# Antibacterial Potential of Symmetrical Twin-Drug 3,6-Diaminoxanthones

**DOI:** 10.3390/ph17020209

**Published:** 2024-02-06

**Authors:** Diana I. S. P. Resende, Fernando Durães, Sidika Zubarioglu, Joana Freitas-Silva, Nikoletta Szemerédi, Madalena Pinto, Eugénia Pinto, Paulo Martins da Costa, Gabriella Spengler, Emília Sousa

**Affiliations:** 1 Laboratory of Organic and Pharmaceutical Chemistry (LQOF), Department of Chemical Sciences, Faculty of Pharmacy, University of Porto, Rua de Jorge Viterbo Ferreira, 228, 4050-313 Porto, Portugal; 2 Interdisciplinary Centre of Marine and Environmental Research (CIIMAR), Terminal de Cruzeiros do Porto de Leixões, Av. General Norton de Matos s/n, 4450-208 Matosinhos, Portugal; 3 ICBAS—Instituto de Ciências Biomédicas Abel Salazar, University of Porto, Rua de Jorge Viterbo Ferreira, 228, 4050-313 Porto, Portugal; 4 Department of Medical Microbiology, Albert Szent-Györgyi Health Center and Albert Szent-Györgyi Medical School, University of Szeged, Semmelweis utca 6, 6725 Szeged, Hungary; 5 Laboratory of Microbiology, Department of Biological Sciences, Faculty of Pharmacy, University of Porto, Rua de Jorge Viterbo Ferreira, 228, 4050-313 Porto, Portugal

**Keywords:** efflux pump, multidrug resistance, xanthones, antibacterial activity, biofilm inhibition, quorum sensing

## Abstract

Global health faces a significant issue with the rise of infectious diseases caused by bacteria, fungi, viruses, and parasites. The increasing number of multi-drug resistant microbial pathogens severely threatens public health worldwide. Antibiotic-resistant pathogenic bacteria, in particular, present a significant challenge. Therefore, there is an urgent need to identify new potential antimicrobial targets and discover new chemical entities that can potentially reverse bacterial resistance. The main goal of this research work was to create and develop a library of 3,6-disubstituted xanthones based on twin drugs and molecular extension approaches to inhibit the activity of efflux pumps. The process involved synthesizing 3,6-diaminoxanthones through the reaction of 9-oxo-9*H*-xanthene-3,6-diyl bis(trifluoromethanesulfonate) with various primary and secondary amines. The resulting 3,6-disubstituted xanthone derivatives were then tested for their in vitro antimicrobial properties against a range of pathogenic strains and their efficacy in inhibiting the activity of efflux pumps, biofilm formation, and quorum-sensing. Several compounds have exhibited effective antibacterial properties against the Gram-positive bacterial species tested. Xanthone **16**, in particular, has demonstrated exceptional efficacy with a remarkable MIC of 11 µM (4 µg/mL) against reference strains *Staphylococcus aureus* ATCC 25923 and *Enterococcus faecalis* ATCC 29212, and 25 µM (9 µg/mL) against methicillin-resistant *S. aureus* 272123. Furthermore, some derivatives have shown potential as antibiofilm agents in a crystal violet assay. The ethidium bromide accumulation assay pinpointed certain compounds inhibiting bacterial efflux pumps. The cytotoxic effect of the most promising compounds was examined in mouse fibroblast cell line NIH/3T3, and two monoamine substituted xanthone derivatives with a hydroxyl substituent did not exhibit any cytotoxicity. Overall, the nature of the substituent was critical in determining the antimicrobial spectra of aminated xanthones.

## 1. Introduction

The consistent growth of antimicrobial resistance has hindered the effective prevention and treatment of an ever-increasing range of infections caused by bacteria, parasites, viruses, and fungi [1,2]. Current medications are losing efficacy, and while infections persist in the body, the risk of spreading to others increases and creates a progressively severe risk for global public health [3,4]. To alleviate this burden, it is imperative to identify new agents that can prevent resistance and shorten treatment duration [4,5].

Xanthones are a class of organic compounds that occur naturally as secondary metabolites in various species of terrestrial and marine plants, fungi, and lichen [6,7]. These heterocyclic polyphenolic compounds with several naturally and synthetically occurring derivatives exhibited significant bactericidal and fungicidal properties against a variety of bacterial and fungal strains [8,9]. In recent works, we described the synthesis and antimicrobial profile of two series of novel nature-inspired xanthones [10,11]. Our group was able to further explore the multidrug resistance reversing activities of twenty xanthone derivatives in *Staphylococcus aureus* 272123 and in the *acrA* gene-inactivated mutant *Salmonella enterica* serovar Typhimurium SL1344 [12]. Their efflux pump inhibitory properties, inhibition of biofilm formation, and quorum sensing studies were evaluated, with two simple aminated derivatives emerging as hit compounds, not only for their overall results against bacterial resistance and virulence mechanisms but also for their lack of cytotoxicity mammalian cells, with potential to be used as safe antibiotic adjuvants [12]. While biofilms play a critical role in the persistence of infections and act as a major source of bacterial dissemination to other areas of the body [13], other mechanisms are responsible for microbial resistance [14]. Quorum sensing (QS) is a process that allows bacteria to communicate with each other and synchronize their gene expression, resulting in optimum effect of secreted proteins [15]. Efflux pumps (EPs) are biological mechanisms that enhance bacterial resistance by expelling antimicrobial factors, QS signals and precursors, and extracellular polymeric substances used to form biofilms. EPs are considered key biological contributors to the development of antimicrobial resistance and are therefore studied as hallmarks of this phenomenon. This first disclosure of xanthones as antimicrobial adjuvants, mainly by aminated derivatives [11] along with the results obtained for a series of hydroxylated xanthones with synergy with different classes of antimicrobials [12] prompted us to further investigate their antimicrobial potential.

In an attempt to gain further insight into the potential of aminated xanthones with different substitution patterns against antimicrobial resistance mechanisms, new molecular modifications to the xanthone scaffold were planned in the present work. A library of 3,6-disubstituted xanthones was investigated for its antimicrobial activity as well as its inhibition of the activity of efflux pumps, biofilm formation, and quorum-sensing (QS). The working hypothesis was the design of symmetrical derivatives that could combine two pharmacophoric units in a single molecule—so-called ‘twin drugs’ aimed at increasing interaction points with biological target(s) and providing desired multiple or complementary modes of action [16,17]. In this study we have applied this approach derivatizing the 3,6-position of the xanthone scaffold that would also allow a molecular extension with bis-derivatives. Thus, in view of the diverse antimicrobial activity of xanthones and in continuation of our work in disclosing aminated derivatives as antimicrobial agents, we herein report for the first time a library of aminated 3,6-substituted xanthones with structural (stereo) diversity (piperidine, *N*,*N*-diethylmethylamine, morpholine, piperazine, pyrrolidine, (4-chlorophenyl)methanamine, (*R*)-1-(4-chlorophenyl)ethan-1-amine, and (*S*)-1-(4-chlorophenyl)ethan-1-amine) that will allow tunability of the pKas of their conjugate bases and the study of structure–activity relationships.

## 2. Results and Discussion

### 2.1. Docking Studies

To investigate the potential for efflux pump activity inhibition of the new library of aminated xanthones, docking studies were performed in the crystal structure of the AcrB portion of the AcrAB-TolC efflux system (PDB: 4DX5), as this is the efflux pump component [18,19]. The experiments were conducted at two distinct locations: the substrate-binding site (SBS) and the hydrophobic trap (HT). The results are presented in Table 1.

All compounds presented docking scores similar or better than the controls and previously obtained hits and, therefore, this library was chosen to undergo in vitro analysis using models of bacteria that have demonstrated resistance. It could also be noted that the compounds appeared to have a better affinity towards the SBS than the HT, which can be explained by the latter being a smaller pocket into which the compounds present herein do not fit [20]. It is also interesting to note that the mono- and disubstituted compounds have similar docking scores, with the exception of the pairs of compounds **8** and **9**, which present very different results for the SBS.

Another interesting observation is the fact that compounds **13**–**18**, which present similarities between themselves, are not predicted to have very different affinities to the SBS of the AcrB component. In fact, a previous virtual screening by our group focused on xanthones with diverse substituents identified two xanthones possessing the same amine as compounds **13**–**18**. The results obtained showed similar docking scores, even though only the xanthone containing the same substituent as **16** was active against the Gram-negative model tested [12].

### 2.2. Synthesis

To find ideal compounds with favourable selectivity and functionality, a ‘twin drug’ approach was adopted as an alternative optimization strategy for the previously synthesized biologically active xanthones [12]. The integration of two pharmacophoric entities within a single molecule enhances the number of interaction points with biological target(s), thus leading to a better on-/off-target profile [16,17]. In addition, our analysis accounted for the fact that the presence of halogens or aminated derivatives along with the presence of hydroxyl groups emphasized their potential as antimicrobial adjuvants [12].

**Scheme 1:**
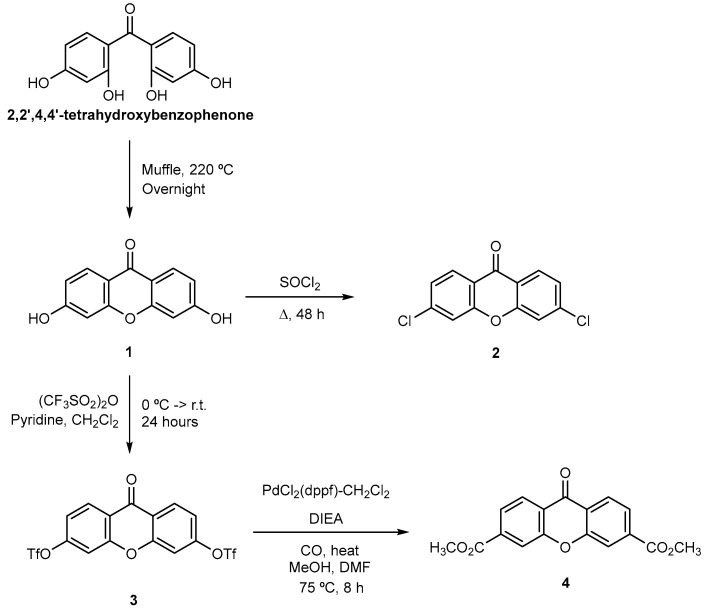
Synthesis of xanthone **1** from 2,2′,4,4′-tetrahydroxybenzophenone, of xanthone **3** to be used as precursor of the aminated xanthones, and of simple xanthones **2** and **4**. DIEA: *N*,*N*-Diisopropylethylamine; DMF: *N*,*N*-Dimethylformamide.

Eighteen 3,6-disubstituted xanthones (**2**–**19**), including fifteen aminated xanthones, were prepared from 3,6-dihydroxy-9*H*-xanthen-9-one (**1**). 3,6-Dihydroxy-9*H*-xanthen-9-one (**1**) was obtained through a dehydrative cyclization of commercially available 2,2′,4,4′-tetrahydroxybenzophenone—a straightforward methodology since no purification is needed to furnish **1** in quantitative yields, as previously reported elsewhere [21]. Based on previous results for antibacterial simple xanthones [10,11], simple derivatives **2** and **4** were also planned for further biological investigations and to clarify the impact of the substituted aminated moieties. 3,6-Dichloro-9*H*-xanthen-9-one (**2**) was obtained from **1** in 81% yield by reaction with thionyl chloride [21] (Scheme 1); for more details see Supplementary Material. The reaction of **1** with trifluoromethanesulfonic anhydride in dichloromethane, via formation of a pyridinium salt, furnishes 9-oxo-9*H*-xanthene-3,6-diyl bis(trifluoromethanesulfonate) **3** in 85% yield [22], which after Pd-catalyzed carbonylation, gives the desired dimethyl 9-oxo-9*H*-xanthene-3,6-dicarboxylate **4** [23]. 

Aminated xanthones **5**–**19** were prepared via reaction of ditriflyl xanthone **3** with the appropriate amines (Table 2) [22]. All the reactions succeeded, with the formation of the 3,6-diaminated xanthone in moderate to good yields (13–82%). Interestingly, in the reaction where the obtained yield was lower, the formation of a hydroxylated product (**7**, **9**, **12**, **14**, **16**, and **18**) was observed, probably due to the unwanted presence of water in the reaction media. Among all the synthesized derivatives, compounds **7**, **9**, **12**–**19** are described herein for the first time. The structure elucidation of compounds **1**–**6**, **8**, **10**, and **11** was established by ^1^H and ^13^C nuclear magnetic resonance (NMR) techniques, and the acquired data are in accordance with those previously reported [21,22,23]. The structure elucidation of new compounds **7**, **9**, **12**–**19** was established on the basis of high-resolution mass spectrometry (HRMS) and NMR techniques; full details are provided in the Supplementary Material [22]. The purity of the compounds was assessed by HPLC and shown to be higher than 95%.

### 2.3. Antimicrobial Activity

Xanthones **1**–**9**, **11**–**14**, and **19** were not active against any of the six tested bacteria strains—three Gram-positive (*Staphylococcus aureus* ATCC 25923, *Enterococcus faecalis* ATCC 29212, and *S. aureus* 272123) and three Gram-negative (*Escherichia coli* ATCC 25922, *Pseudomonas aeruginosa* ATCC 27853, and *S. enterica* serovar Typhimurium SL1344). Nonetheless, some compounds presented interesting antibacterial activity. This is the case of **10**, the only xanthone derivative with activity against a Gram-negative strain (*E. coli* ATCC 25922) (Table 3). This result might be related to the highest pKa value of amine **10**, hypothesized to be in an ionizable state in high amounts, thus facilitating compound’s entry through Gram negative porins. Some derivatives were found to be active against Gram-positive strains tested, as can be seen in Table 3. It is worth noting the results obtained for derivative **16**, which displayed a MIC of 11 µM (4 µg/mL) for the reference strains *S. aureus* ATCC 25923 and *E. faecalis* ATCC 29212, and of 25 µM (9 µg/mL) against the oxacillin- and methicillin-resistant *S. aureus* 272123. 

Mono-derivative **16** and its enantiomer **18** revealed the highest effect, being active against three different Gram-positive bacterial strains and presenting higher antibacterial activity than the corresponding symmetric **15** and **17** derivatives. These results highlight the relevance of the 1-(4-chlorophenyl)ethan-1-amine chain with stereochemistry effects not being determinant for activity. Moreover, for growth inhibitory effects, twin-drug and/or molecular extension approaches were not beneficial. It can also be inferred that the presence of the substituent at position 3 of the xanthone core favors antibacterial activity when compared to xanthones with an identical substituent at position 1 [11]. We hypothesize that derivative **10**, the only derivative active against Gram-negative bacteria, being the most basic compound (pKa = 8.507), may penetrate better through porins and reach the growth inhibitory target in higher concentrations. In vitro antifungal activity of 19 synthesized xanthones (**1**–**19**) was tested against three fungal strains, but none showed activity against *Candida albicans*, *Aspergillus fumigatus*, or *Trichophyton rubrum* except for xanthone **10,** which showed a MIC value for *T. rubrum* of 508 μM (256 µg/mL).

### 2.4. Efflux Pump Activity Inhibition Assay

The compounds were assessed for their capability of modulating ethidium bromide (EB) accumulation in resistant *S. aureus* and *S. typhimurium* SL1344 strains by the automated EB method. A relative fluorescence index was calculated based on the means of relative fluorescence units, as can be seen in Table 4.

From the analysis shown in the previous table, it can be noted that **5**, **7**, **12**, **13**, **16**, **18**, and **19** can increase fluorescence (values highlighted in bold) in comparison to the positive control (reserpine and CCCP), which can be attributed to the inhibition of the efflux of EB in the tested strains but can also be due to the fluorescence emitted by the compound itself. As such, the fluorescence of these compounds was measured in a solution of PBS for the duration of the assay (60 min).

The results obtained showed that the fluorescence of the compound itself was not an issue in the case of **5**. However, some compounds displayed erratic fluorescence curves, and their results for this particular assay were not considered; this happened for **12**, **13** and **16**. Compounds **7**, **18**, and **19** were not tested, as their fluorescence was only high in one of the strains tested. Genetic assays could be performed as an alternative to real-time ethidium bromide accumulation to overcome the problem of fluorescence of the compounds.

As such, it could be highlighted that compound **7** was effective at inhibiting efflux pumps in *S. aureus* 272123, while compounds **18** and **19** displayed the same effects on *S. enterica* Typhimurium SL1344, and compound **5** was effective against both strains. Compared with previously analyzed hydroxylated [12], aminated [12], and amidated [24] xanthones, we can conclude that this approach benefits EPI effects, with the most potent EPI xanthones being disclosed in this work. As in previous studies [12] and contrary to growth inhibitory studies, the stereochemistry of the substituents is determinant for EPI.

The compounds with potential to inhibit the activity of efflux pumps such as **5**, **7**, **18**, and **19** were evaluated for the inhibition of resistance mechanisms closely linked to the inhibition of EPs, specifically biofilm formation and QS.

### 2.5. Inhibition of Biofilm Formation and Quorum-Sensing Assays

Efflux pumps are essential components that affect the formation of biofilms and QS. They act as mediators in the removal of polymeric substances that contribute to biofilm production and also export the signal molecules of QS that regulate biofilm formation. These factors play a crucial role in determining how bacteria gather and stick to solid surfaces. Additionally, the influence of Eps on QS signal molecules can also have a significant impact on the overall process of QS itself. Due to these relationships, the effect of xanthones **5**, **7**, **18**, and **19** and efflux pump activity on biofilm formation was examined for the reference strain *S. aureus* ATCC 25923 and the resistant strain *S. aureus* 272123. Biofilm inhibition, presented as a %, was calculated based on the mean of absorbance units. Reserpine was used as a control in both strains, as this was the positive control used in the real-time EB accumulation assay and has also been described as an anti-biofilm agent [25]. The results obtained are present in Table 5.

Since biofilms are closely linked to EPs, as these structures are responsible for the transport of extracellular polymeric substances that ultimately lead to the production of biofilm, it was expected that the compounds that produced a reduction in the efflux of ethidium bromide would show reduction in biofilm formation [14]. This relationship, however, could not be observed in the results obtained, as xanthone derivative **19** was the only one to demonstrate significant activity in *S. aureus* ATCC 25923 (56%) and an inhibition of approximately 94% in *S. aureus* 272123. This compound did not show an inhibition of efflux activity greater than reserpine in the real-time ethidium bromide accumulation assay, but was effective against *S. enterica* Typhimurium SL1344, which could mean that it disrupts biofilm formation through a mechanism other than the inhibition of efflux pump activity.

As QS is a virulence mechanism dependent on the segregation of signal molecules to the extracellular medium, the inhibition of the transport of these molecules, which also happens through EPs, could result in a lack of communication through QS [26]. In order to assess this, three models were used: a sensor strain *Chromobacterium violaceum* 026 and an AHL producer strain *Sphingomonas paucimobilis* Ezf **10**–**17**, inoculated as parallel lines, AHL producer *C. violaceum* wild type 85 (wt85), and *Serratia marcescens* AS-1, the latter two inoculated as single lines. The interaction between strains and compounds was evaluated as the reduction of pigment production, in millimeters. Promethazine (PMZ) was used as a positive control, and compounds **5**, **7**, **18**, and **19** were evaluated (Table 6).

Compounds **5** and **19** showed potential in inhibiting QS and can also inhibit the efflux of ethidium bromide in *S. typhimurium* SL1344, the Gram-negative strain previously tested, which may suggest a possible connection, as the bacteria tested herein are also Gram-negative and have been described to express RND efflux pumps [27]. Compound **7**, which was able to inhibit efflux in the Gram-positive strain, was also effective at inhibiting QS, suggesting a possible link between these effects exerted by compound **7**.

### 2.6. Cytotoxicity Assay

When selecting efflux pump inhibitors for therapeutics, it is crucial to consider several factors. Three significant aspects to keep in mind include: selecting an inhibitor that is not antibacterial to prevent resistance, ensuring that the molecule is selective and does not affect eukaryotic efflux pumps, and confirming that it does not pose a risk of toxicity to eukaryotic cells. In order to evaluate the toxicity of the most promising compounds that showed positive results in biofilm inhibition (**5**, **7**, **18**, and **19**), efflux pump inhibition, and/or QS inhibition assays, a simple toxicity test was carried out on normal mouse fibroblast cells (NIH/3T3). Doxorubicin was used as a positive control. The results are shown in Table 7, and the dose response curves are presented as supplementary data (Figure S2).

Within the series of 3,6-disubstituted xanthones, it can be noted that derivatives **5** and **19** presented low cytotoxicity towards the tested cell line. Interestingly, monoamine substituted xanthone derivatives with a hydroxyl substituent (**7** and **18**) did not display cytotoxicity. The cytotoxicity displayed by compounds **5** and **19** may not compromise the effectivity of these compounds as efflux pump inhibitors (EPIs), since they were active below their IC_50_. 

## 3. Materials and Methods

### 3.1. Chemistry

#### 3.1.1. Reagents and Equipment

All reagents and solvents were purchased from TCI (Tokyo, Japan), Acros, Sigma Aldrich (Sigma-Aldrich Co., Ltd., Gillinghan, UK), or Alfa Aesar and had no further purification process. A Buchi Waterchath B-480 rotary evaporator was used to remove solvents under reduced pressure. A Köfler (Wagner and Munz, Munich, Germany) microscope was used to measure melting points (m.p.), which were uncorrected. The structure elucidation of the compounds was achieved using NMR spectra, which were obtained by the Department of Chemistry of the University of Aveiro at room temperature using a Bruker Advance 300 spectrometer (300 or 400 MHz for ^1^H and 75 or 101 MHz for ^13^C, Bruker Biosciences Corporation, Billerica, MA, USA). Carbons were assigned using HSQC and HMBC spectra. HRESIMS analyses were performed at CIIMAR, Matosinhos, using a Q Exactive Focus Hybrid Quadrupole Orbitrap Mass Spectrometer (Thermo Fisher Scientific, Bremen, Germany) controlled by Q Exactive Focus (Exactive Series) 2.9 and Thermo Scientific Xcalibur 4.1.31.9 software. HRESIMS data were obtained in full scan positive and negative mode with a scan range of *m*/*z* 100–1500, the capillary voltage of HESI set to −3.5 kV, and the capillary temperature set to 263 °C. The sheath gas flow rate was set to 50 units. 

#### 3.1.2. Synthesis

##### General

Pre-coated thin-layer chromatography plates (0.2-mm thickness using Merck silica gel 60 (GF_254_)) were used to monitor the progress of the reactions. The plates were eluted with the appropriate mobile phases and further visualization was performed using a UV light source suitable for observations under short (254 nm) and long (365 nm) wavelength UV light. Flash column chromatography was used to purify the obtained compounds (Merck silica gel 60 (0.040–0.063 mm, Merck, Darmstadt, Germany) and preparative thin layer chromatography (silica gel HPLC60 RP-18 (GF_254_), Merck, Darmstadt, Germany). Determination of the compound’s purity by HPLC-DAD was performed by Dr. Sara Cravo at Laboratory of Organic and Pharmaceutical Chemistry. For compounds **13**, **14**, **15**, **17**, and **18**, the HPLC system consisted of a Shimadzu LC-20AD pump equipped with a Shimadzu DGV-20A5 degasser, a Rheodyne 7725i injector fitted with a 20 µL loop, and an SPD-M20A DAD detector (Kyoto, Japan). Data acquisition was performed using Shimadzu LCMS Lab Solutions software, version 3.50 SP2. The column used in this study was an ACE—C18 (150 mm × 4.6 mm I.D., particle size 5 µm) (Advanced Chromatography Technologies Ltd. (Aberdeen, Scotland, UK)). The mobile phase composition was water and methanol (2:8 *v*/*v*); all were HPLC grade solvents obtained from Merck Life Science S.L.U. (Darmstadt, Germany). The flow rate was 1.0 mL/min, and the UV detection wavelength was 254 nm. Analyses were performed at 28 °C in an isocratic mode in a 30 min run. Peak purity index was determined by total peak UV-Vis spectra between 210–800 nm with a step of 4 nm. For compounds **7**, **9**, **12**, **16**, and **19**, the HPLC system consisted of a Shimadzu LC-20AD pump equipped with a Shimadzu DGV-20A5 degasser, a Rheodyne 7725i injector fitted with a 20 µL loop, and an SPD-M20A DAD detector (Kyoto, Japan). Data acquisition was performed using Shimadzu LCMS Lab Solutions software, version 3.50 SP2 (Kyoto, Japan). The column used in this study was Phenomenex–Lux^®^ 5 µm Amylose-1 (250 mm × 4.6 mm I.D., particle size 5 µm) manufactured by Phenomenex, Inc. (Torrance, CA, USA). Analyses were performed using a mixture of HPLC grade solvents *n*-hexane and ethanol (8:2 *v*/*v*) as the mobile phase (Carlo Erba, Reagents S.r.l. (Val de Reuil, France)) under the following conditions: flow rate of 1.0 mL/min, UV detection wavelength: 254 nm, room temperature, isocratic mode, 20 to 60 min run.

##### Synthesis of Xanthone Primary Derivatives **1**–**4**

Xanthones **1**, **2**, **3**, and **4** were synthesized and characterized according to previously described procedures [21,22,23], respectively.

##### Synthesis of Aminated Xanthones **5**–**19**

9-Oxo-9*H*-xanthene-3,6-diyl bis(trifluoromethanesulfonate) was dissolved in DMSO (0.2 M) and the appropriate amine (10 equiv.) was added. The reaction mixture was heated to 90 °C and stirred for 20 h. After this period, the solvent was removed, and the reaction crude purified by preparative chromatography (CH_2_Cl_2_/Acetone 100:2 as mobile phase) to give the pure products.

**3,6-di(piperidin-1-yl)-9*H*-xanthen-9-one (5)**: Compound **5** was synthesized (150.9 mg, 82%) and characterized according to the previously described procedure [22].

**3,6-bis(diethylamino)-9H-xanthen-9-one (6)**: Compound **6** was synthesized (49.8 mg, 29%) and characterized according to the previously described procedure [22].

**3-(diethylamino)-6-hydroxy-9H-xanthen-9-one (7)**: Light yellow solid (71.9 mg, 50%). m.p. 182–184 °C. ^1^H NMR (400 MHz, DMSO-d_6_, δ ppm): 7.94 (d, ^3^*J*_1,2_ = 8.6 Hz, 1H, H-1), 7.88 (d, ^3^*J*_8,7_ = 9.1 Hz, 1H, H-8), 6.82 (dd, ^3^*J*_2,1_ = 8.7 Hz, ^4^*J*_2,4_ = 2.3 Hz, 1H, H-2), 6.78 (d, ^4^*J*_4,2_ = 2.2 Hz, 1H, H-4), 6.77 (dd, ^3^*J*_7,8_ = 8.7 Hz, ^4^*J*_7,5_ = 2.3 Hz, 1H, H-7), 6.54 (d, ^4^*J*_5,7_ = 2.4 Hz, 1H, H-5), 3.45 (q, ^3^*J*_1′,2′_ = ^3^*J*_1′′,2′′_ = 7.0 Hz, 4H, H-1′a, H-1′b, H-1′’a, H-1′’b), 1.14 (t, ^3^*J*_2′,1′_ = ^3^*J*_2′′,1′′_ = 7.0 Hz, 6H, H-2′a, H-2′b, H-2′c, H-2′′a, H-2′′b, H-2′′c). ^13^C NMR (101 MHz, DMSO-d_6_, δ ppm): 173.3 (C-9), 162.8 (C-3), 158.0 (C-10a), 157.3 (C-4a), 152.2 (C-6), 127.5 (C-1), 127.3 (C-8), 114.3 (C-9a), 113.1 (C-2), 110.0 (C-8a), 109.3 (C-7), 102.0 (C-4), 95.9 (C-5), 44.1 (C-1′, C-1′′), 12.3 (C-2′, C-2′′). HRMS (ESI, negative ions) *m*/*z*: [M-H]^−^ calculated for C_17_H_16_NO_3_^−^: 282.11247, found 282.11380.

**3,6-dimorpholino-9*H*-xanthen-9-one (8)**: Compound **8** was synthesized (41.4 mg, 18%) and characterized according to the previously described procedure [22].

**3-hydroxy-6-morpholino-9*H*-xanthen-9-one (9)**: Light yellow solid (89.9 mg, 52%). m.p. 169–171 °C. ^1^H NMR (400 MHz, DMSO-d_6_, δ ppm): 7.96 (d, ^3^*J*_1,2_ = 8.7 Hz, 1H, H-1), 7.93 (d, ^3^*J*_8,7_ = 9.0 Hz, 1H, H-8), 7.05 (dd, ^3^*J*_7,8_ = 9.1 Hz, ^4^*J_7_*_,5_ = 2.4 Hz, 1H, H-7), 6.87 (d, ^4^*J*_5,7_ = 2.4 Hz, 1H, H-5), 6.84 (dd, ^3^*J*_2,1_ = 8.7 Hz, ^4^*J_2_*_,4_ = 2.2 Hz, 1H, H-2), 6.79 (d, ^4^*J*_4,2_ = 2.2 Hz, 1H, H-4), 3.73 (m, 4H, H-2′a, H-2′b, H-6′a, H-6′b), 3.37 (m, 4H, H-3′a, H-3′b, H-5′a, H-5′b). ^13^C NMR (101 MHz, DMSO-d_6_, δ ppm): 173.6 (C-9), 163.21 (C-3), 157.5 (C-4a, C-10a), 155.3 (C-6), 127.6 (C-1), 126.9 (C-8), 114.2 (C-2), 113.4 (C-8a), 112.4 (C-9a), 111.3 (C-7), 102.0 (C-4), 99.4 (C-5), 65.8 (C-3′,5′), 46.8 (C-2′,6′). HRMS (ESI, negative ions) *m*/*z*: [M-H]^−^ calculated for C_17_H_16_NO_4_^−^: 296.09173, found 296.09320.

**3,6-di(piperazin-1-yl)-9*H*-xanthen-9-one (10):** Compound **10** was synthesized (118.4 mg, 64%) and characterized according to the previously described procedure [22].

**3,6-di(pyrrolidin-1-yl)-9*H*-xanthen-9-one (11):** Compound **11** was synthesized (40.7 mg, 24%) and characterized according to the previously described procedure [22].

**3-hydroxy-6-(pyrrolidin-1-yl)-9*H*-xanthen-9-one (12):** Light yellow solid (32.9 mg, 23%). m.p. 176–178 °C. ^1^H NMR (300 MHz, DMSO-d_6_, δ ppm): 7.93 (d, ^3^*J*_1,2_ = 8.7 Hz, 1H, H-1), 7.89 (d, ^3^*J*_8,7_ = 8.9 Hz, 1H, H-8), 6.80 (dd, ^3^*J*_2,1_ = 8.6 Hz, ^4^*J_2_*_,4_ = 2.2 Hz, 1H, H-2), 6.76 (d, ^4^*J*_4,2_ = 2.1 Hz, 1H, H-4), 6.64 (dd, ^3^*J*_7,8_ = 8.9 Hz, ^4^*J*_7,5_ = 2.2 Hz, 1H, H-7), 6.42 (d, ^4^*J*_5,7_ = 2.2 Hz, 1H, H-5), 3.36 (t, ^3^*J*_2′,3′_ = ^3^*J*_5′,4′_ = 6.5 Hz, 4H, H-2′a, H-2′b, H-5′a, H-5′b), 1.98 (t, ^3^*J*_3′,2′_ = ^3^*J*_3′,4′_ = ^3^*J*_4′,5′_ = ^3^*J*_4′,3′_ = 6.5 Hz, 4H, H-3′a, H-3′b, H-4′a, H-4′b). ^13^C NMR (75 MHz, DMSO-d_6_, δ ppm): 173.4 (C-9), 163.4 (C-3), 157.6 (C-10a), 157.3 (C-4a), 151.8 (C-6), 127.5 (C-1), 127.1 (C-8), 114.0 (C-2), 113.3 (C-9a), 110.2 (C-7), 110.0 (C-8a), 101.9 (C-4), 96.4 (C-5), 47.5 (C-2′, C-5′), 25.0 (C-3′, C-4′). HRMS (ESI, positive ions) *m*/*z*: [M + H]^+^ calculated for C_17_H_16_NO_3_^+^: 282.11247, found 282.11230.

**3,6-bis((4-chlorobenzyl)amino)-9*H*-xanthen-9-one (13):** Light yellow solid (115.9 mg, 48%). m.p. 171–173 °C. ^1^H NMR (300 MHz, DMSO-d_6_, δ ppm): 7.76 (d, ^3^*J*_1,2_ = ^3^*J*_8,7_ = 8.7 Hz, 2H, H-1, H-8), 7.36–7.46 (m, 8H, H-2′, H-3′, H-5′, H-6′, H-2′’, H-3′’, H-5′’, H-6′’), 6.67 (dd, ^3^*J*_2,1_ = ^3^*J*_7,8_ = 8.8 Hz and ^4^*J_2_*_,4_ = ^4^*J*_7,5_ = 2.2 Hz, 2H, H-2, H-7), 6.32 (d, ^4^*J*_4,2_ = ^4^*J*_5,7_ = 2.1 Hz, 2H, H-4, H-5), 4.38 (d, ^3^*J* = 5.9 Hz, 4H, CH_2_). ^13^C NMR (75 MHz, DMSO-d_6_, δ ppm): 173.0 (C-9), 157.7 (C-4a, C-10a), 153.6 (C-3, C-6), 138.2 (C-1′, C-1′′), 131.5 (C-4′, C-4′′), 129.0 (C-2′, C-6′, C-2′′, C-6′′), 128.5 (C-3′, C-5′, C-3′, C-5′′), 126.7 (C-1, C-8), 111.2 (C-2, C-7, C-8a, C-9a), 95.8 (C-4, C-5), 45.2 (CH_2_). HRMS (ESI, positive ions) *m*/*z*: [M + H]^+^ calculated for C_27_H_21_Cl_2_N_2_O_2_^+^: 475.09746, found 475.10193.

**3-((4-chlorobenzyl)amino)-6-hydroxy-9*H*-xanthen-9-one (14):** Light yellow solid (51.8 mg, 29%). m.p. 162–164 °C. ^1^H NMR (300 MHz, DMSO-d_6_, δ ppm): 7.92 (d, ^3^*J*_1,2_ = 8.2 Hz, 1H, H-1), 7.81 (d, ^3^*J*_8,7_ = 8.1 Hz, 1H, H-8), 7.54–7.62 (m, 1H, NH), 7.40 (br s, 4H, H-2′, H-3′, H-5′, H-6′), 6.82 (d, ^3^*J*_2,1_ = 8.7 Hz, 1H, H-2), 6.77 (s, 1H, H-4), 6.73 (d, ^3^*J*_7,8_ = 8.8 Hz, 1H, H-7), 6.40 (s, 1H, H-5), 4.41 (d, ^3^*J* = 5.9 Hz, 4H, CH_2_). ^13^C NMR (75 MHz, DMSO-d_6_, δ ppm): 173.3 (C-9), 162.9 (C-3), 158.0 (C-10a), 157.2 (C-4a), 154.0 (C-6), 138.1 (C-1′), 131.5 (C-4′), 129.1 (C-2′,6′/C-3′,5′), 128.5 (C-2′,6′/C-3′,5′), 127.5 (C-1), 126.9 (C-8), 114.2 (C-9a), 113.2 (C-2), 110.9 (C-7), 102.0 (C-8a), 99.6 (C-4), 95.8 (C-5), 45.1 (CH_2_). HRMS (ESI, positive ions) *m*/*z*: [M + H]^+^ calculated for C_20_H_15_ClNO_3_^+^: 352.07350, found 352.07290.

**3,6-bis(((*R*)-1-(4-chlorophenyl)ethyl)amino)-9*H*-xanthen-9-one (15):** Light yellow solid (33.2 mg, 13%). m.p. 159–161 °C. ^1^H NMR (300 MHz, DMSO-d_6_, δ ppm): 7.71 (d, ^3^*J*_1,2_ = ^3^*J*_8,7_ = 8.8 Hz, 2H, H-1, H-8), 7.36–7.43 (m, 4H, H-2′, H-3′, H-5′, H-6′), 6.67 (dd, ^3^*J*_2,1_ = ^3^*J*_7,8_ = 8.8 Hz and ^4^*J_2_*_,4_ = ^4^*J*_7,5_ = 2.1 Hz, 2H, H-2, H-7), 6.14 (d, ^4^*J*_4,2_ = ^4^*J*_5,7_ = 2.1 Hz, 2H, H-4, H-5), 4.64 (t, ^3^*J* = 6.8 Hz, 2H, CH). 1.44 (d, ^3^*J* = 6.8 Hz, 3H, CH_3_).^13^C NMR (75 MHz, DMSO-d_6_, δ ppm): 172.9 (C-9), 157.5 (C-4a, C-10a), 152.8 (C-3, C-6), 144.1 (C-1′), 131.2 (C-4′), 128.5 (C-3′, C-5′), 127.8 (C-2′, C-6′), 126.5 (C-1, C-8), 111.7 (C-8a, C-9a), 111.1 (C-2, C-7), 96.3 (C-4, C-5), 51.2 (CH), 24.2 (CH_3_). HRMS (ESI, positive ions) *m*/*z*: [M + H]^+^ calculated for C_29_H_25_Cl_2_N_2_O_2_^+^: 503.12876, found 503.13382.

**(*R*)-3-((1-(4-chlorophenyl)ethyl)amino)-6-hydroxy-9*H*-xanthen-9-one (16):** Light yellow solid (57.6 mg, 31%). m.p. 187–188 °C. ^1^H NMR (300 MHz, DMSO-d_6_, δ ppm): 10.69 (s, 1H, OH), 7.91 (d, ^3^*J*_8,7_ = 8.7 Hz, 1H, H-8), 7.78 (d, ^3^*J*_1,2_ = 8.8 Hz, 1H, H-1), 7.52–7.32 (m, 5H, H-2′, H-3′, H-5′, H-6′, NH), 6.80 (dd, ^3^*J*_7,8_ = 8.7 Hz and ^4^*J*_7,5_ = 2.2 Hz, 1H, H-7), 6.73 (d, ^4^*J_4,2_* = 2.2 Hz, 1H, H-4), 6.71 (dd, ^3^*J*_2,1_ = 9.5 Hz and ^4^*J*_2,4_ = 2.2 Hz, 1H, H-2), 4.68 (t, *J* = 6.7 Hz, 1H, CH), 1.45 (d, *J* = 6.7 Hz, 3H, CH_3_). ^13^C NMR (75 MHz, DMSO-d_6_, δ ppm): 173.3 (C-9), 162.8 (C-6), 157.8 (C-4a), 157.2 (C-10a), 153.2 (C-3), 143.9 (C-1′), 131.3 (C-4′), 128.6, 127.8 (C-2′,6′/C-3′,5′), 127.5 (C-8), 126.8 (C-1), 114.2 (C-7), 113.2 (C-8a), 112.0 (C-9a), 110.9 (C-2), 102.0 (C-5), 96.3 (C-4), 51.2 (CH), 24.1 (CH_3_). HRMS (ESI, positive ions) *m*/*z*: [M + H]^+^ calculated for C_21_H_17_ClNO_3_^+^: 364.07350, found 364.07520.

**3,6-bis(((*S*)-1-(4-chlorophenyl)ethyl)amino)-9*H*-xanthen-9-one (17):** Light yellow solid (58.8 mg, 23%). m.p. 182–184 °C. ^1^H NMR (300 MHz, DMSO-d_6_, δ ppm): 7.99 (d, ^3^*J*_1,2_ = ^3^*J*_8,7_ = 8.7 Hz, 2H, H-1, H-8), 7.37–7.21 (m, 8H, H-2′, H-3′, H-5′, H-6′, H-2′′, H-3′′, H-5′’, H-6′′), 6.52 (dd, ^3^*J*_2,1_ = ^3^*J*_7,8_ = 8.7 Hz and ^4^*J_2_*_,4_ = ^4^*J*_7,5_ = 2.2 Hz, 2H, H-2, H-7), 6.11 (d, ^4^*J*_4,2_ = ^4^*J*_5,7_ = 2.2 Hz, 2H, H-4, H-5), 4.68 (d, ^3^*J* = 5.3 Hz, 2H, NH_a_, NH_b_), 4.63–4.44 (m, 2H, CH_a_,CH_b_), 1.53 (d, *J* = 6.7 Hz, 6H, CH_3_a, CH_3_b).^13^C NMR (75 MHz, DMSO-d_6_, δ ppm): 173.9 (C-9), 159.4 (C-4a, C-10a), 152.7 (C-3, C-6), 143.6 (C-4′, C-4′′), 134.4 (C-1′, C-1′′), 129.9 (CAr), 127.8 (C-1, C-8), 127.3 (CAr), 114.1 (C-8a, C-9a), 109.8 (C-2, C-7), 98.4 (C-4, C-5), 52.2 (CH_a_, CH_b_), 24.9 (CH_3_a, CH_3_b). HRMS (ESI, positive ions) *m*/*z*: [M + H]^+^ calculated for C_29_H_25_Cl_2_N_2_O_2_^+^: 503.12876, found 503.13617.

**(*S*)-3-((1-(4-chlorophenyl)ethyl)amino)-6-hydroxy-9*H*-xanthen-9-one (18):** Light yellow solid (65.0 mg, 35%). m.p. 196–198 °C. ^1^H NMR (300 MHz, DMSO-d_6_, δ ppm): 7.89 (d, ^3^*J*_8,7_ = 8.7 Hz, 1H, H-8), 7.78 (d, ^3^*J*_1,2_ = 8.8 Hz, 1H, H-1), 7.51–7.31 (m, 4H, H-2′, H-3′, H-5′, H-6′), 6.78 (dd, ^3^*J*_7,8_ = 8.7 Hz and ^4^*J*_7,5_ = 2.2 Hz, 1H, H-7), 6.73–6.69 (m, 2H, H-2, H-5), 6.27 (br s, 1H, H-4), 4.73–4.64 (m, 1H, NH), 4.05–3.99 (m, 1H, CH), 1.46 (d, J = 6.7 Hz, 3H, CH_3_). ^13^C NMR (75 MHz, DMSO-d_6_, δ ppm): 173.7 (C-9), 164.3 (C-6), 158.3 (C-4a), 157.7 (C-10a), 153.6 (C-3), 147.4 (C-1′), 144.4 (C-4′), 131.7 (C-8a/C-9a), 131.2 (C-8a/C-9a), 129.0 (C-Ar), 128.4 (C-Ar), 128.3 (C-Ar), 128.3 (C-Ar), 127.9 (C-8), 127.2 (C-a), 114.2 (C-7), 111.4 (C-2), 102.4 (C-5), 96.7 (C-4), 50.4 (CH), 26.8 (CH_3_). HRMS (ESI, positive ions) *m*/*z*: [M + H]^+^ calculated for C_21_H_17_ClNO_3_^+^: 366.08915, found 366.09365.

**3,6-bis(5-amino-3,4-dihydroisoquinolin-2(1*H*)-yl)-9*H*-xanthen-9-one (19):** Light yellow solid (64.5 mg, 26%). m.p. 193–194 °C. ^1^H NMR (300 MHz, DMSO-d_6_, δ ppm): 7.90 (d, ^3^*J*_1,2_ = ^3^*J*_8,7_ = 9.0 Hz, 2H, H-1, H-8), 7.08 (dd, ^3^*J*_2,1_ = ^3^*J*_7,8_ = 9.1 Hz and ^4^*J_2_*_,4_ = ^4^*J*_7,5_ = 2.3 Hz, 2H, H-2, H-7), 6.89 (t, ^3^*J* = 7.7 Hz, 2H, H-7′, H-7′′), 6.83 (d, ^4^*J*_4,2_ = ^4^*J*_5,7_ = 2.3 Hz, 2H, H-4, H-5), 6.50 (t, ^3^*J* = 6.7 Hz, 4H, H-6′, H-8′, H-6′′, H-8′′), 4.92 (s, 4H, 5′-NH_2_, 5′’-NH_2_), 4.51 (s, 4H, H-1′a, H-1′b, H-1′′a, H-1′′b), 3.76 (t, ^3^*J* = 5.9 Hz, 4H, H-3′a, H-3′b, H-3′’a, H-3′’b), 2.62 (t, ^3^*J* = 5.8 Hz, 4H, H-4′a, H-4′b, H-4′′a, H-4′′b). ^13^C NMR (75 MHz, DMSO-d_6_, δ ppm): 173.2 (C-9), 157.7 (C-4a, C-10a), 154.0 (C-3, C-6), 146.0 (C-5′, C-5′′), 134.2 (C-8a′, C-8a′′), 126.9 (C-1, C-8), 126.3 (C-7′, C-7′′), 118.4 (C-4a′, C-4a′′), 114.4 (C-8′, C-8′′), 112.3 (C-6′, C-6′′), 111.8 (C-8a, C-9a), 110.9 (C-2, C-7), 98.6 (C-4, C-5), 49.2 (C-1′, C-1′′), 44.7 (C-3′, C-3′′), 22.8 (C-4′, C-4′′). HRMS (ESI, positive ions) *m*/*z*: [M + H]^+^ calculated for C_31_H_29_N_4_O_2_^+^: 489.22850, found 489.22824.

### 3.2. Microbiology

#### 3.2.1. Culture Media and Chemicals

In the experiments described herein, the culture media used were the following: cation-adjusted Mueller–Hinton broth (MHB II; Sigma-Aldrich, St. Louis, MO, USA and Biokar Diagnostics, Allone, Beauvais, France), Luria–Bertani broth (LB-B; Sigma, St. Louis, MO, USA), Tryptic Soy broth (TSB; Scharlau Chemie S. A., Barcelona, Spain), and Trypto-Casein Soy agar (TSA; Biokar Diagnostics, Allone, Beauvais, France). For the QS inhibition assays, the agar medium used was Luria–Bertani with a few modifications (LB*-A) and was prepared in house. The composition was as follows: 1.0 g yeast extract (Merck, Darmstadt, Germany), 10.0 g tryptone (Biolab, Budapest, Hungary), 10.0 g NaCl (Molar Chemicals, Halásztelek, Hungary), 1.0 g K_2_HPO_4_ (Biolab, Budapest, Hungary), 0.3 g MgSO_4_·7 H_2_O (Reanal, Budapest, Hungary), 5 mL Fe-EDTA stock solution and 20.0 g of bacteriological agar (Molar Chemicals, Halásztelek, Hungary) per liter of media.

The chemicals used—DMSO, 3-(4,5-dimethylthiazol-2-yl)-2,5-diphenyltetrazolium bromide (MTT), sodium dodecyl sulfate (SDS), phosphate-buffered saline (PBS; pH 7.4), ethidium bromide (EB), reserpine, carbonyl cyanide 3-chlorophenylhydrazone (CCCP), PMZ, PAβN, and CV—were bought from Sigma-Aldrich Chemie GmbH (Steinheim, Germany), and doxorubicin (2 mg/mL) was purchased from Teva Pharmaceuticals, Budapest, Hungary.

#### 3.2.2. Microorganisms

The bacterial strains used were *Staphylococcus aureus* American Type Culture Collection (ATCC) 25923, *Enterococcus faecalis* ATCC 29212, and methicillin and ofloxacin-resistant *Staphylococcus aureus* 272123 clinical isolate (Gram-positive), *Escherichia coli* ATCC 25922*, Pseudomonas aeruginosa* ATCC 27853, and *acrA* gene-inactivated mutant *Salmonella enterica* serovar Typhimurium SL1344 (Gram-negative). Regarding the QS tests, all the strains used were Gram-negative, and comprised *Chromobacterium violaceum* wild type 85 (wt85), *C. violaceum* CV026 (CV026), *Sphingomonas paucimobilis* Ezf 10–17 (EZF), and *Serratia marcescens* AS-1. The antifungal activity of the compounds was evaluated against *Candida albicans* ATCC 10231, *Aspergillus fumigatus* ATCC 204305, *Trichophyton rubrum* FF5.

#### 3.2.3. Microbiological Analysis of Xanthone Derivatives

Xanthone derivatives were evaluated for their minimum inhibitory concentration (MIC), efflux pump inhibition (EPI) activity, influence on biofilm formation, quorum-sensing (QS) inhibition, and cytotoxicity. Docking studies were also performed. The compounds were dissolved in DMSO to obtain a stock solution of 10 mg/mL. Scheme 1 and Table 2 shows the structures of the derivatives used.

#### 3.2.4. Docking Studies

The crystal structure of AcrB (PDB code: 4DX5) [18] was downloaded from the protein databank (PDB) [28]. Previously reported AcrB inhibitors D13-9001 doxorubicin, MBX-3132, minocycline, and PAβN were drawn along with the tested compounds with ChemDraw (PerkinElmer Informatics), and their energy was minimized using ArgusLab. Docking studies were carried out using AutoDock Vina (Scripps, CA, USA) [29] for the sites described in [20], which were the SBS and the HT. For each molecule, the top nine poses were collected, and the most favorable binding conformation was that which presented the lowest docking score.

#### 3.2.5. Antibacterial Assay

The MIC of the compounds was determined using the microdilution method in a 96-well plate following the guidelines established by the Clinical and Laboratory Standard Institute (CLSI) [30]. Herein, MHB II was used as culture media, and the compounds were tested in concentrations ranging from 64 µg/mL to 4 µg/mL, with MIC being determined by visual inspection. As the compounds were diluted from a stock solution in DMSO, we made sure that this solvent was used in subinhibitory concentrations (1% (*v*/*v*)).

#### 3.2.6. Antifungal Activity

The synthesized compounds were evaluated against the yeast *C. albicans*, the filamentous fungus *A. fumigatus*, and the dermatophyte *T. rubrum*. Similar to the antibacterial assay, the microdilution method was used to determine the MIC following CLSI guidelines (reference documents M27-A3 for yeasts [31] and M38-A2 [32] for filamentous fungi). In brief, suspensions of cells or spores were prepared in RPMI-1640 broth medium (Biochrom, Berlin, Germany), which was supplemented with MOPS (Sigma-Aldrich, St. Louis, MO, USA), starting from fresh cultures of the different strains of fungi, and the inoculum was adjusted. In the same medium, two-fold serial dilutions of the compounds, ranging from 4–256 µg/mL, were prepared. The DMSO concentration did not exceed 2.5% (*v*/*v*). MIC was considered to be the lowest concentration at which there was complete growth inhibition in comparison to the controls with no compound. The quality control was assured using the MIC of voriconazole for *Pichia kudriavzevii* (former *Candida krusei*) ATCC 6258 [31,32].

#### 3.2.7. Efflux Pump Inhibition Assays

The compounds were tested for their ability to inhibit the activity of efflux pumps in the strains *S. typhimurium* SL1344 and *S. aureus* 272123, using real-time fluorimetry through the monitorization of the intracellular accumulation of the efflux pump substrate EB. An automated method was used using a CLARIOstar Plus plate reader (BMG Labtech, Ortenberg, Germany). The positive controls were reserpine for Gram-positive strains and CCCP for Gram-negative strains, which were applied at 25 µM, and the solvent DMSO was also applied at a subinhibitory concentration of 1% (*v*/*v*).

The bacterial strains were incubated in an appropriate culture media, which was TSB for *S. aureus* 272123 and LB-B for *S. typhimurium* SL1344, at 37 °C, until the optical density (OD) of the inoculate was between 0.4 and 0.6 at 600 nm. A centrifugation of the culture at 13,000× *g* took place for 3 min, followed by a washing step and resuspension in PBS (pH 7.4). The centrifugation and resuspension steps were repeated.

In this assay, the compounds were applied at a concentration equivalent to one-third of their MIC in a 1 µg/mL solution of EB in PBS, as this concentration was safe for the bacteria in use. The exceptions were compounds with a MIC higher than 100 µM, where the tested concentration was 50 µM. A volume of 50 µL of this solution was transferred into a 96-well black microtiter plate (Greiner Bio-One Hungary Kft, Mosonmagyaróvár, Hungary), and 50 µL of bacterial suspension (OD_600_ 0.4–0.6) was added to each well.

The CLARIOstar plate reader was used to monitor the fluorescence at excitation and emission wavelengths of 530 nm and 600 nm, respectively, on a real-time basis, every minute for an hour. From this data, the inhibitory activity of the compounds, namely the RFI at minute 60, which was the last time point of this assay, was calculated using the formula:

$$RFI=\frac{{RF}_{treated}-{RF}_{untreated}}{{RF}_{untreated}}$$

where RF_treated_ is the relative fluorescence (RF) at the last time point of the EB accumulation curve in the presence of the compound, and RF_untreated_ is the RF at the last time point of the EB accumulation curve of the untreated control (the well containing only DMSO).

#### 3.2.8. Inhibition of Biofilm Formation

The ability of selected compounds to decrease the formation of biofilm was also studied in two strains of *S.* aureus: *S. aureus* ATCC 25923 and *S. aureus* 272123. Biofilm formation was detected using crystal violet (CV), a dye, at 0.1% (*v*/*v*). The initial inoculum was incubated overnight in TSB, and diluted to an OD_600_ of 0.1. Then, the bacterial suspensions were transferred to 96-well microtiter plates, and the compounds were added at a subinhibitory concentration (half the MIC or 100 µM if the compound did not present an observable MIC for these strains in the previous assay) to a final volume of 200 µL. The EPI reserpine served as the positive control.

The plates were incubated for 48 h at 30 °C with stirring at 100 rpm. When this incubation period was over, the incubation medium was disposed of, and the unattached cells were removed by rinsing the plate with tap water. Then, a volume of 200 µL of a 0.1% (*v*/*v*) CV solution was added, followed by a 15 min incubation at room temperature, after which the CV solution was discarded, and the plates rinsed tap water again. Finally, 200 µL of ethanol (70%) were added to the wells.

The OD_600_ was measured using a Multiscan EX ELISA plate reader (Thermo Labsystems, Cheshire, WA, USA), and the effect of the compounds in the formation of biofilm was expressed in percentage (%) of decrease in biofilm formation.

#### 3.2.9. Quorum Sensing Assay

The inhibition of QS by these compounds was investigated on the EZF and sensor CV026 pair, CV026wt, and *S. marcescens*. The parallel inoculation method was used, which consisted of pair combinations of the sensor strain CV026 and EZF, a strain capable of producing *N*-acyl-homoserine lactone (AHL), were inoculated directly onto the LB*-A surface as parallel lines, 5 mm from each other, approximately. The other strains, *S. marcescens* AS-1 and wt85, were inoculated as single lines. In the center of the inoculated line(s), filter paper disks (7 mm in diameter) impregnated with 8 µL of a solution of 10 mM of the compounds were placed. As positive control, the drug PMZ was used.

The plates were incubated for 24–48 h at room temperature (20 °C), after which the inhibition of pigment production was assessed visually as an indicator of QS inhibition. The discoloured but intact bacterial colonies were measured with a ruler [33].

#### 3.2.10. Cell Lines and Cultures

The cell line used was NIH/3T3, ATCC CRL-1658TM, cultivated in DMEM (Gibco 52100-039) supplemented with 10% heat-inactivated fetal bovine serum (Biowest, VWR International Kft, Debrecen, Hungary), 100 U/L of L-glutamine, 1% Na pyruvate, 1% penicillin/streptomycin (Sigma-Aldrich Chemie GmbH), and 0.1% nystatin (8.3 g/L in ethylene glycol). Using a combination of 0.25% trypsin and 0.02% EDTA for 5 min at 37 °C, the adherent cells were detached. Before each cytotoxicity assay, the cells were seeded in untreated 96-well flat-bottom microtiter plates, followed by a 4-h incubation period in a humidified atmosphere (5% CO_2_, 95% air) at 37 °C [34].

#### 3.2.11. Cytotoxicity Assay

The cytotoxicity of the tested compounds was assessed in mouse embryonic fibroblasts (NIH/3T3 cells). The cells were distributed in 96-well flat-bottom microtiter plates at a concentration of 1 × 10^4^, to which the compounds were added to. Initially, they were incubated for 24 h, which was followed by the addition of a solution of MTT in PBS to each well, followed by a 4-h incubation period. A volume of 100 μL of a solution of 10% SDS in a 0.01 M HCl solution was added, and incubated the cells were incubated overnight at 37 °C. As a positive control, doxorubicin was used.

Cellular growth was determined in quadruplicate by measuring the OD at 540 nm (reference 630 nm) using a Multiscan EX ELISA reader (Thermo Labsystems, Cheshire, WA, USA). The inhibition of cell growth was determined using the following equation:

$$100-\left(\frac{{OD}_{sample}-{OD}_{mediumcontrol}}{{OD}_{cellcontrol}-{OD}_{mediumcontrol}}\right)\times 100$$

The results were obtained as the mean ± standard deviation (SD), and the IC_50_ values were calculated by best fitting the dose-dependent inhibition curves in GraphPad Prism 5.03 for Windows software.

## 4. Conclusions

Increasing levels of drug resistance in our society have made developing new antimicrobial agents a crucial need. The present study delves into various efflux pump-related aspects of bacterial resistance, including the compounds’ overall activity and their interaction with different virulence and resistance determinants such as bacterial communication, biofilm formation, and efflux pump inhibition. The study reinforces the potential of xanthone derivatives in overcoming antimicrobial resistance mechanisms. The ‘twin drug’ approach proved to be a successful strategy for the development of new xanthone derivatives to be used as antimicrobial adjuvants.

Eighteen 3,6-disubstituted xanthones were obtained from 3,6-dihydroxy-9*H*-xanthen-9-one (**1**) via several synthetic methodologies. Aminated xanthones **5**–**19** were prepared via reaction of ditriflyl xanthone **3** with the appropriate amines and, to the best of our knowledge, xanthones **7**, **9**, and **12**–**19** were synthesized in this study for the first time. A set of 3,6-disubstituted xanthones showed their potential as antimicrobials and as antibiotic adjuvants. 

Compound **16** was identified as the hit compound in terms of antibacterial activity as it presented inhibitory action against the growth of three Gram-positive bacterial strains. For inhibiting of efflux pumps, we hypothesized in previous studies that an aminated substituent may cause membrane perturbations that can change their function. The structural similarity of xanthones and phenothiazines, previously reported as EPIs [35], reinforce the idea that xanthones may be potential inhibitors of this target. Compounds **5**, **7**, **18**, and **19** were effective at decreasing the efflux of ethidium bromide in the tested strains, which can translate to the inhibition of EPs. The ability of the compounds to inhibit mechanisms related to EPs, such as biofilm formation and QS, was also investigated. Amongst the compounds tested, only xanthone derivative **19** showed significant anti-biofilm activity in *S. aureus* ATCC 25923 (56%) and inhibited approximately 94% of *S. aureus* 272123. Although this compound did not demonstrate greater inhibition of efflux activity than reserpine in the real-time ethidium bromide accumulation assay, it was found to be effective against *S. enterica* Typhimurium SL1344. This suggests that it disrupts biofilm through a mechanism other than the inhibition of EPs. Compounds **5** and **19** have the potential to inhibit QS and ethidium bromide efflux in Gram-negative strains, highlighting the importance of the piperidine ring present in both compounds. Compound **7** inhibits QS and efflux in Gram-positive strains. The structural relationship between the tested xanthone derivatives and promethazine, the positive control, may contribute to QS inhibition. Some of the 3,6-disubstituted xanthone derivatives presented cytotoxic effects on the tested cell line, but those with hydroxyl or monoamine substitutions did not display cytotoxicity. Even though compounds **5** and **19** displayed cytotoxicity on the cell line used, the concentrations at which they display this effect is higher than what was used for the efflux pump inhibition assay. This means they can still be used effectively and safely as adjuvants.

In conclusion, this work resulted in the synthesis of a small library of twin 3,6-disubstituted xanthones and confirmed that xanthone derivatives present a promising and innovative avenue for the production of novel antibacterial agents as well as for the development of compounds capable of combatting common resistance mechanisms.

The overall results show that the synthesized xanthone derivatives not only are effective in inhibiting efflux pumps but also present efficacy against other resistance mechanisms with different hits found in different experiments. While **18** presented a broader range against other resistance mechanisms and the capacity to inhibit Gram-positive bacteria not allied with toxicity; **7** also presented a broader range and no toxicity but was not capable of inhibiting either Gram-positive or Gram-negative bacteria. The interesting profile of these derivatives opens the possibility of their use as antimicrobials or in the circumvention of resistance mechanisms. Further research will be carried out to evaluate the biological impacts of the synthesized compounds. It would be worthwhile to delve into the precise mechanisms by which the compounds exert their action or the efflux pumps to which they bind in greater detail through comprehensive studies.

## Figures and Tables

**Table 1:** Docking results for the compounds in AcrB.

Compound	Docking Score		Compound	Docking Score	
	SBS	HT		SBS	HT
**1**	−6.8	−6.1	**13**	−9.1	5.0
**2**	−6.7	−5.4	**14**	−8.4	−5.4
**3**	−9.9	−6.6	**15**	−9.2	15.0
**4**	−7.2	−5.5	**16**	−8.3	−0.2
**5**	−8.7	−4.3	**17**	−9.4	12.8
**6**	−6.7	−4.2	**18**	−8.2	−2.9
**7**	−6.8	−5.4	**19**	−10.4	11.8
**8**	−9.0	−3.9	**D13-9001**	−9.7	26.5
**9**	−7.6	−6.2	**Doxorubicin**	−8.9	15.4
**10**	−8.6	−3.6	**MBX-3132**	−7.9	2.9
**11**	−8.2	−5.2	**Minocycline**	−8.7	26.7
**12**	−7.5	−6.0	**PAβN**	−7.1	−4.7

SBS: Substrate binding site; HT: Hydrophobic trap. PAβN: Phe-Arg-β-naphthylamide.

**Table 2:**
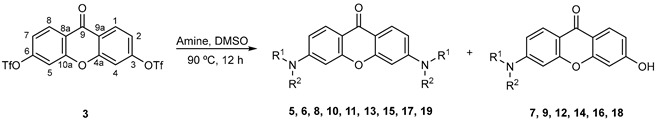
Synthesis of aminated xanthones **5**–**19**.

Entry	Compound	Yield, %	Compound	Yield, %
1	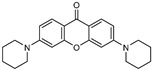	**5** (82)	-	-
2	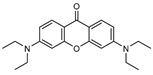	**6** (29)	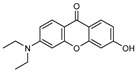	**7** (50)
3	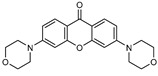	**8** (18)	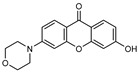	**9** (52)
4	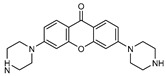	**10** (64)	-	-
5	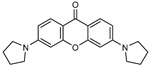	**11** (24)	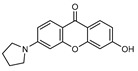	**12** (23)
6	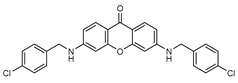	**13** (48)	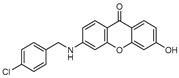	**14** (29)
7	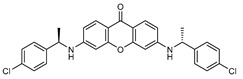	**15** (13)	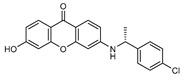	**16** (31)
8	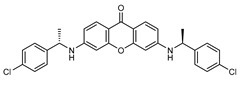	**17** (23)	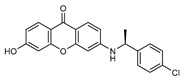	**18** (35)
9	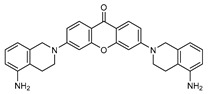	**19** (26)	-	-

- The corresponding mono derivatives of twin drugs **5**, **10** and **19** could not be isolated/detected.

**Table 3:** MIC values for xanthones **1**–**4**, **10,** and **15**–**18** in μM (μg/mL) against a panel of Gram-negative and Gram-positive bacteria.

Compound	MIC in µM (µg/mL)			
	*E. coli* ATCC 25922	*S. aureus* ATCC 25923	*S. aureus* 272123	*E. faecalis* ATCC 29212
**1–4**	>200	>200	>200	>200
**10**	176 (64 µg/mL)	>200	>200	>200
**15**	>200	>200	>200	127 (64 µg/mL)
**16**	>200	11 (4 µg/mL)	25 (9 µg/mL)	11 (4 µg/mL)
**17**	>200	>200	100 (50 µg/mL)	127 (64 µg/mL)
**18**	>200	44 (16 µg/mL)	50 (18 µg/mL)	22 (8 µg/mL)

Gram-negative bacteria (*E. coli* ATCC 25922) and Gram-positive bacteria (*S. aureus* ATCC 25923, *S. aureus* 272123, and *E. faecalis* ATCC 29212).

**Table 4:** Relative fluorescence index of tested derivatives **1**–**19** observed for Gram-positive *S. aureus* 272123 and Gram-negative *S. enterica* Typhimurium SL1344.

Relative Fluorescence Index (RFI)			Relative Fluorescence Index (RFI)		
Compound	*S. aureus* 272123	*S. enterica* Typhimurium SL1344	Compound	*S. aureus* 272123	*S. enterica* Typhimurium SL1344
**1**	−0.23 ± 0.04	−0.17 ± 0.02	**12**	**0.88 ± 0.07**	**2.24 ± 0.05**
**2**	−0.09 ± 0.04	0.02 ± 0.04	**13**	**2.12 ± 0.07**	**3.52 ± 0.64**
**3**	−0.05 ± 0.07	0.09 ± 0.02	**14**	0.06 ± 0.05	0.17 ± 0.03
**4**	−0.21 ± 0.04	−0.05 ± 0.03	**15**	−0.08 ± 0.09	0.06 ± 0.02
**5**	**1.12 ± 0.02**	**0.82 ± 0.02**	**16**	**0.74 ± 0.01**	**4.30 ± 0.01**
**6**	0.32 ± 0.05	0.04 ± 0.04	**17**	0.19 ± 0.01	−0.06 ± 0.02
**7**	**0.76 ± 0.04**	0.28 ± 0.14	**18**	0.36 ± 0.05	**0.57 ± 0.11**
**8**	0.12 ± 0.03	0.03 ± 0.08	**19**	−0.27 ± 0.04	**0.49 ± 0.04**
**9**	−0.58 ± 0.06	−0.26 ± 0.10	**Reserpine**	0.50 ± 0.04	-
**10**	0.06 ± 0.07	−0.11 ± 0.02	**CCCP**	-	0.40 ± 0.07
**11**	−0.01 ± 0.18	0.01 ± 0.06			

CCCP: Carbonyl cyanide 3-chlorophenylhydrazone; values highlighted in bold represent an RFI above the positive control.

**Table 5:** Percentage of biofilm inhibition by the selected compounds **5**, **7**, **18**, and **19** against Gram-positive *S. aureus* ATCC 25923 and *S. aureus* 272123.

Compound	Inhibition of Biofilm Formation (%)	
	*S. aureus* ATCC 25923	*S. aureus* 272123
**5**	0	13.1 ± 2.65
**7**	0	8.16 ± 0.93
**18**	0	47.1 ± 0.04
**19**	56.1 ± 0.05	93.6 ± 0.03
**Reserpine**	22.3 ± 5.10	63.1 ± 2.33

**Table 6:** Results of the quorum sensing inhibition assay for compounds **5**, **7**, **18**, and **19**.

Compound	Quorum Sensing Inhibition (mm)		
	*S. marcescens*	wt85	EZF + CV026
**5**	0	0	21 ± 0.5
**7**	0	0	53 ± 0.1
**18**	0	0	59 ± 0.5
**19**	0	0	29 ± 0.5
**PMZ**	18 ± 0.8	40 ± 0.1	41 ± 0.5

**Table 7:** IC_50_ values (μM and µg/mL) of **5**, **7**, **18**, and **19** in cytotoxicity assays against NIH/3T3 mouse fibroblast cells.

Compound	IC_50_ (µM) ± SD	IC_50_ (µg/mL) ± SD
**5**	82.03 ± 0.27	29.73 ± 0.10
**7**	>100	>28.31
**18**	>100	>36.58
**19**	64.22 ± 1.09	31.38 ± 0.53
**Doxorubicin**	12.05 ± 0.81	6.55 ± 0.44

## Data Availability

The data presented in this study are available as Supplementary Materials.

## Supplementary Materials

The following supporting information can be downloaded at: https://www.mdpi.com/article/10.3390/ph17020209/s1, NMR data (Figures S1–S40), HRMS data (Figures S41–S50), and HPLC-DAD Purity Data (Table S1).

## Abbreviations

AHL	Acyl-homoserine-lactone
ATCC	American Type Culture Collection
CCCP	Carbonyl cyanide 3-chlorophenylhydrazone
CV026	*Chromobacterium violaceum* CV026
CV	Crystal violet
DMSO	Dimethyl sulfoxide
EPIs	efflux pump inhibitors
EB	Ethidium bromide
EZF	*Sphingomonas paucimobilis* EZF 10-17
HT	Hydrophobic trap
IC_50_	Half-maximal inhibitory concentration
LB-A	Luria–Bertani agar
LB-B	Luria–Bertani broth
LD	Lipoyl domain
MFS	Major facilitator superfamily
MHB II	Cation-adjusted Mueller–Hinton broth
MIC	Minimum inhibitory concentration
MTT	3-(4,5-dimethylthiazol-2-yl)-2,5-diphenyltetrazolium bromide
OD	Optical density
PBS	Phosphate-buffered saline
PMZ	Promethazine
QS	Quorum sensing
RF	Relative fluorescence
RFI	Relative fluorescence index
SBS	Substrate-binding site
SD	Standard deviation
TSA	Tryptic Soy agar
TSB	Tryptic Soy broth

## References

[1] Picot S., Beugnet F., Leboucher G., Bienvenu A.-L. (2022). Drug Resistant Parasites and Fungi from a One-Health Perspective: A Global Concern That Needs Transdisciplinary Stewardship Programs. One Health.

[2] Verhoef J., van Kessel K., Snippe H., Parnham M.J., Nijkamp F.P., Rossi A.G. (2019). Immune Response in Human Pathology: Infections Caused by Bacteria, Viruses, Fungi, and Parasites. Nijkamp and Parnham’s Principles of Immunopharmacology.

[3] Adebisi Y.A., Ogunkola I.O. (2023). The Global Antimicrobial Resistance Response Effort Must Not Exclude Marginalised Populations. Trop. Med. Health.

[4] Mo Y., Oonsivilai M., Lim C., Niehus R., Cooper B.S. (2023). Implications of Reducing Antibiotic Treatment Duration for Antimicrobial Resistance in Hospital Settings: A Modelling Study and Meta-Analysis. PLoS Med..

[5] Annunziato G. (2019). Strategies to Overcome Antimicrobial Resistance (AMR) Making Use of Non-Essential Target Inhibitors: A Review. Int. J. Mol. Sci..

[6] Badiali C., Petruccelli V., Brasili E., Pasqua G. (2023). Xanthones: Biosynthesis and Trafficking in Plants, Fungi and Lichens. Plants.

[7] Remali J., Sahidin I., Aizat W.M. (2022). Xanthone Biosynthetic Pathway in Plants: A Review. Front. Plant Sci..

[8] Kuete V., Alibert-Franco S., Eyong K.O., Ngameni B., Folefoc G.N., Nguemeving J.R., Tangmouo J.G., Fotso G.W., Komguem J., Ouahouo B.M.W. (2011). Antibacterial Activity of Some Natural Products against Bacteria Expressing a Multidrug-Resistant Phenotype. Int. J. Antimicrob. Agents.

[9] Fotie J., Bohle S.D. (2006). Pharmacological and Biological Activities of Xanthones. Anti-Infect. Agents Med. Chem..

[10] Resende D.I.S.P., Pereira-Terra P., Inácio Â.S., Costa P.M.d., Pinto E., Sousa E., Pinto M.M.M. (2018). Lichen Xanthones as Models for New Antifungal Agents. Molecules.

[11] Resende D.I.S.P., Pereira-Terra P., Moreira J., Freitas-Silva J., Lemos A., Gales L., Pinto E., de Sousa M.E., da Costa P.M., Pinto M.M.M. (2020). Synthesis of a Small Library of Nature-Inspired Xanthones and Study of Their Antimicrobial Activity. Molecules.

[12] Durães F., Resende D.I.S.P., Palmeira A., Szemerédi N., Pinto M.M.M., Spengler G., Sousa E. (2021). Xanthones Active against Multidrug Resistance and Virulence Mechanisms of Bacteria. Antibiotics.

[13] Vestby L.K., Grønseth T., Simm R., Nesse L.L. (2020). Bacterial Biofilm and its Role in the Pathogenesis of Disease. Antibiotics.

[14] Alav I., Sutton J.M., Rahman K.M. (2018). Role of Bacterial Efflux Pumps in Biofilm Formation. J. Antimicrob. Chemother..

[15] Wu L., Luo Y. (2021). Bacterial Quorum-Sensing Systems and Their Role in Intestinal Bacteria-Host Crosstalk. Front. Microbiol..

[16] Tomassoli I., Gündisch D. (2015). The Twin Drug Approach for Novel Nicotinic Acetylcholine Receptor Ligands. Bioorganic Med. Chem..

[17] Fujisaki F., Aki H., Naito A., Fukami E., Kashige N., Miake F., Sumoto K. (2014). Synthesis of New 5-Substituted Hydantoins and Symmetrical Twin-Drug Type Hydantoin Derivatives. Chem. Pharm. Bull..

[18] Eicher T., Cha H.-J., Seeger M.A., Brandstätter L., El-Delik J., Bohnert J.A., Kern W.V., Verrey F., Grütter M.G., Diederichs K. (2012). Transport of Drugs by the Multidrug Transporter Acrb Involves an Access and a Deep Binding Pocket That Are Separated by a Switch-Loop. Proc. Natl. Acad. Sci. USA.

[19] Du D., Wang Z., James N.R., Voss J.E., Klimont E., Ohene-Agyei T., Venter H., Chiu W., Luisi B.F. (2014). Structure of the Acrab–Tolc Multidrug Efflux Pump. Nature.

[20] Aron Z., Opperman T.J. (2018). The Hydrophobic Trap—The Achilles Heel of Rnd Efflux Pumps. Res. Microbiol..

[21] Rosa G.P., Palmeira A., Resende D.I.S.P., Almeida I.F., Kane-Pagès A., Barreto M.C., Sousa E., Pinto M.M.M. (2021). Xanthones for Melanogenesis Inhibition: Molecular Docking and Qsar Studies to Understand Their Anti-Tyrosinase Activity. Bioorganic Med. Chem..

[22] Wu L., Burgess K. (2008). Synthesis and Spectroscopic Properties of Rosamines with Cyclic Amine Substituents. J. Org. Chem..

[23] Dax S., DeCorte B., Liu L., McDonnell M., McNally J. (2009). Tricyclic-Bridged Piperidinylidene Derivatives as δ-Opioid Modulators. U.S. Patent.

[24] Durães F., Cravo S., Freitas-Silva J., Szemerédi N., Martins-da-Costa P., Pinto E., Tiritan M.E., Spengler G., Fernandes C., Sousa E. (2021). Enantioselectivity of Chiral Derivatives of Xanthones in Virulence Effects of Resistant Bacteria. Pharmaceuticals.

[25] Parai D., Banerjee M., Dey P., Mukherjee S.K. (2020). Reserpine Attenuates Biofilm Formation and Virulence of Staphylococcus Aureus. Microb. Pathog..

[26] Kaur B., Gupta J., Sharma S., Sharma D., Sharma S. (2021). Focused Review on Dual Inhibition of Quorum Sensing and Efflux Pumps: A Potential Way to Combat Multi Drug Resistant Staphylococcus Aureus Infections. Int. J. Biol. Macromol..

[27] Benomar S., Evans K.C., Unckless R.L., Chandler J.R. (2019). Efflux Pumps in *Chromobacterium* Species Increase Antibiotic Resistance and Promote Survival in a Coculture Competition Model. Appl. Environ. Microbiol..

[28] Sussman J.L., Lin D., Jiang J., Manning N.O., Prilusky J., Ritter O., Abola E.E. (1998). Protein Data Bank (Pdb): Database of Three-Dimensional Structural Information of Biological Macromolecules. Acta Crystallogr. Sect. D Biol. Crystallogr..

[29] Trott O., Olson A.J. (2010). Autodock Vina: Improving the Speed and Accuracy of Docking with a New Scoring Function, Efficient Optimization, and Multithreading. J. Comput. Chem..

[30] Clinical and Laboratory Standards Institute (2018). Methods for Dilution Antimicrobial Susceptibility Tests for Bacteria That Grow Aerobically.

[31] Clinical and Laboratory Standards Institute (2008). Clinical and Laboratory Standards Institute: Reference Method for Broth Dilution Antifungal Susceptibility Testing of Yeasts.

[32] Clinical and Laboratory Standards Institute (2008). Reference Method for Broth Dilution Antifungal Susceptibility Testing of Filamentous Fungi.

[33] Gajdács M., Spengler G. (2019). The Role of Drug Repurposing in the Development of Novel Antimicrobial Drugs: Non-Antibiotic Pharmacological Agents as Quorum Sensing-Inhibitors. Antibiotics.

[34] Ferreira R.J., Kincses A., Gajdács M., Spengler G., dos Santos D.J.V.A., Molnár J., Ferreira M.-J.U. (2018). Terpenoids from Euphorbia Pedroi as Multidrug-Resistance Reversers. J. Nat. Prod..

[35] Kaatz Glenn W., Moudgal Varsha V., Seo Susan M., Kristiansen Jette E. (2003). Phenothiazines and Thioxanthenes Inhibit Multidrug Efflux Pump Activity in Staphylococcus aureus. Antimicrob. Agents Chemother..

