# Supplementary material for: Antibacterial Potential of Symmetrical Twin-Drug 3,6-Diaminoxanthones
Source: Pharmaceuticals (Basel). 2024 Feb 6;17(2):209. doi: 10.3390/ph17020209 (PMC10891989; doi:10.3390/ph17020209)
Supplement: Supplementary file 1 [file pharmaceuticals-17-00209-s001.zip › pharmaceuticals-2826597-supplementary.pdf]

Supporting information

Antibacterial Potential of Symmetrical Twin-Drug 3,6-Diaminoxanthones

Diana I. S. P. Resende [1,2,†], Fernando Durães [1,2,†], Sidika Zubarioglu [1], Joana Freitas-Silva [2,3], Nikoletta Szemerédi [4], Madalena Pinto [1,2], Eugénia Pinto [2,5,*], Paulo Martins da Costa [2,3], Gabriella Spengler [4] and Emília Sousa [1,2,*]

[1] Laboratory of Organic and Pharmaceutical Chemistry (LQOF), Department of Chemical Sciences, Faculty of Pharmacy, University of Porto, Rua de Jorge Viterbo Ferreira, 228, 4050-313 Porto, Portugal
[2] Interdisciplinary Centre of Marine and Environmental Research (CIIMAR), Terminal de Cruzeiros do Porto de Leixões, Av. General Norton de Matos s/n, 4450-208 Matosinhos, Portugal
[3] ICBAS—Instituto de Ciências Biomédicas Abel Salazar, University of Porto, Rua de Jorge Viterbo Ferreira 228, 4050-313 Porto, Portugal
[4] Department of Medical Microbiology, Albert Szent-Györgyi Health Center and Albert Szent-Györgyi Medical School, University of Szeged, Semmelweis utca 6, 6725 Szeged, Hungary
[5] Laboratory of Microbiology, Department of Biological Sciences, Faculty of Pharmacy, University of Porto, Rua de Jorge Viterbo Ferreira, 228, 4050-313 Porto, Portugal
* Correspondence: epinto@ff.up.pt (E.P.); esousa@ff.up.pt (E.S.); Tel.: +351-220428585 (E.P.); +351-220428689 (E.S.)
† These authors contributed equally to this work.

Contents

1. NMR Spectra 2
2. High-resolution mass spectrometry 22
3. Peak purity 27

1. NMR Spectra

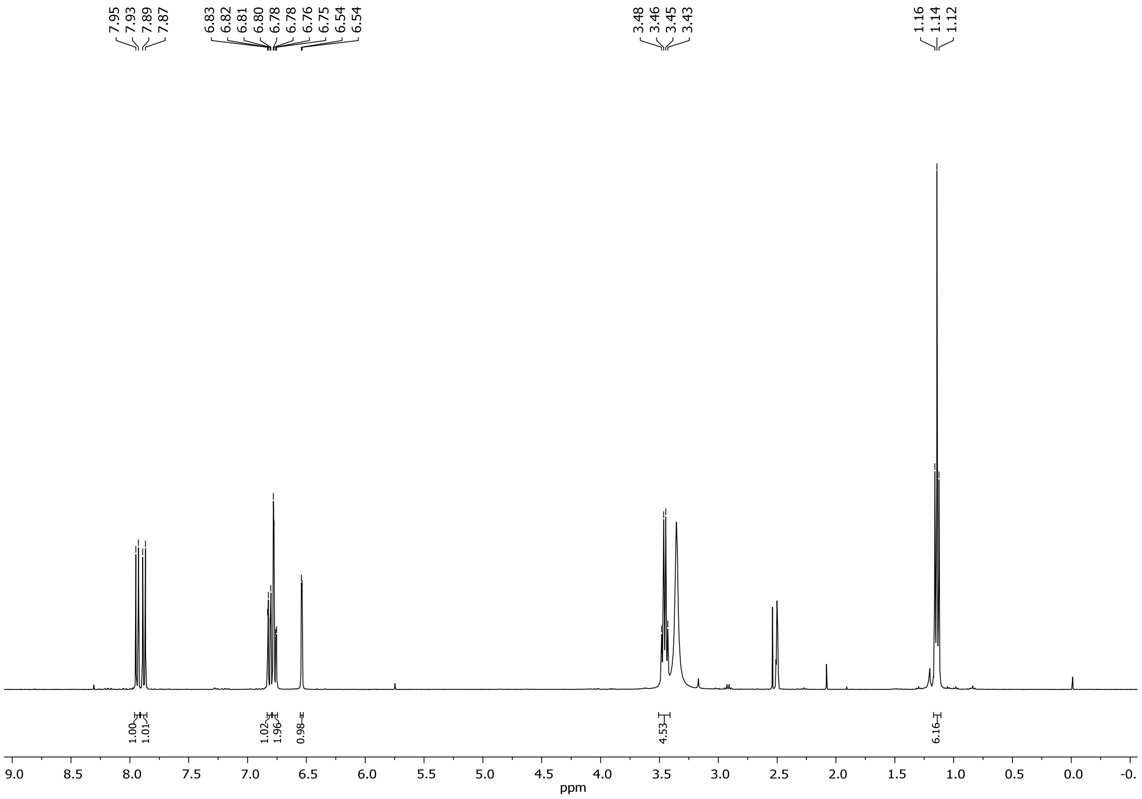

*Figure S1. ^{1}H NMR spectrum of 3-(diethylamino)-6-hydroxy-9H-xanthen-9-one (**7**) (DMSO-d_6, 400 MHz).*

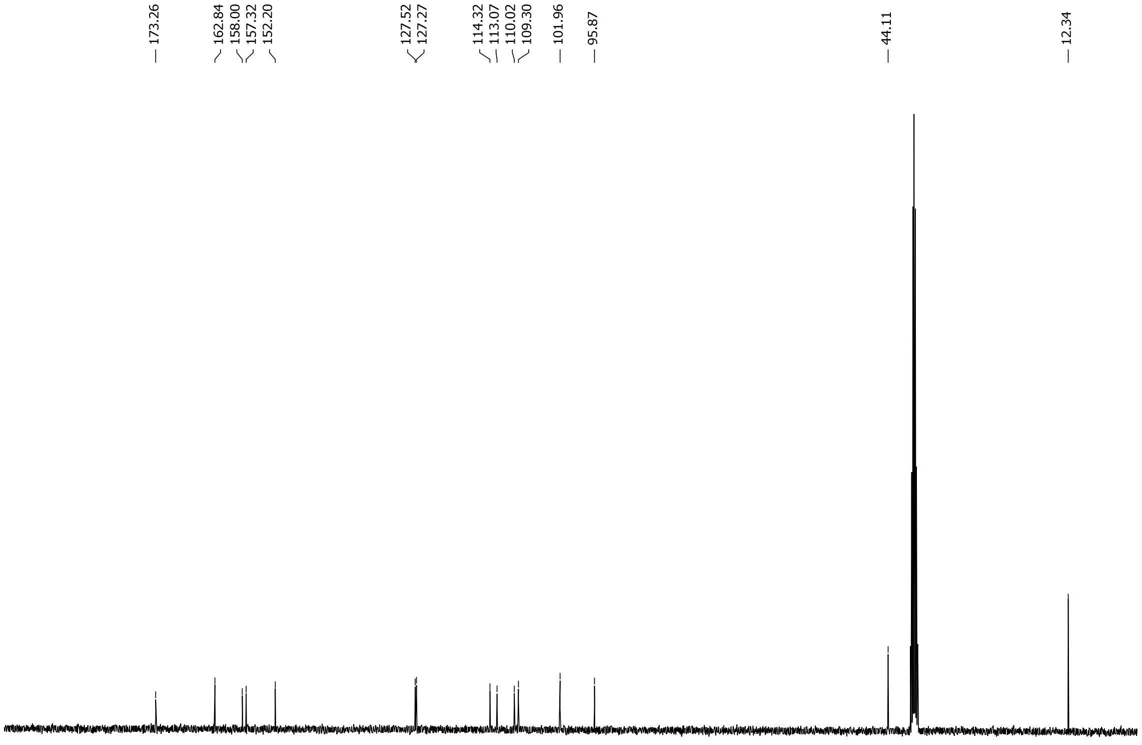

*Figure S2. ^{13}C NMR spectrum of 3-(diethylamino)-6-hydroxy-9H-xanthen-9-one (**7**) (DMSO-d_6, 101 MHz).*

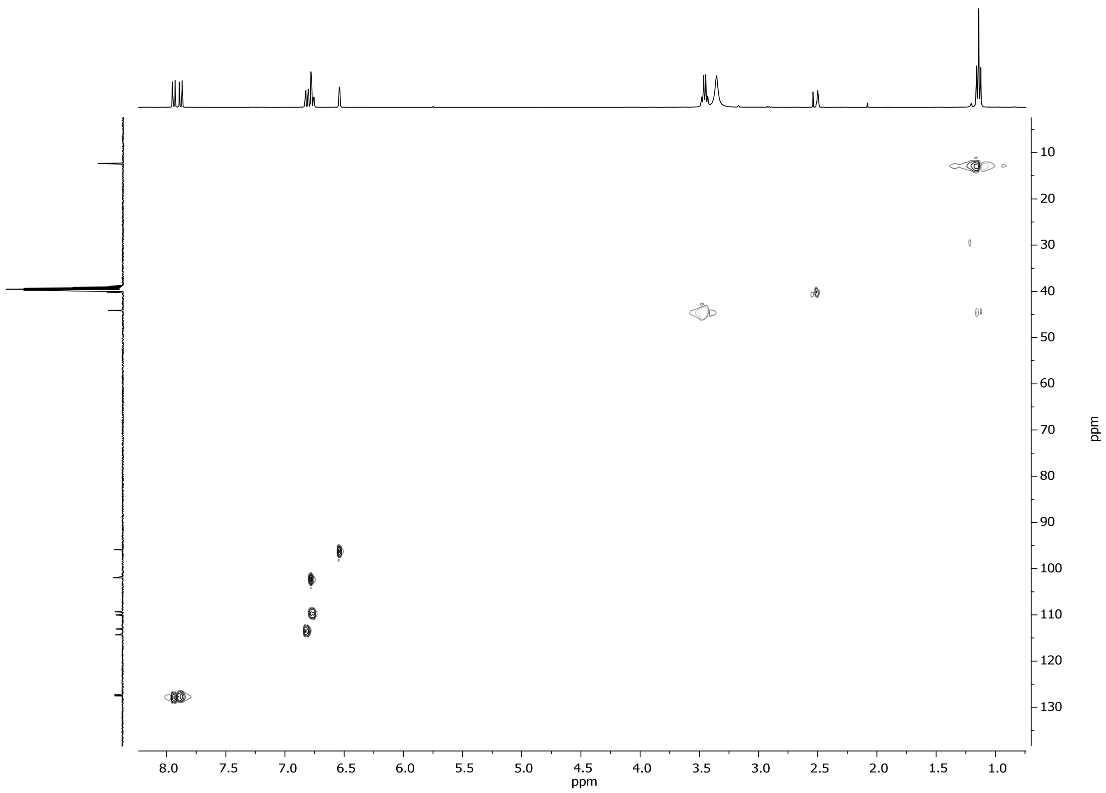

*Figure S3. HSQC spectrum of 3-(diethylamino)-6-hydroxy-9H-xanthen-9-one (**7**).*

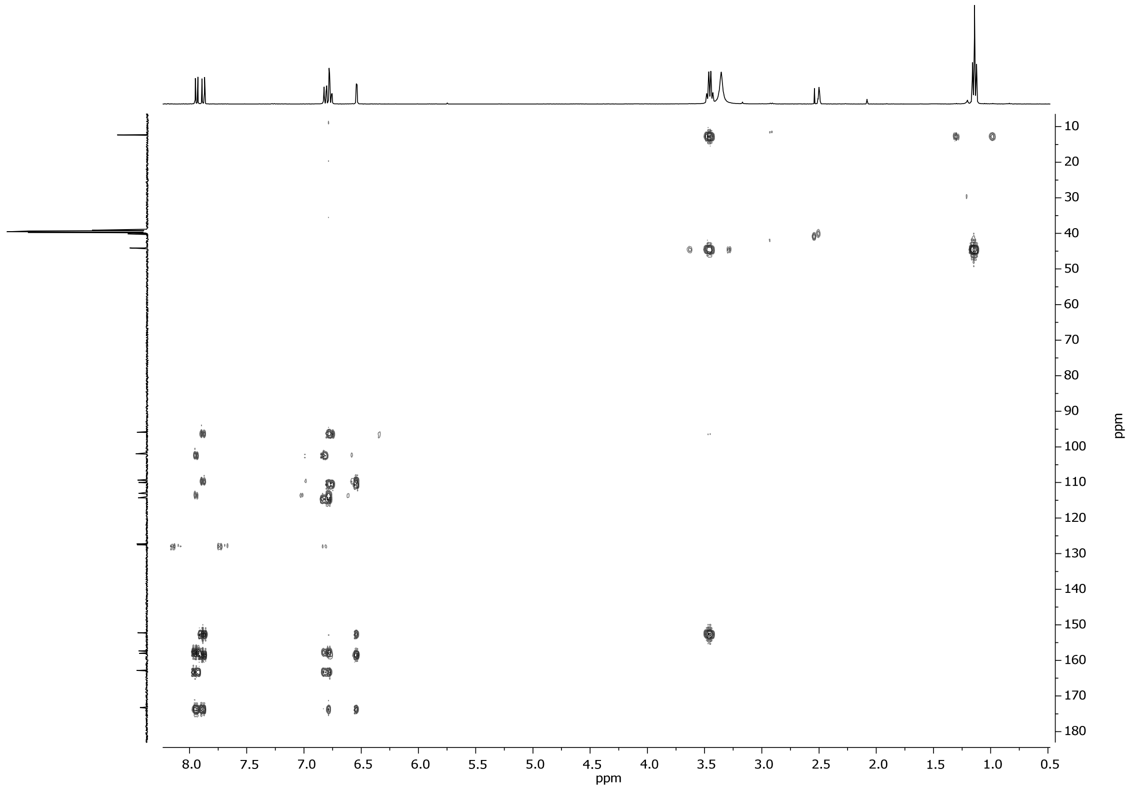

*Figure S4. HMBC spectrum of 3-(diethylamino)-6-hydroxy-9H-xanthen-9-one (**7**).*

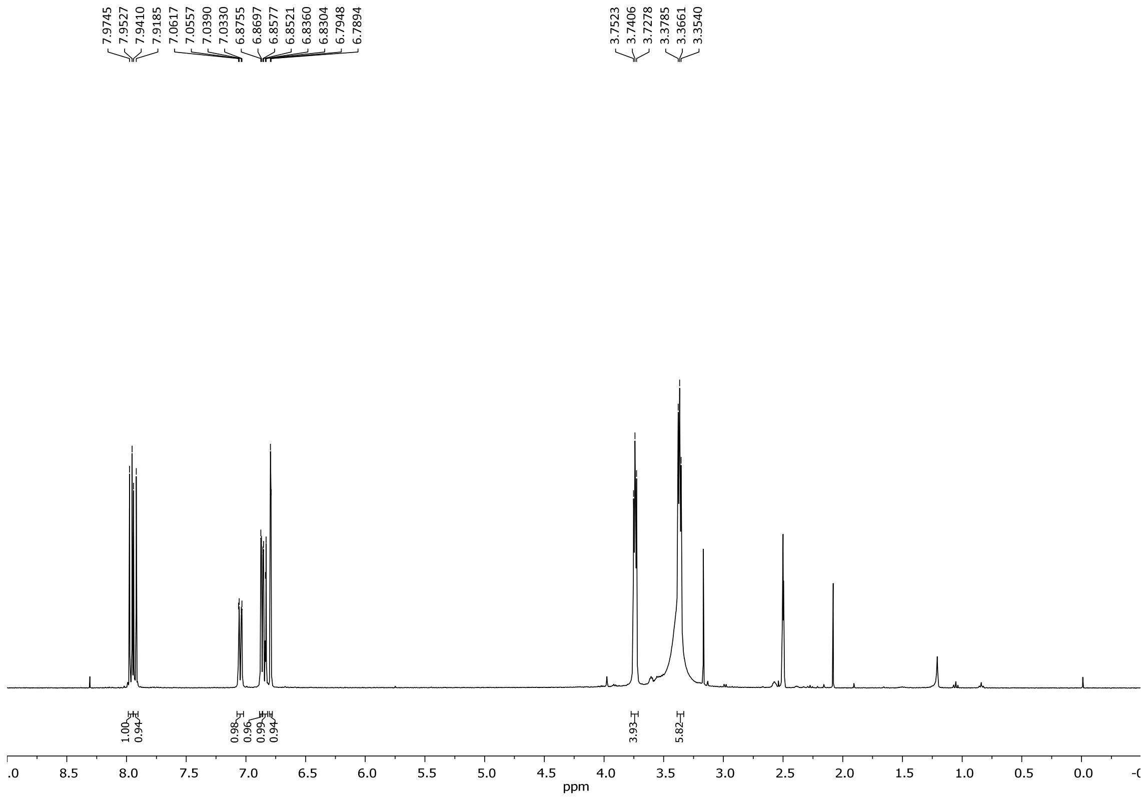

*Figure S5. ¹H NMR spectrum of 3-hydroxy-6-morpholino-9H-xanthen-9-one (**9**) (DMSO-d$_6$, 400 MHz).*

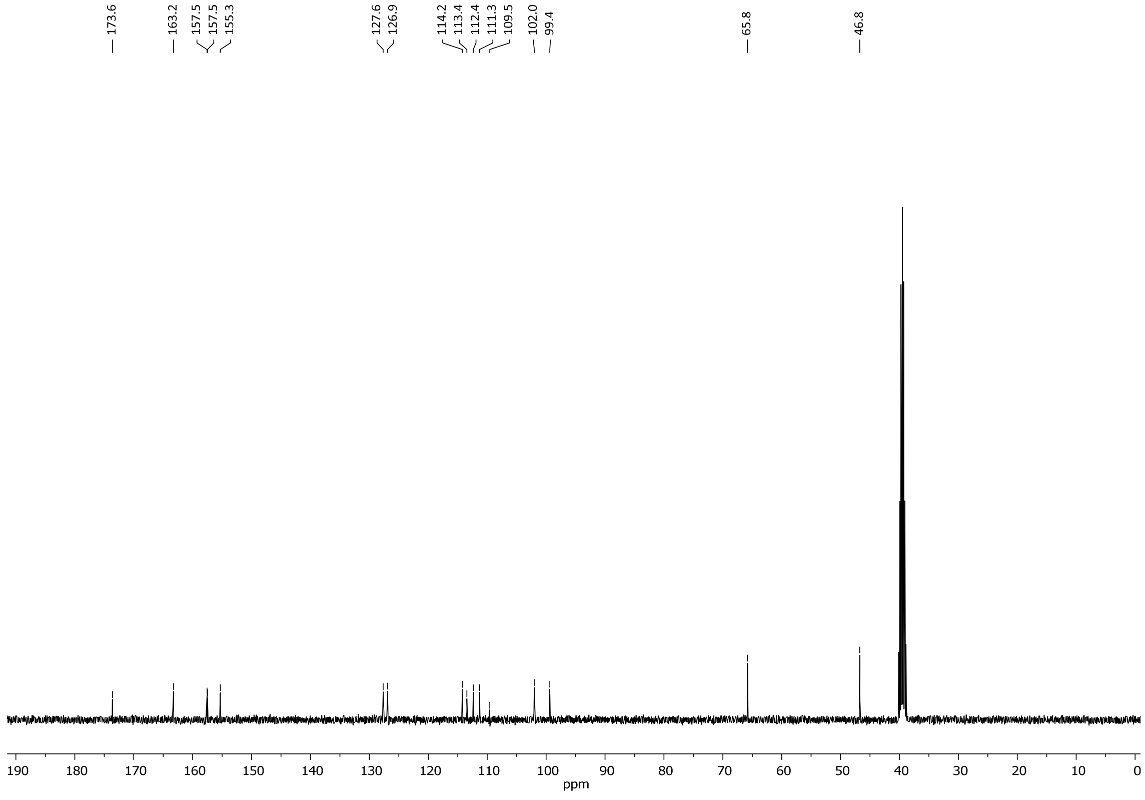

*Figure S6. ¹³C NMR spectrum of 3-hydroxy-6-morpholino-9H-xanthen-9-one (**9**) (DMSO-d$_6$, 101 MHz).*

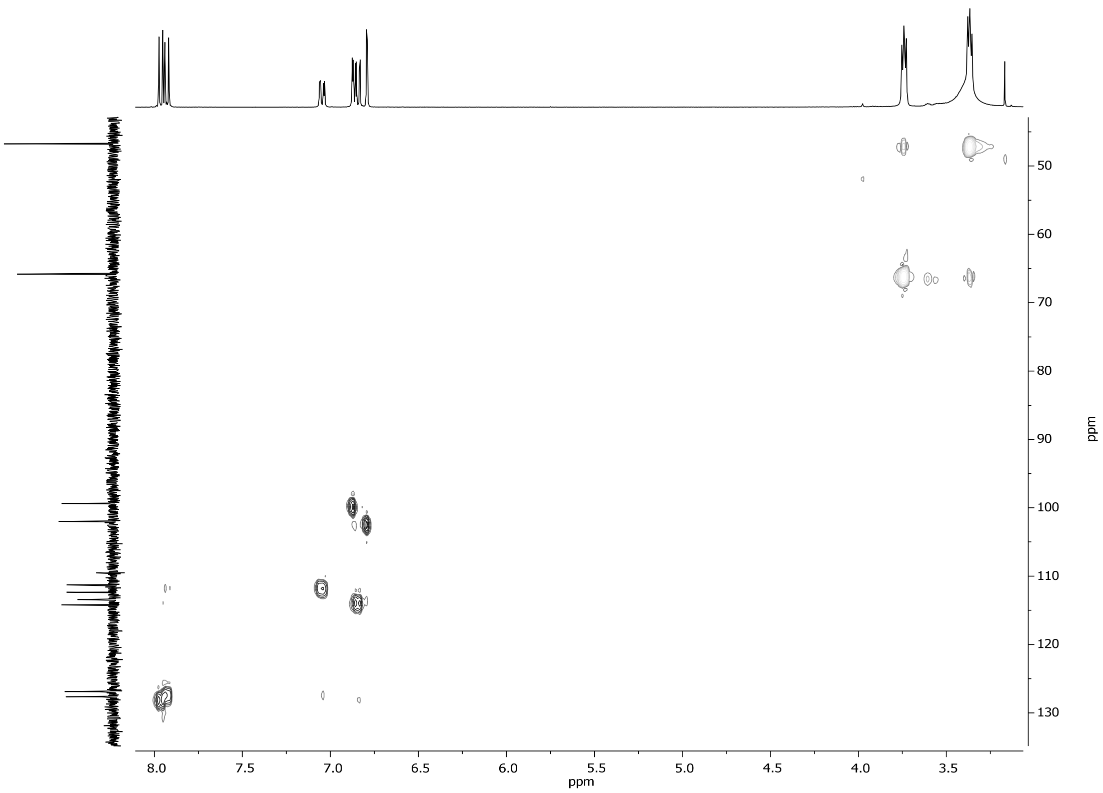

*Figure S7. HSQC spectrum of 3-hydroxy-6-morpholino-9H-xanthen-9-one (**9**).*

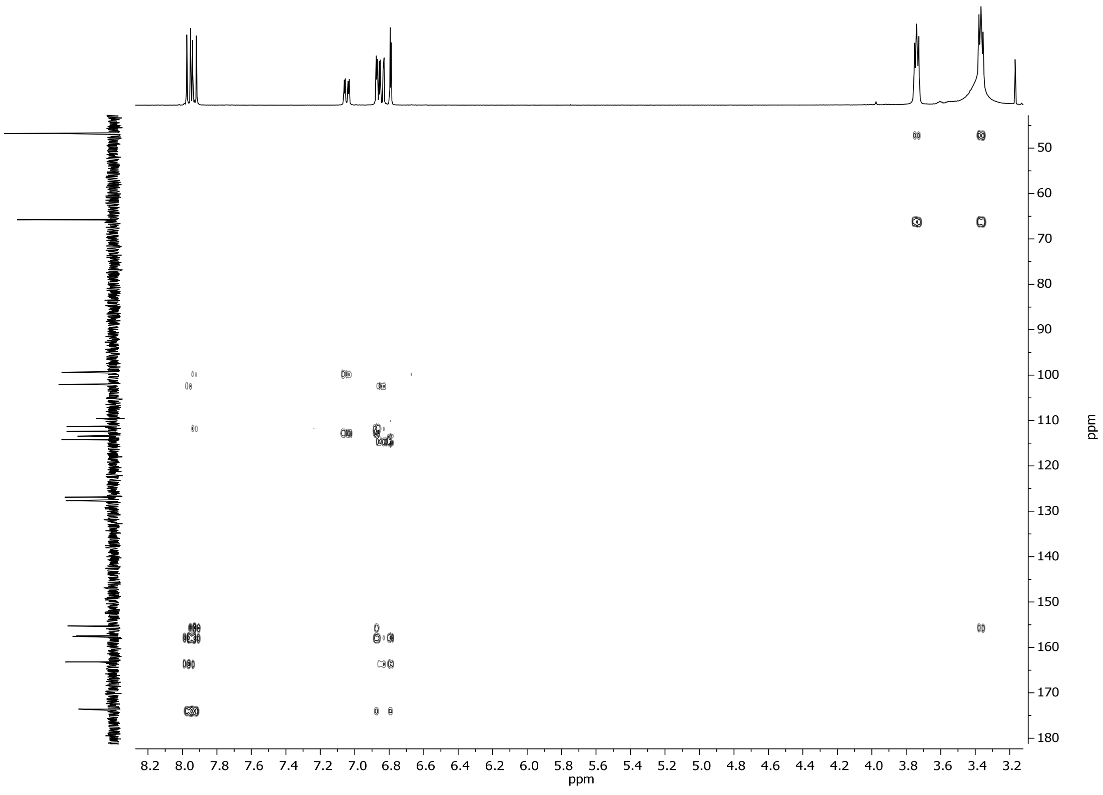

*Figure S8. HMBC spectrum of 3-hydroxy-6-morpholino-9H-xanthen-9-one (**9**).*

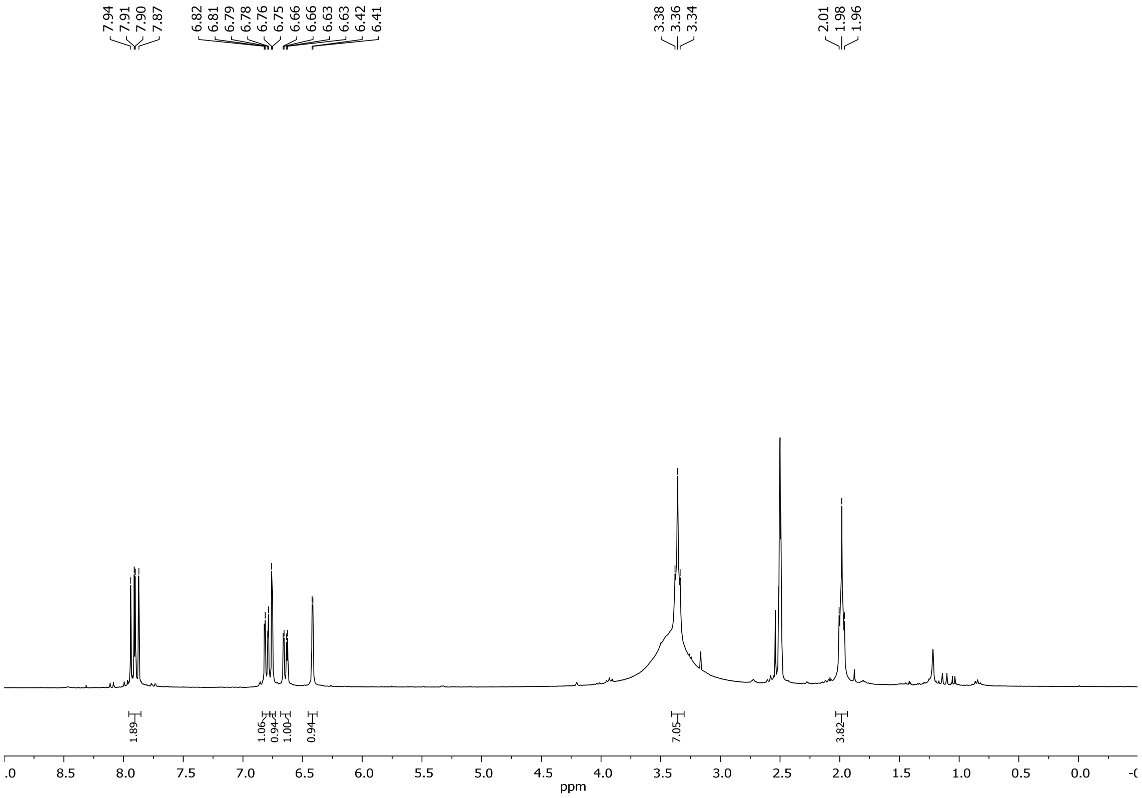

*Figure S9. ^{1}H NMR spectrum of 3-hydroxy-6-(pyrrolidin-1-yl)-9H-xanthen-9-one (**12**) (DMSO-d_6, 300 MHz).*

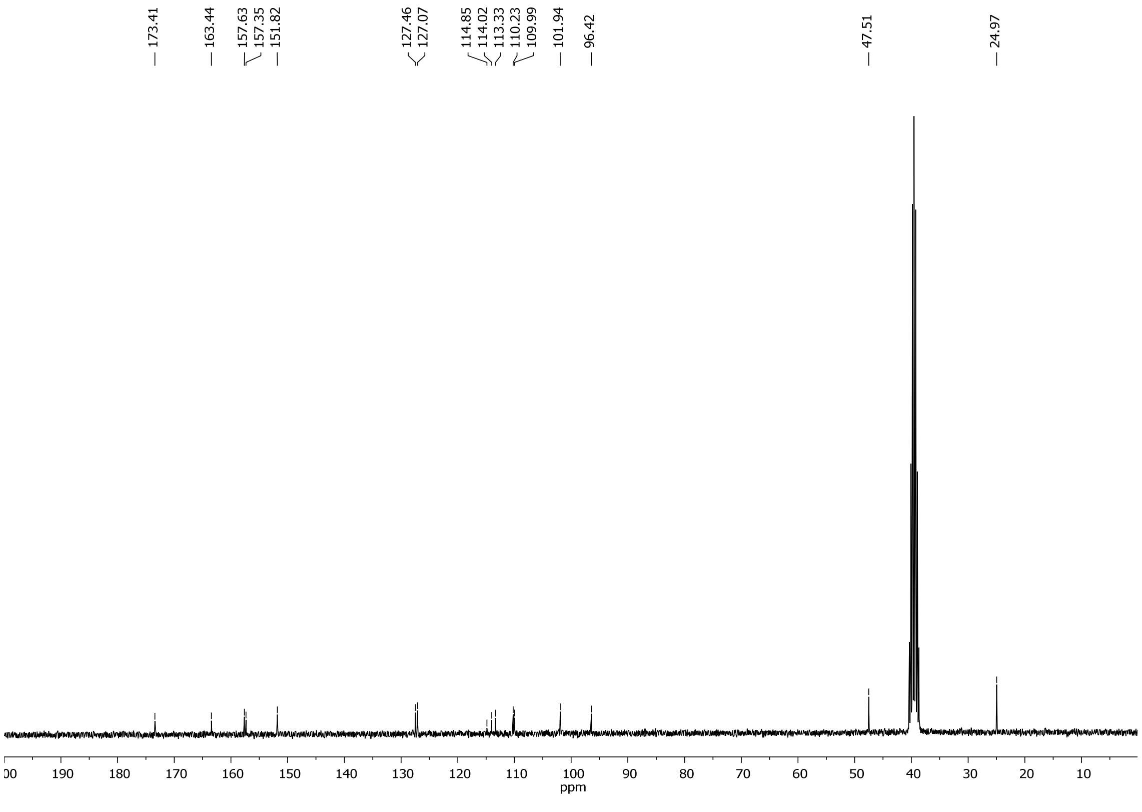

*Figure S10. ^{13}C NMR spectrum of 3-hydroxy-6-(pyrrolidin-1-yl)-9H-xanthen-9-one (**12**) (DMSO-d_6, 75 MHz).*

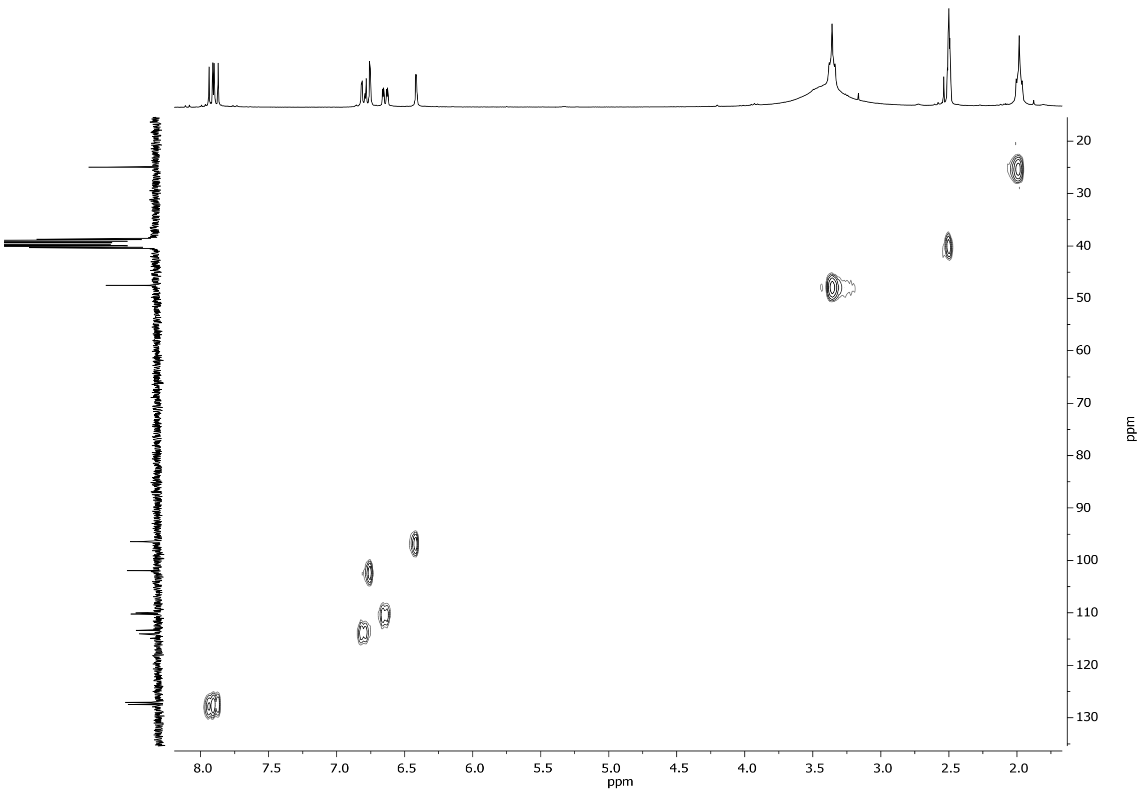

*Figure S11. HSQC spectrum of 3-hydroxy-6-(pyrrolidin-1-yl)-9H-xanthen-9-one (**12**).*

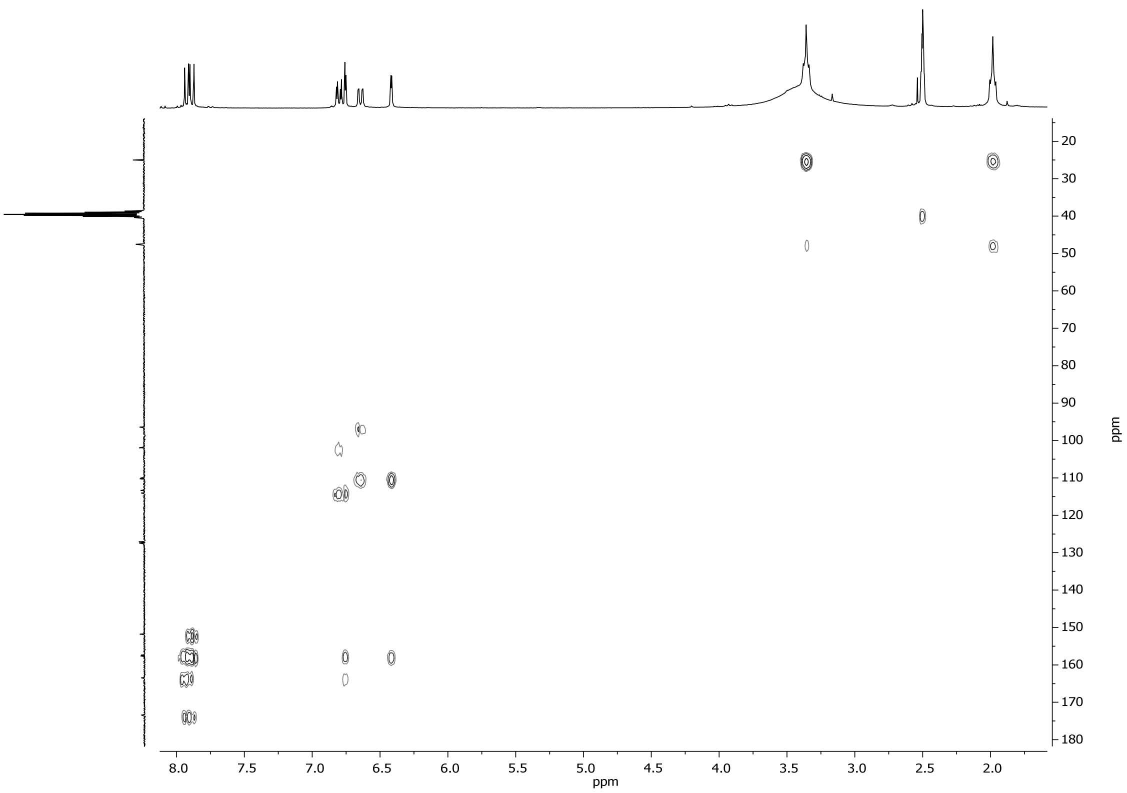

*Figure S12. HMBC spectrum of 3-hydroxy-6-(pyrrolidin-1-yl)-9H-xanthen-9-one (**12**).*

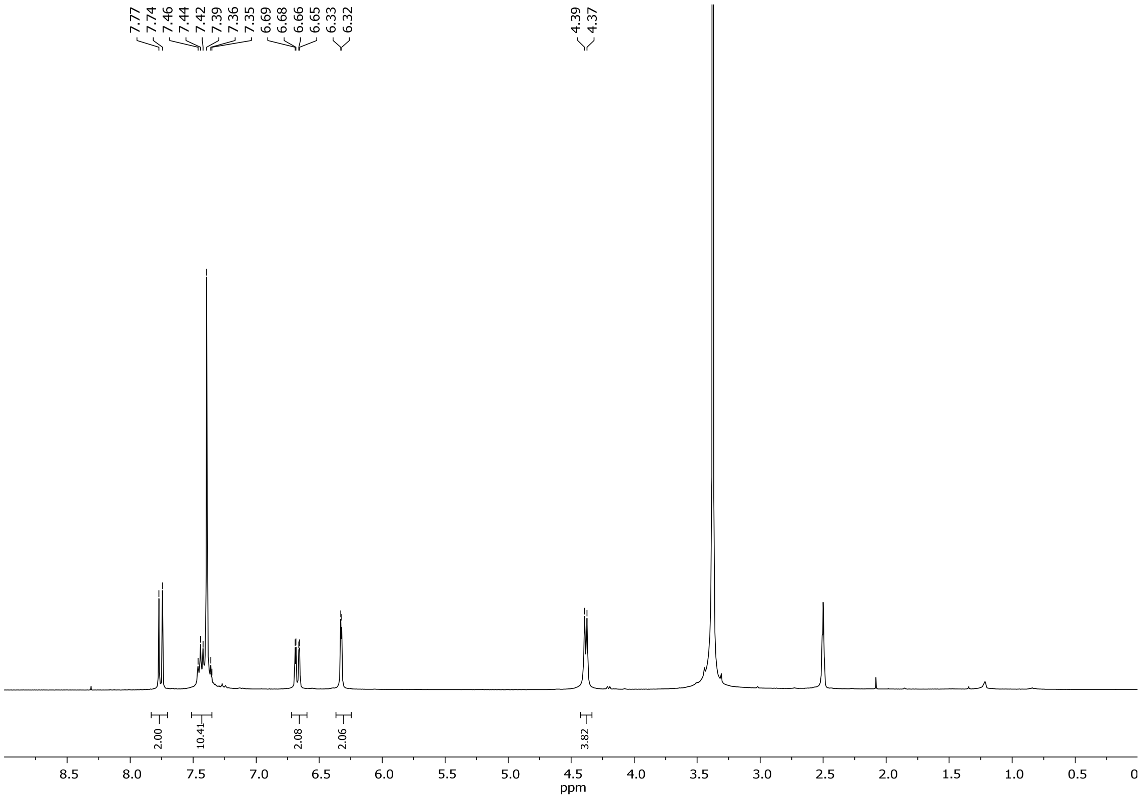

*Figure S13. ¹H NMR spectrum of 3,6-bis((4-chlorobenzyl)amino)-9H-xanthen-9-one (**13**) (DMSO-d$_6$, 300 MHz).*

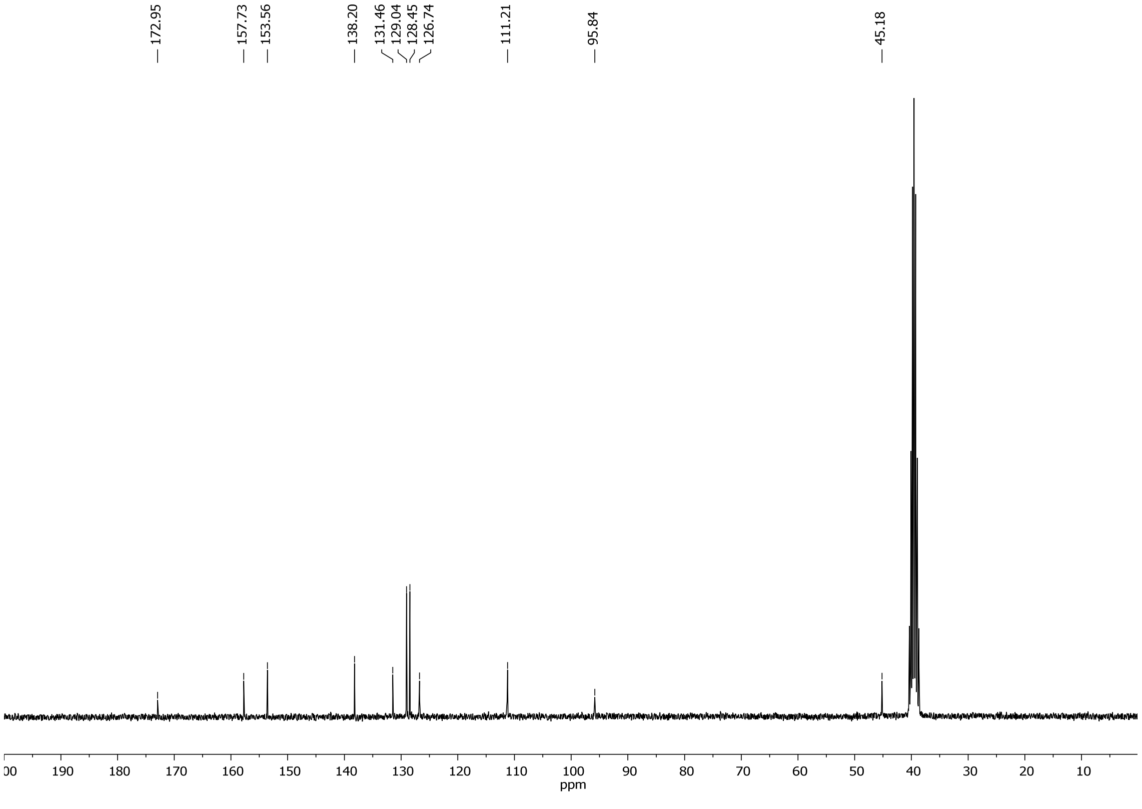

*Figure S14. ¹³C NMR spectrum of 3,6-bis((4-chlorobenzyl)amino)-9H-xanthen-9-one (**13**) (DMSO-d$_6$, 75 MHz).*

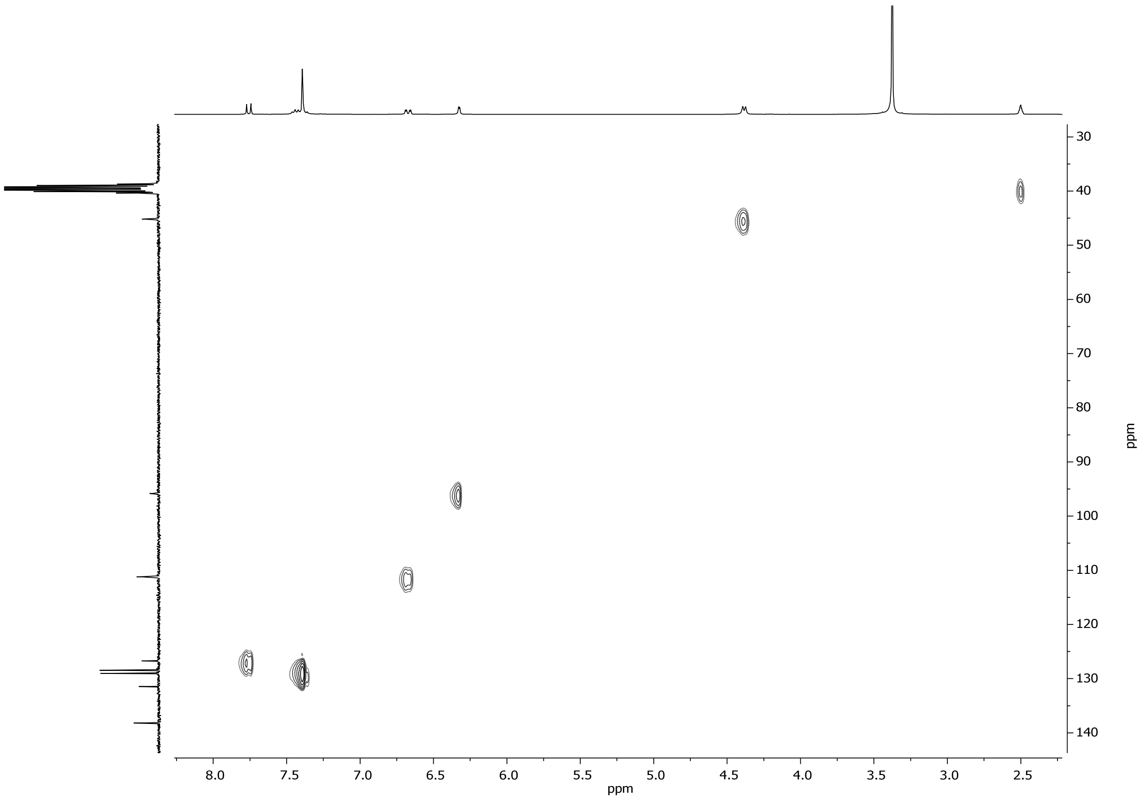

*Figure S15. HSQC spectrum of 3,6-bis((4-chlorobenzyl)amino)-9H-xanthen-9-one (**13**).*

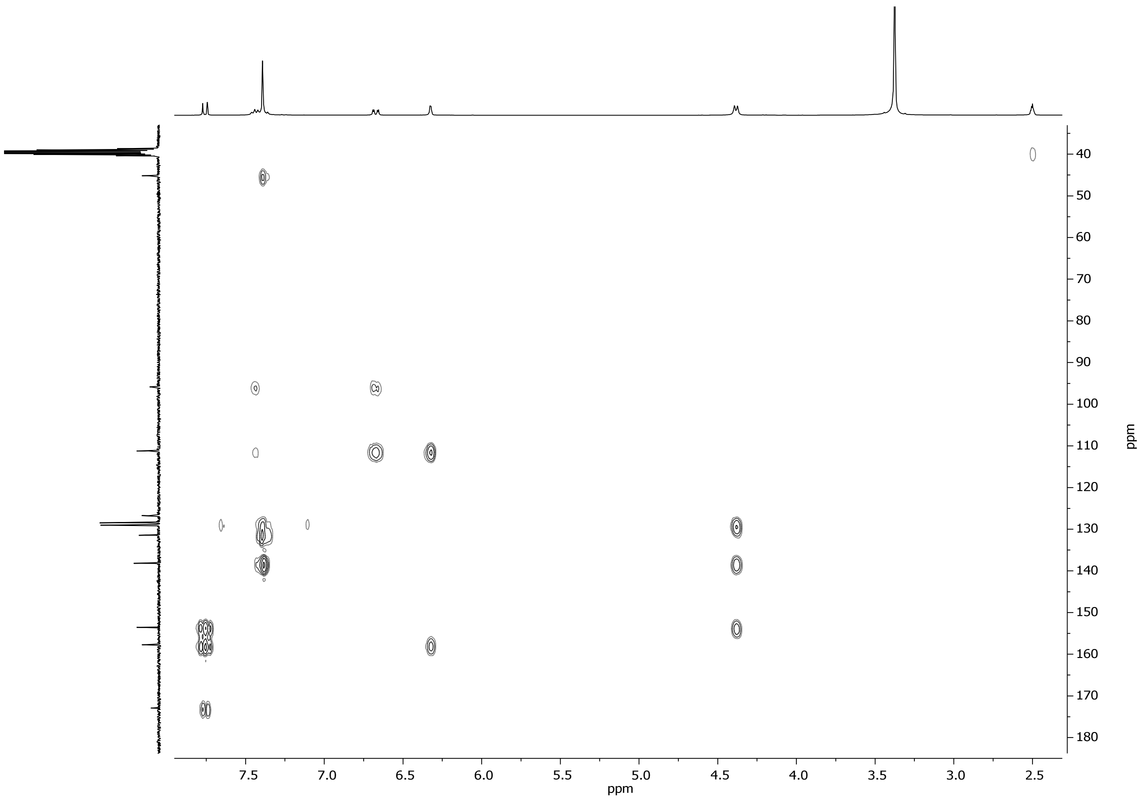

*Figure S16. HMBC spectrum of 3,6-bis((4-chlorobenzyl)amino)-9H-xanthen-9-one (**13**).*

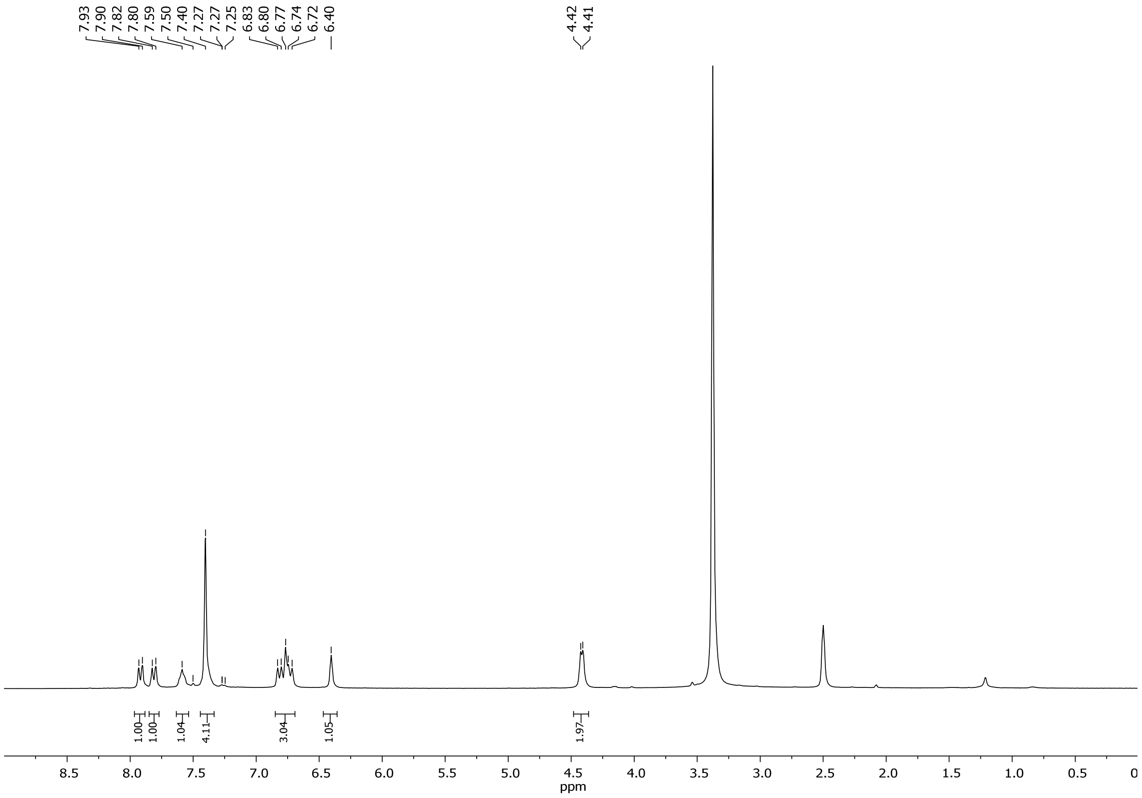

*Figure S17. ^{1}H NMR spectrum of of 3-((4-chlorobenzyl)amino)-6-hydroxy-9H-xanthen-9-one (**14**) (DMSO-d_6, 300 MHz).*

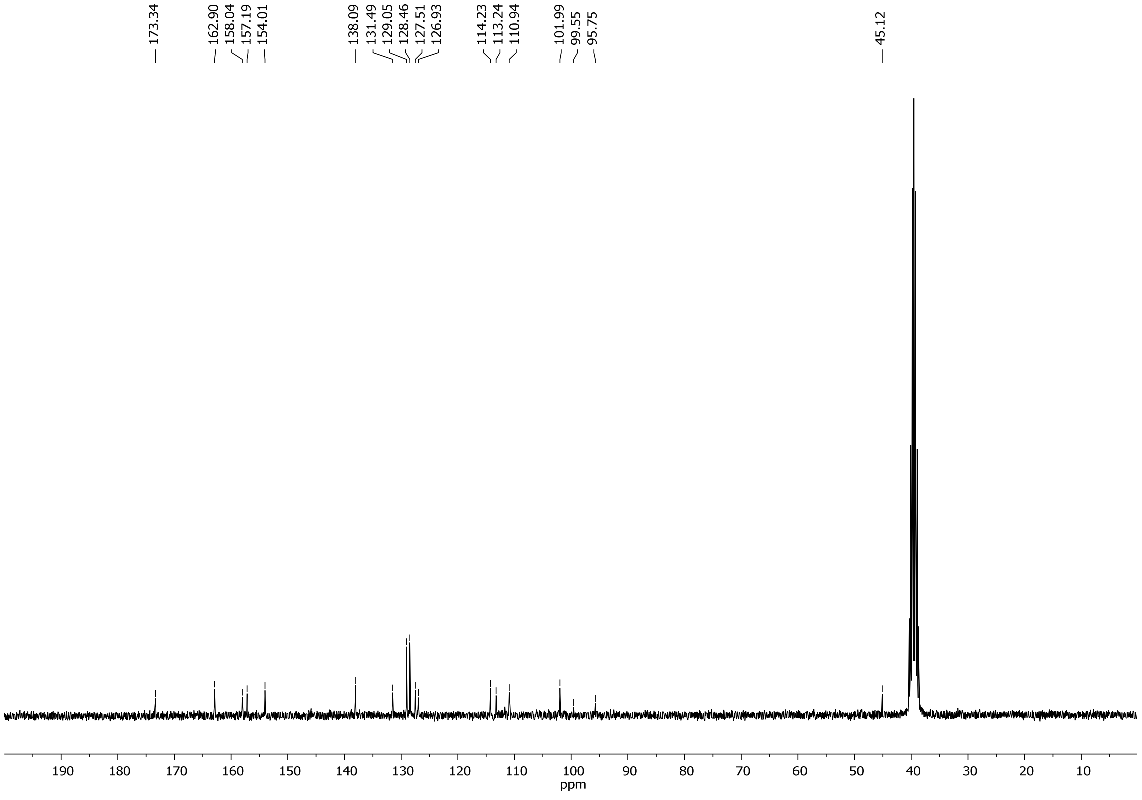

*Figure S18. ^{13}C NMR spectrum of of 3-((4-chlorobenzyl)amino)-6-hydroxy-9H-xanthen-9-one (**14**) (DMSO-d_6, 75 MHz).*

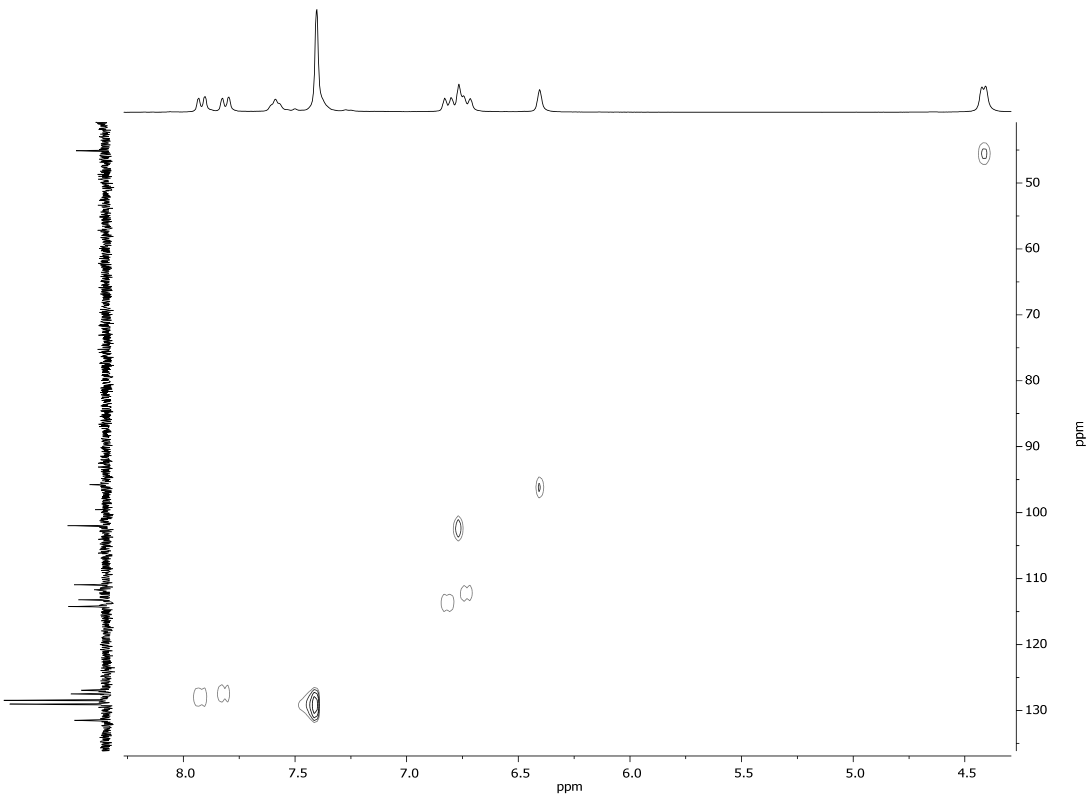

*Figure S 19. HMBC spectrum of 3-((4-chlorobenzyl)amino)-6-hydroxy-9H-xanthen-9-one (**14**).*

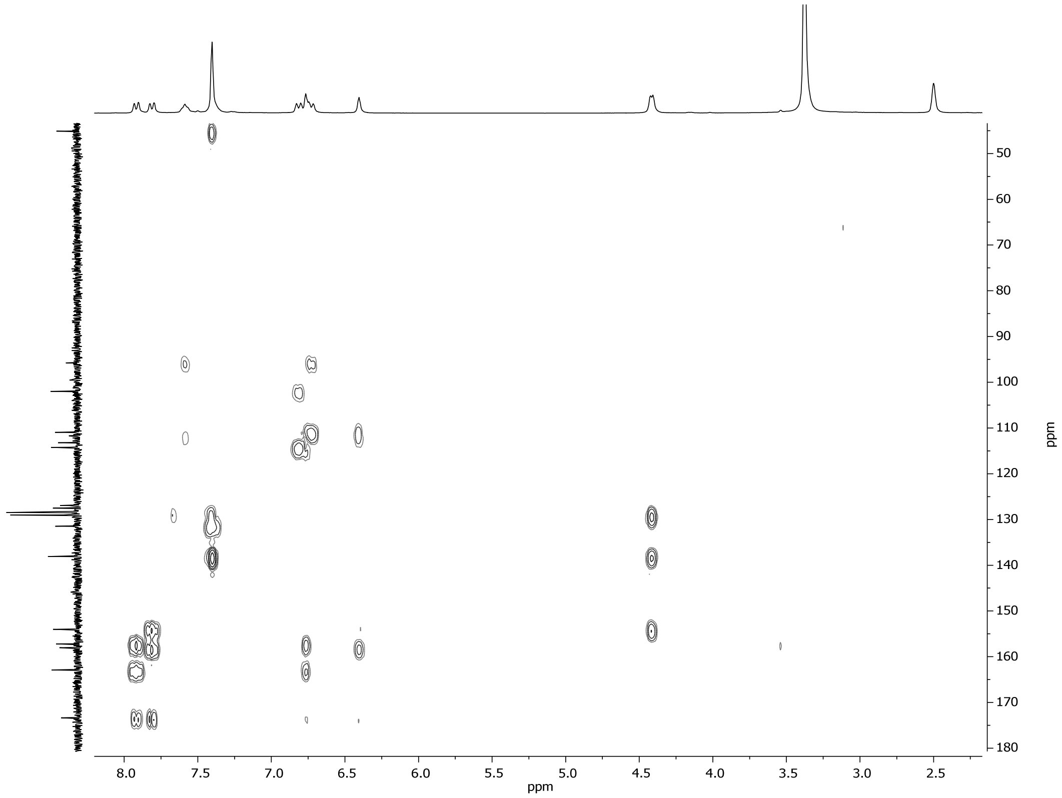

*Figure S20. HMBC spectrum of 3-((4-chlorobenzyl)amino)-6-hydroxy-9H-xanthen-9-one (**14**).*

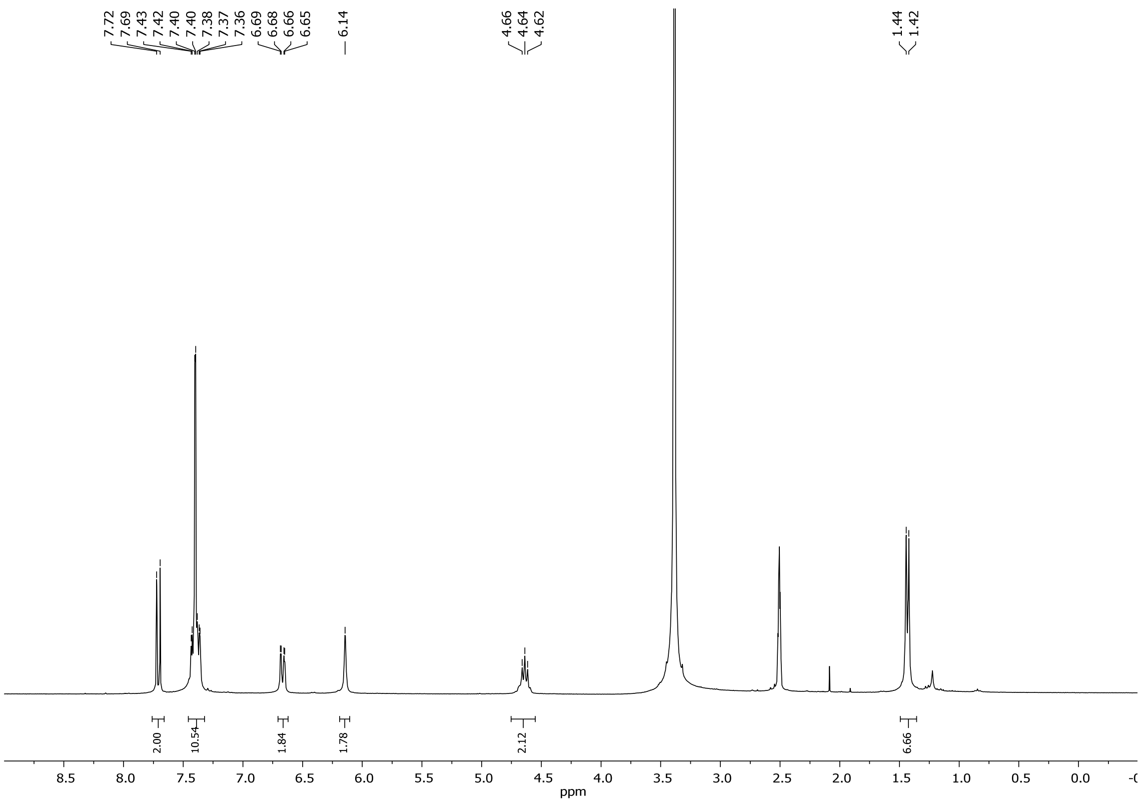

*Figure S21. ^{1}H NMR spectrum of 3,6-bis(((R)-1-(4-chlorophenyl)ethyl)amino)-9H-xanthen-9-one (**15**) (DMSO-d6, 300 MHz).*

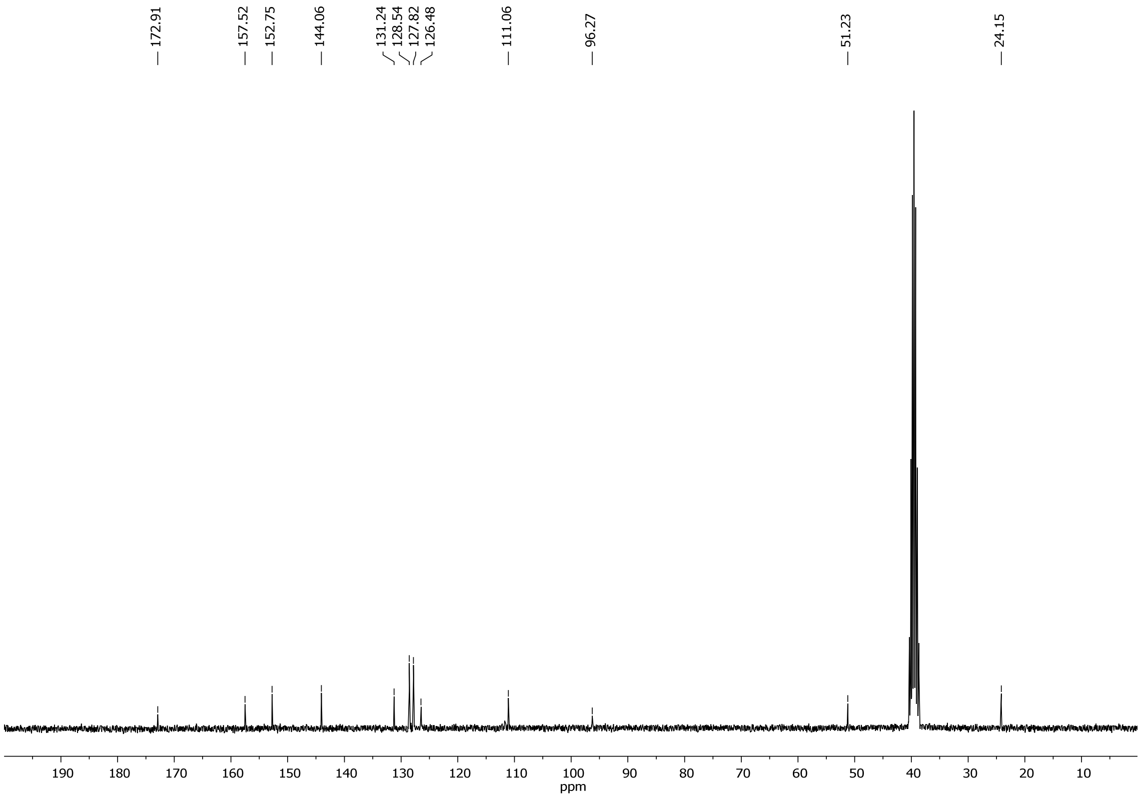

*Figure S22. ^{13}C NMR spectrum of 3,6-bis(((R)-1-(4-chlorophenyl)ethyl)amino)-9H-xanthen-9-one (**15**) (DMSO-d6, 75 MHz).*

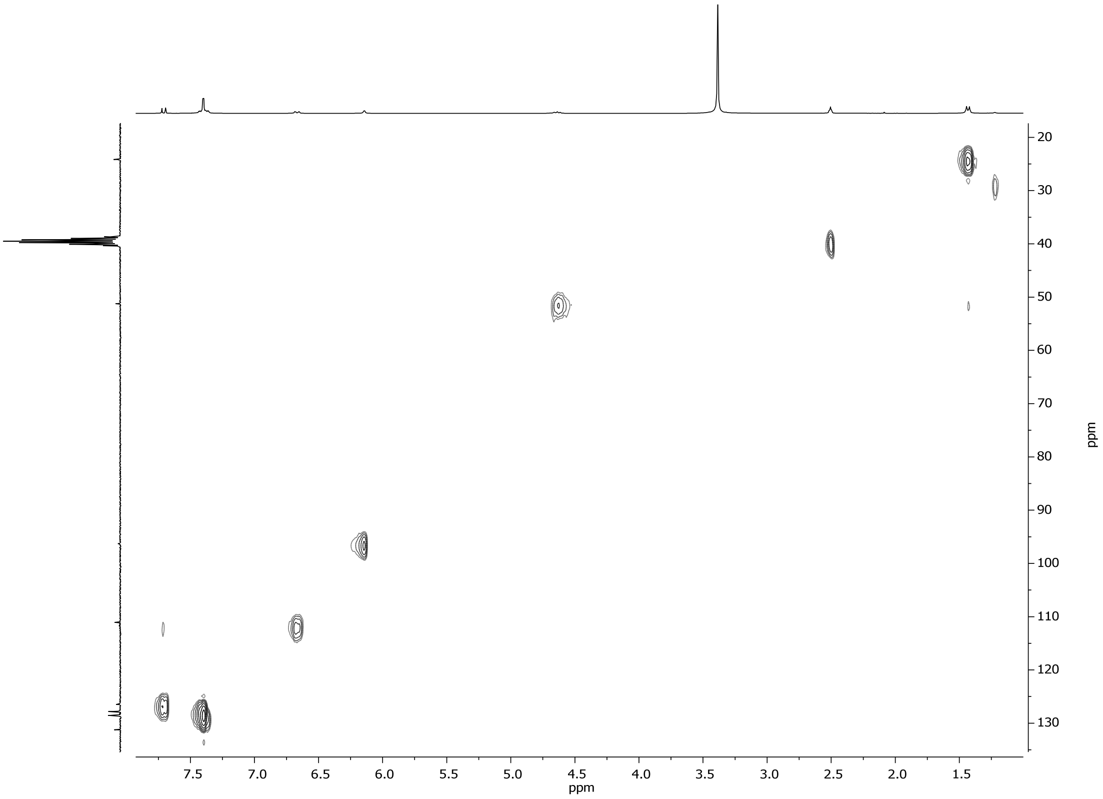

*Figure S23. HSQC spectrum of 3,6-bis(((R)-1-(4-chlorophenyl)ethyl)amino)-9H-xanthen-9-one (**15**).*

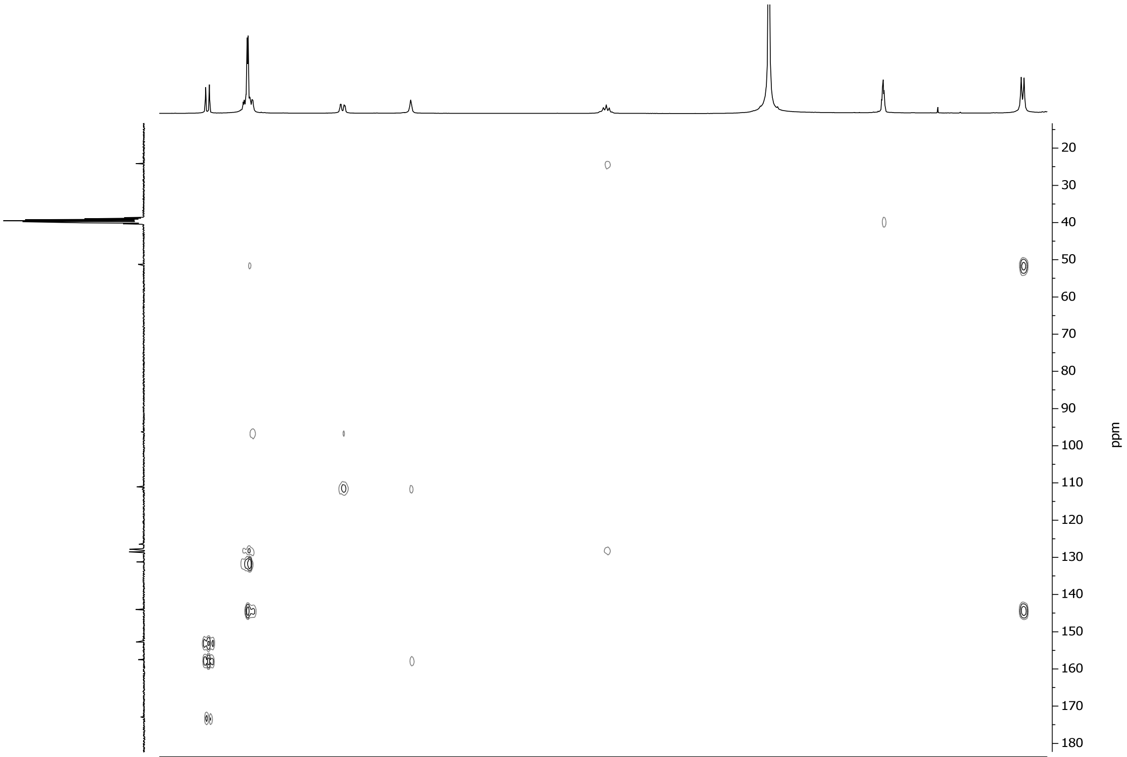

*Figure S24. HMBC spectrum of 3,6-bis(((R)-1-(4-chlorophenyl)ethyl)amino)-9H-xanthen-9-one (**15**).*

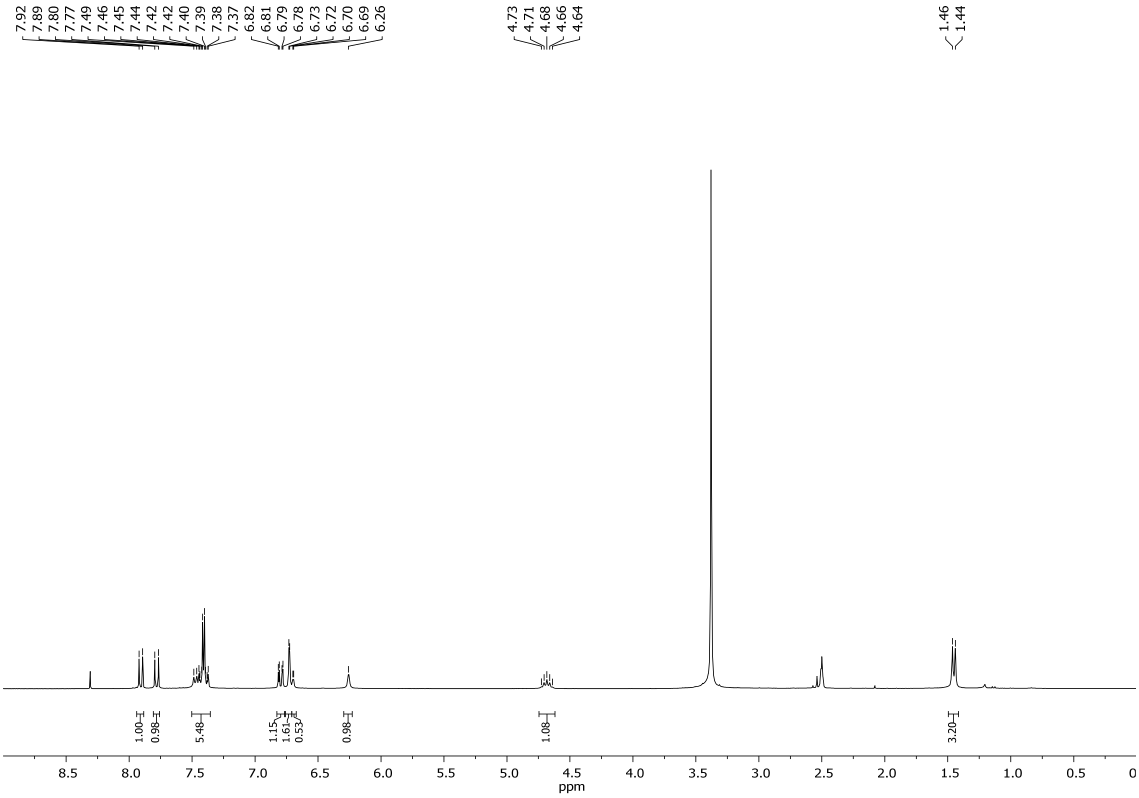

*Figure S25. ^{1}H NMR spectrum of (R)-3-((1-(4-chlorophenyl)ethyl)amino)-6-hydroxy-9H-xanthen-9-one (**16**) (DMSO-d_6, 300 MHz).*

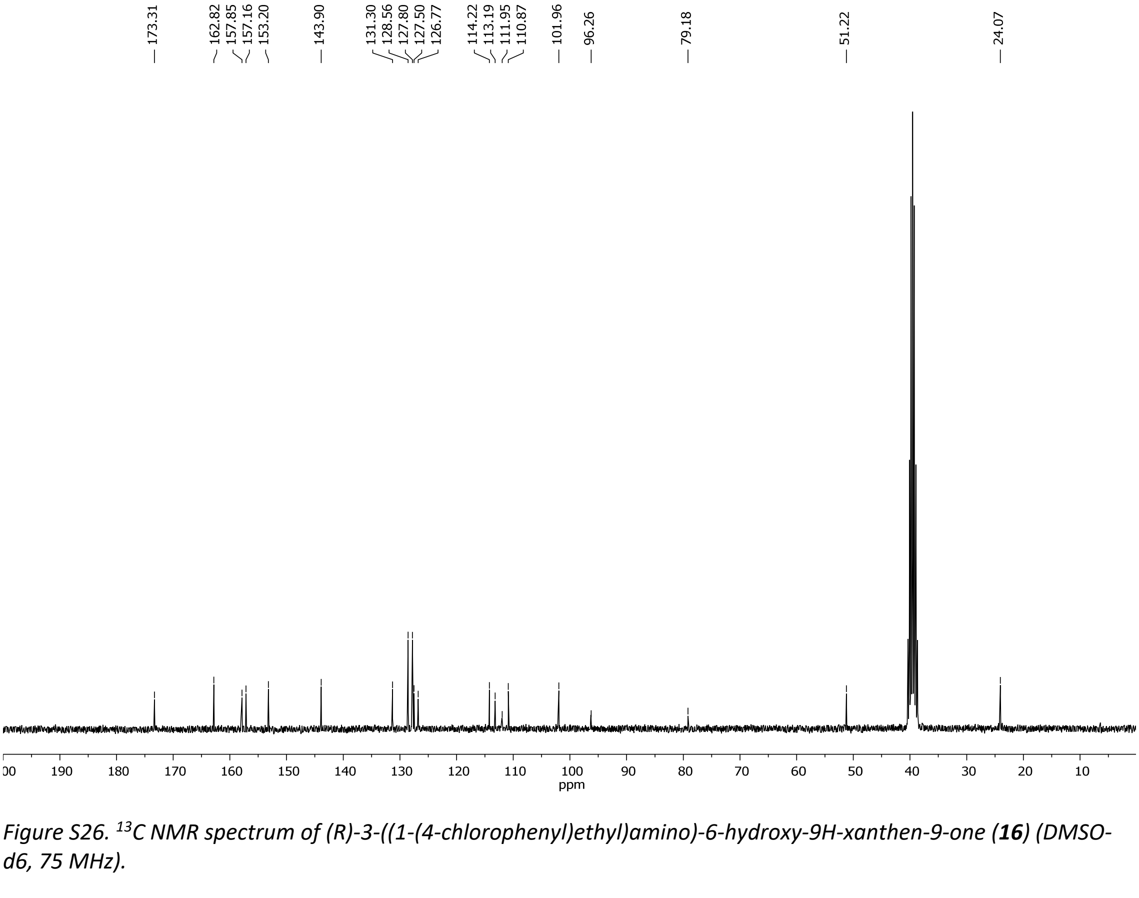

*Figure S26. ^{13}C NMR spectrum of (R)-3-((1-(4-chlorophenyl)ethyl)amino)-6-hydroxy-9H-xanthen-9-one (**16**) (DMSO-d6, 75 MHz).*

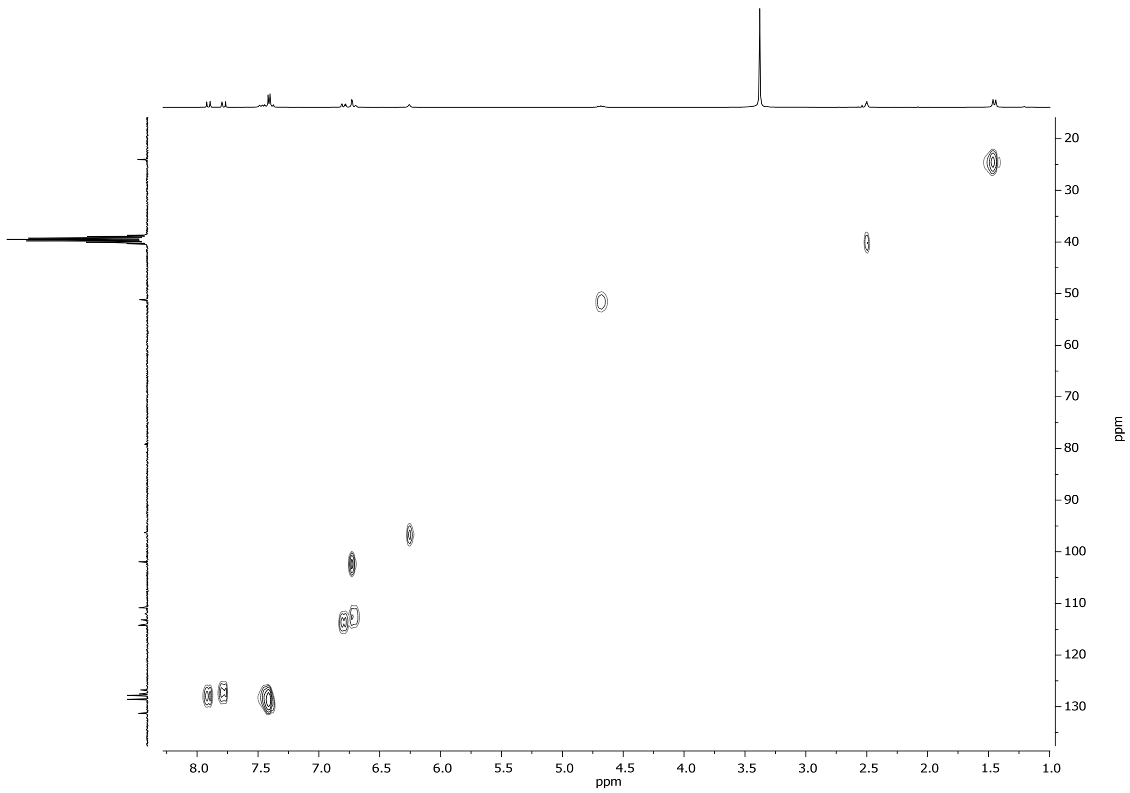

*Figure S27 HSQC spectrum of (R)-3-((1-(4-chlorophenyl)ethyl)amino)-6-hydroxy-9H-xanthen-9-one (**16**).*

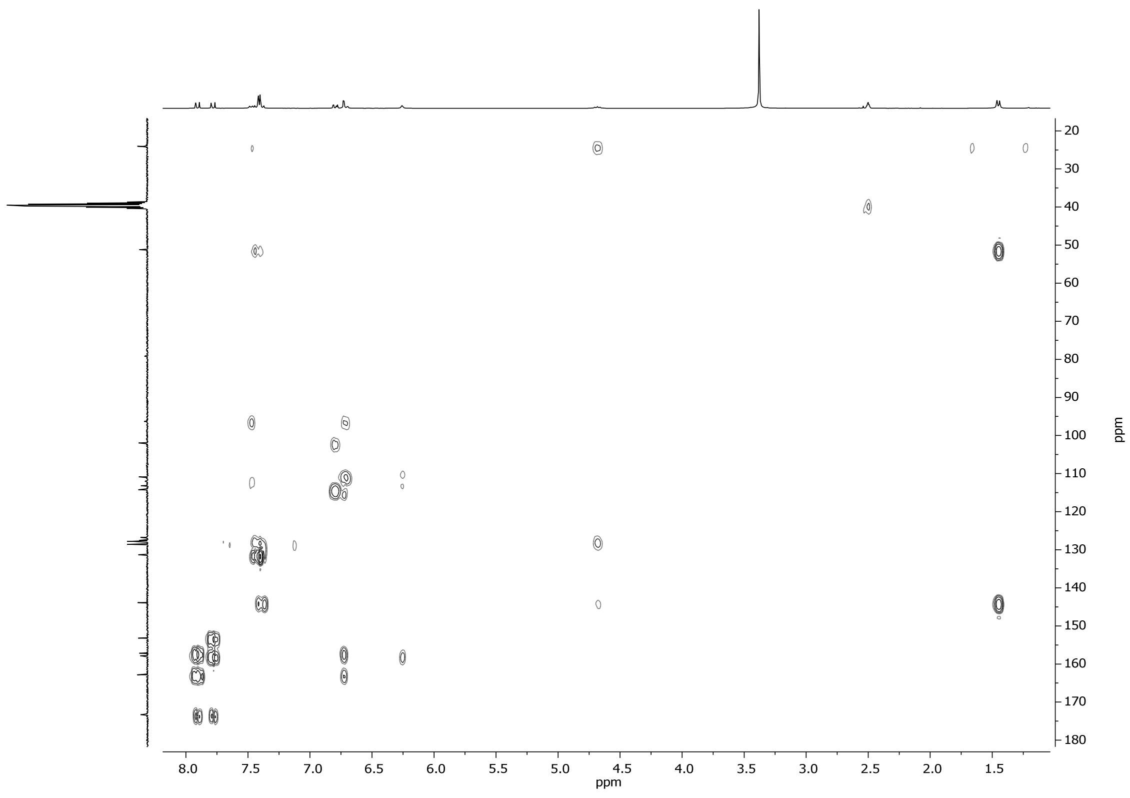

*Figure S28. HMBC spectrum of (R)-3-((1-(4-chlorophenyl)ethyl)amino)-6-hydroxy-9H-xanthen-9-one (**16**).*

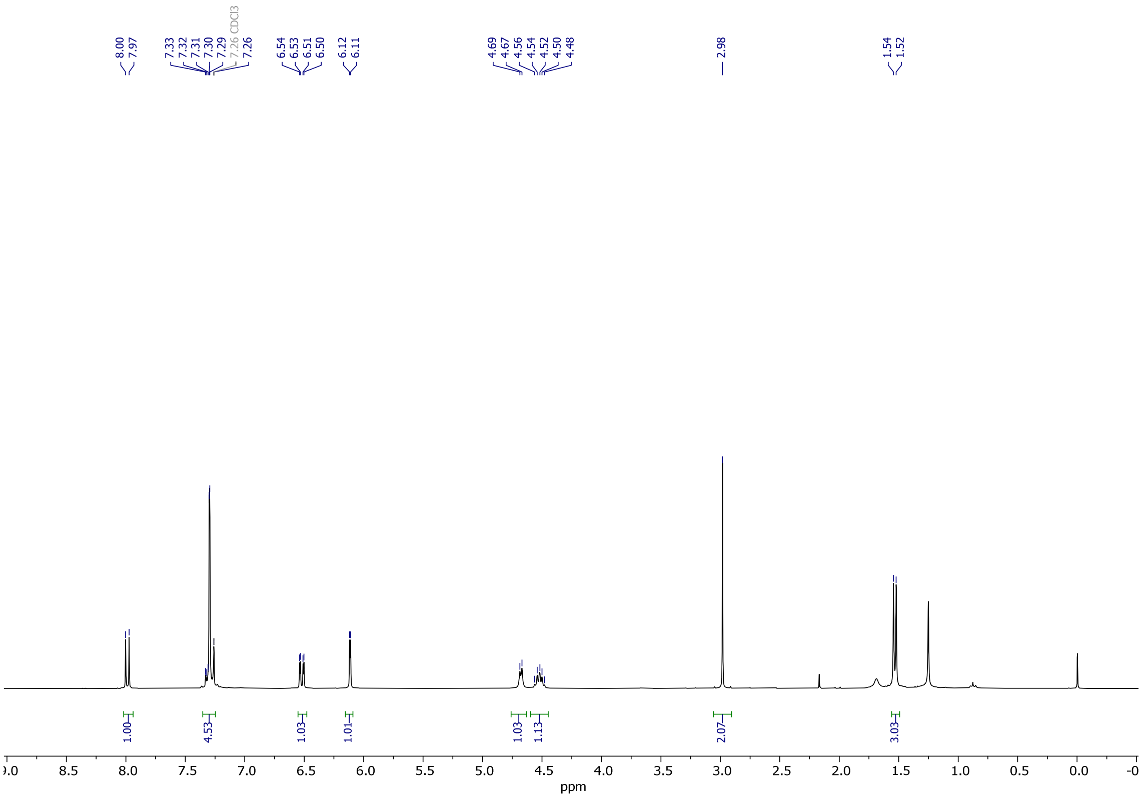

*Figure S29. ^{1}H NMR spectrum of 3,6-bis(((S)-1-(4-chlorophenyl)ethyl)amino)-9H-xanthen-9-one (**17**) (DMSO-d_6, 300 MHz).*

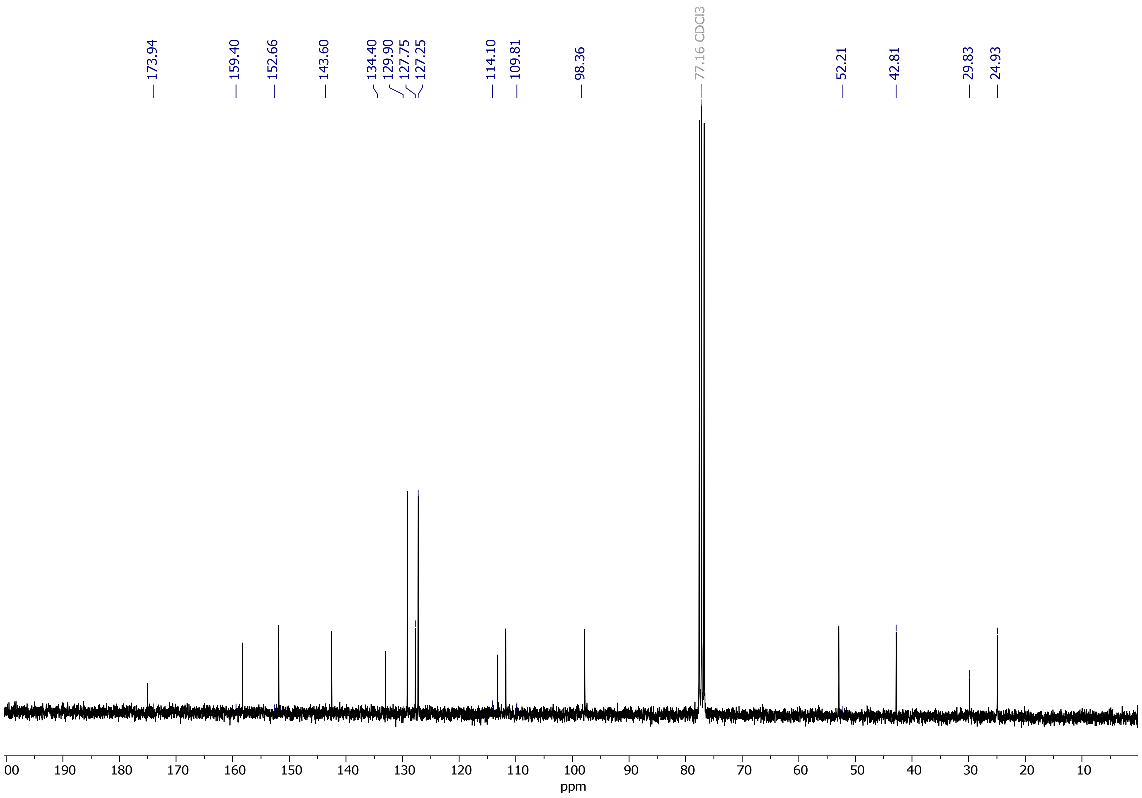

*Figure S30. ^{13}C NMR spectrum of 3,6-bis(((S)-1-(4-chlorophenyl)ethyl)amino)-9H-xanthen-9-one (**17**) (DMSO-d_6, 75 MHz).*

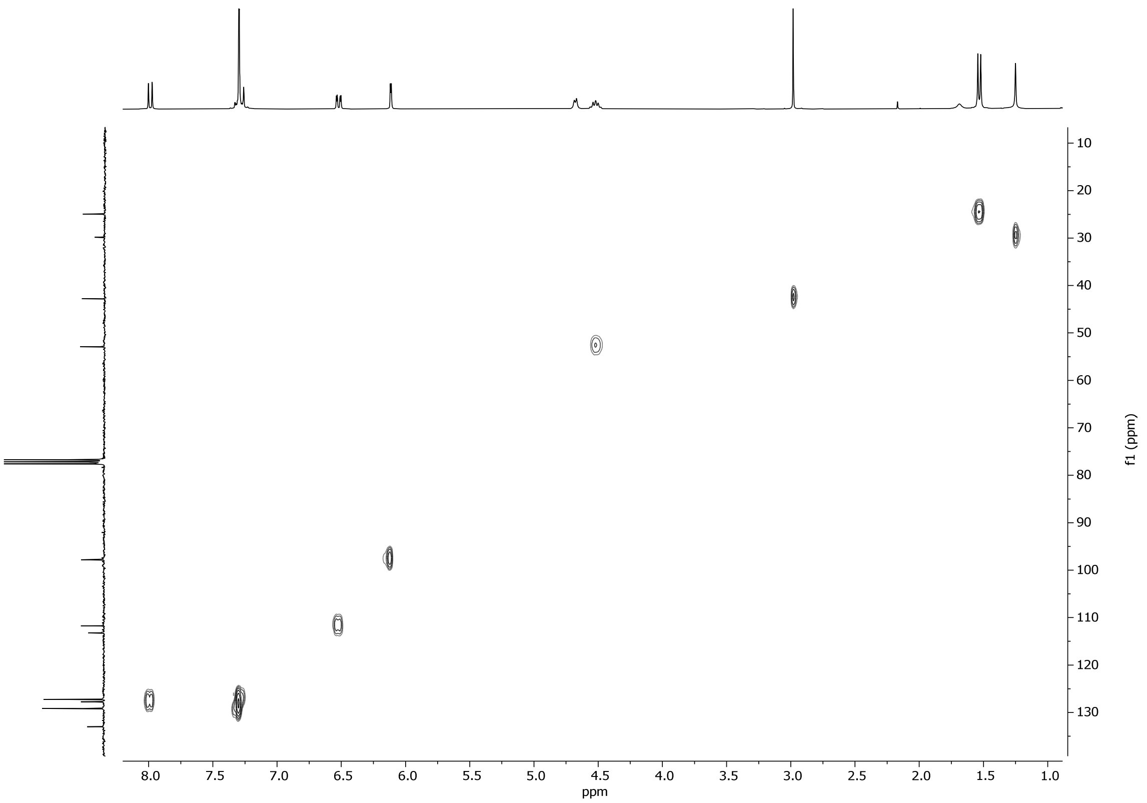

*Figure S31. HSQC spectrum of 3,6-bis(((S)-1-(4-chlorophenyl)ethyl)amino)-9H-xanthen-9-one (**17**).*

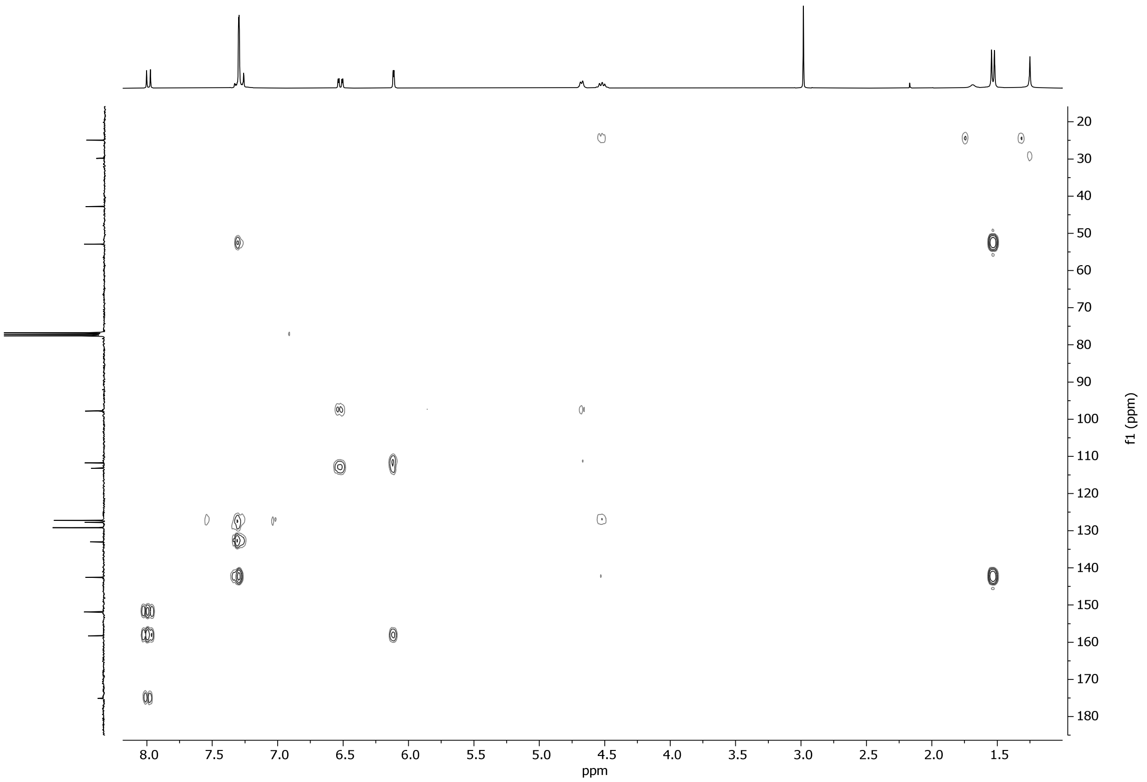

*Figure S32. HMBC spectrum of 3,6-bis((((S)-1-(4-chlorophenyl)ethyl)amino)-9H-xanthen-9-one (**17**).*

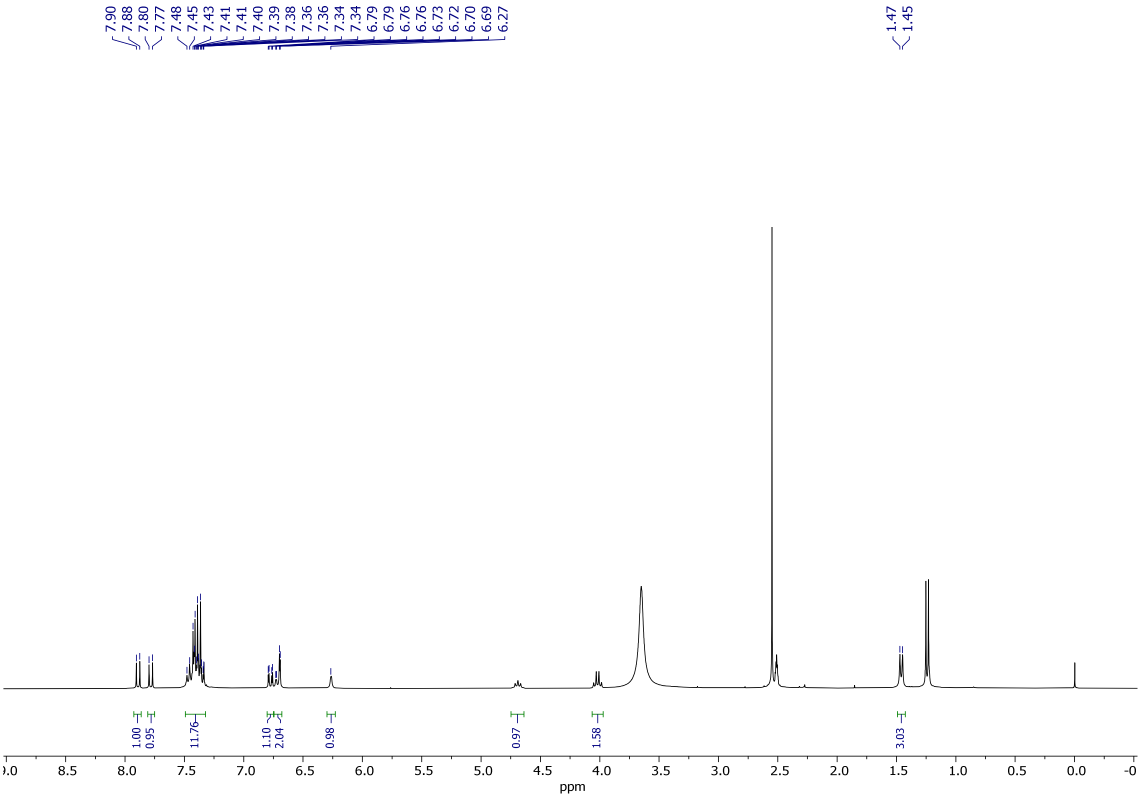

*Figure S33. ¹H NMR spectrum of (S)-3-((1-(4-chlorophenyl)ethyl)amino)-6-hydroxy-9H-xanthen-9-one (**18**) (DMSO-d6, 300 MHz).*

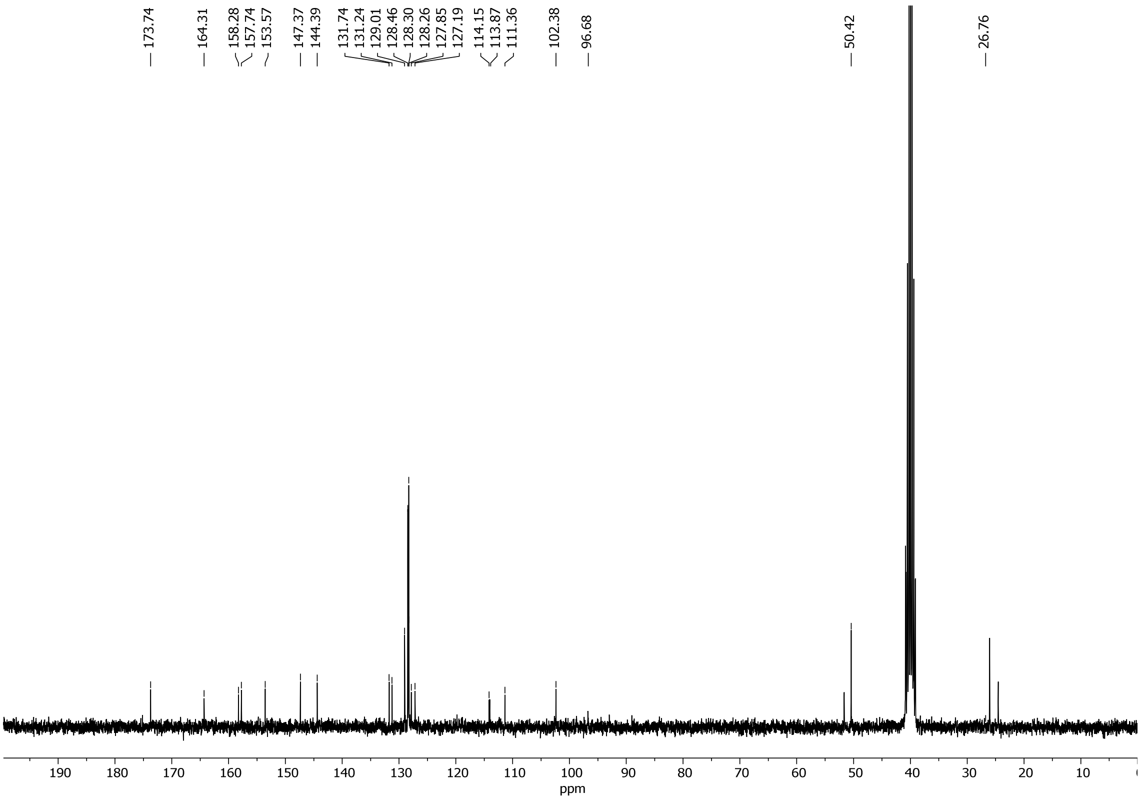

*Figure S34. ¹³C NMR spectrum of (S)-3-((1-(4-chlorophenyl)ethyl)amino)-6-hydroxy-9H-xanthen-9-one (**18**) (DMSO-d6, 75 MHz).*

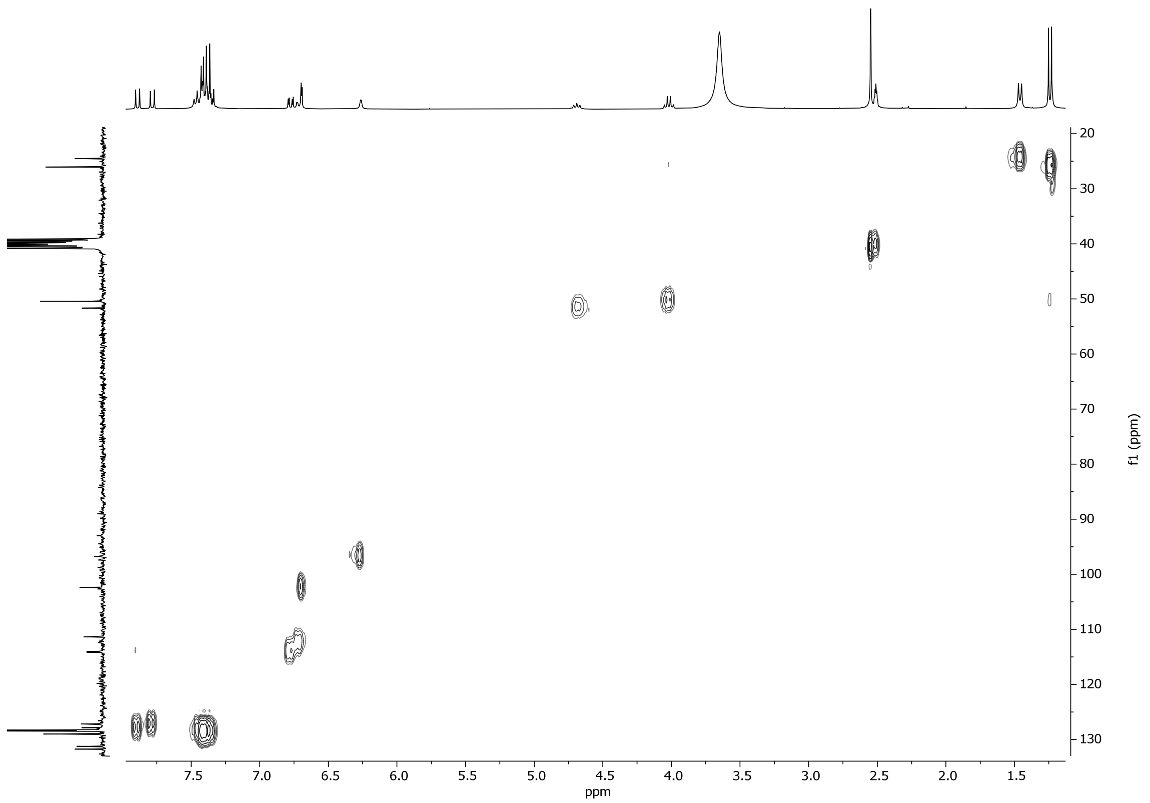

*Figure S35. HSQC spectrum of (S)-3-((1-(4-chlorophenyl)ethyl)amino)-6-hydroxy-9H-xanthen-9-one (**18**).*

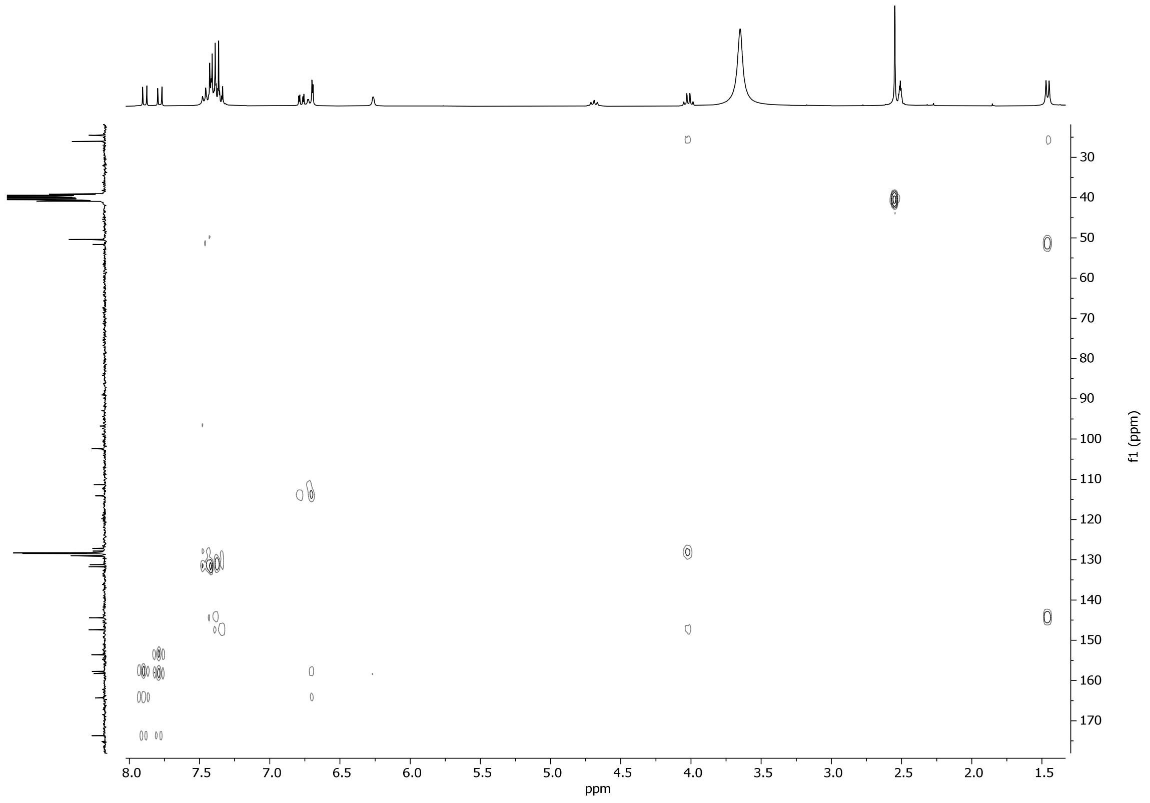

*Figure S36. HMBC spectrum of (S)-3-((1-(4-chlorophenyl)ethyl)amino)-6-hydroxy-9H-xanthen-9-one (**18**).*

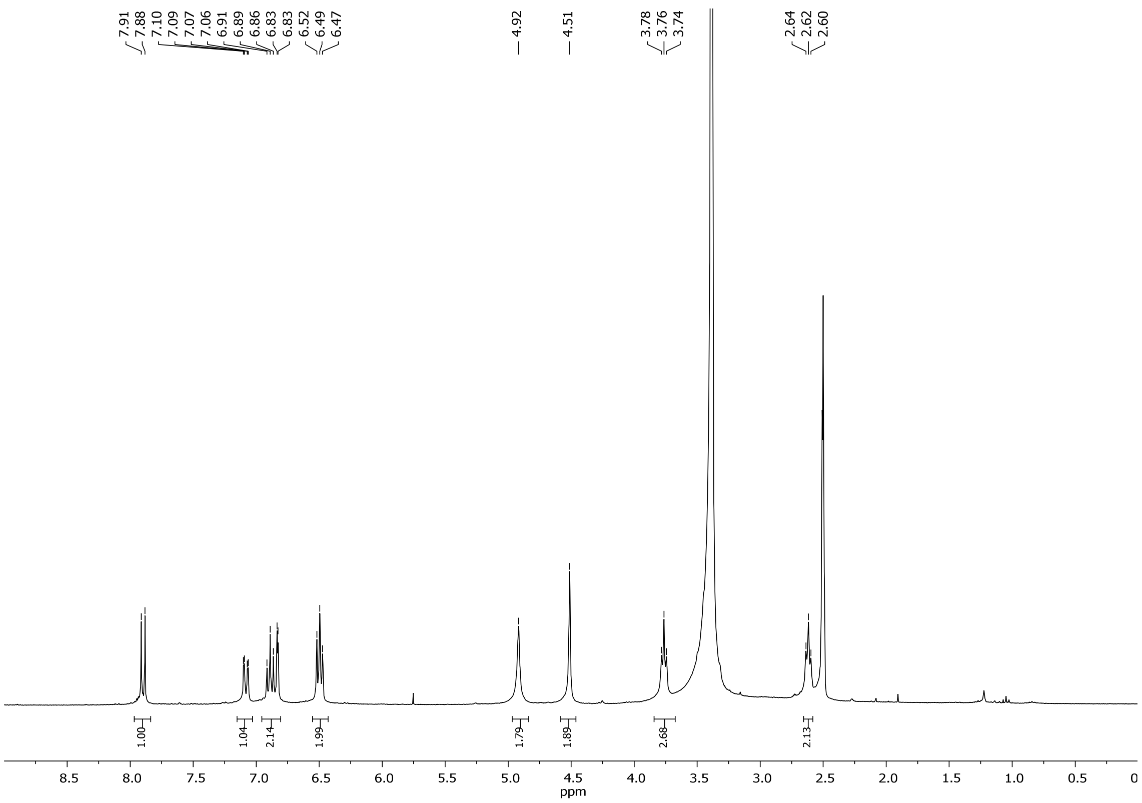

*Figure S37. ^{1}H NMR spectrum of 3,6-bis(5-amino-3,4-dihydroisoquinolin-2(1H)-yl)-9H-xanthen-9-one (**19**) (DMSO-d_6, 300 MHz).*

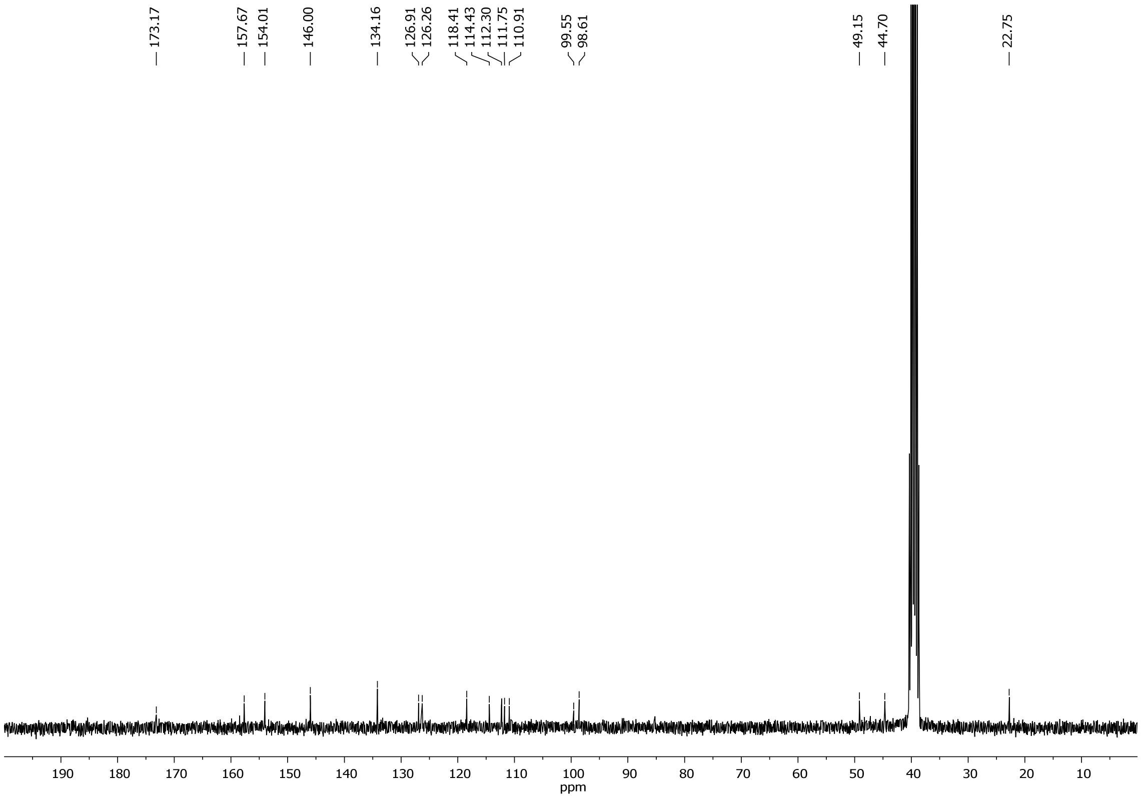

*Figure S38. ^{13}C NMR spectrum of 3,6-bis(5-amino-3,4-dihydroisoquinolin-2(1H)-yl)-9H-xanthen-9-one (**19**) (DMSO-d_6, 75 MHz).*

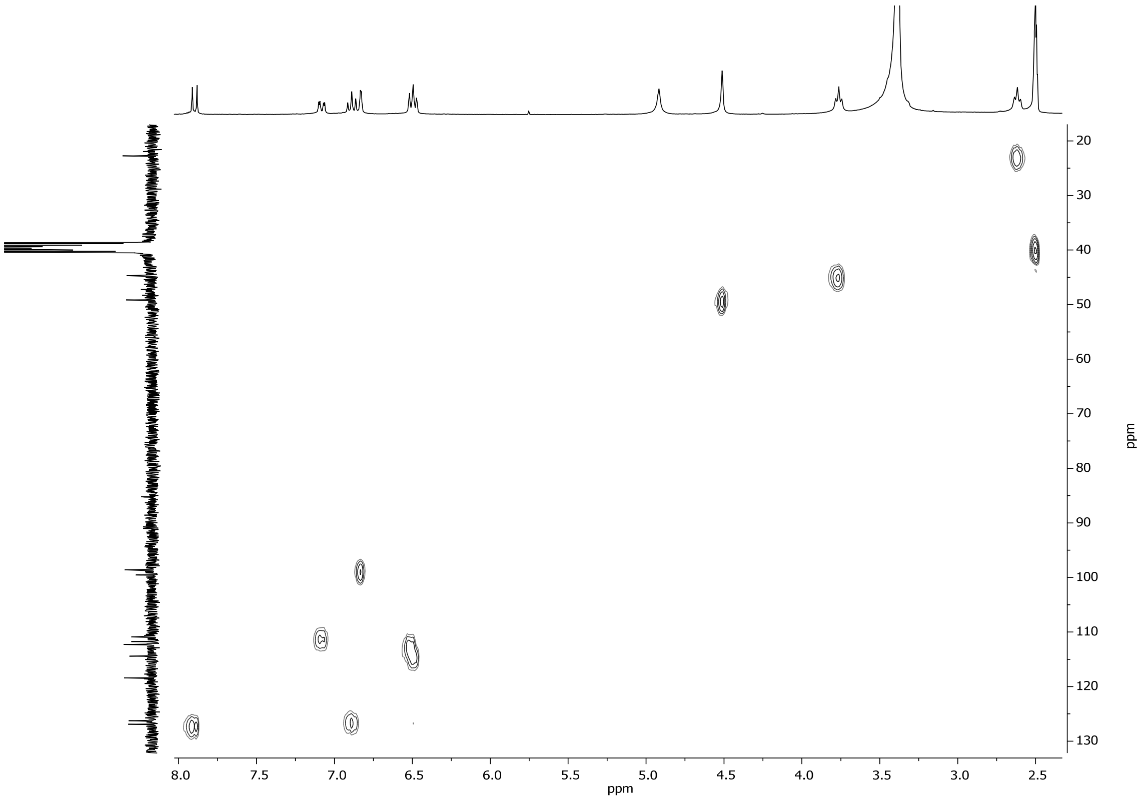

*Figure S39. HSQC spectrum of 3,6-bis(5-amino-3,4-dihydroisoquinolin-2(1H)-yl)-9H-xanthen-9-one (**19**).*

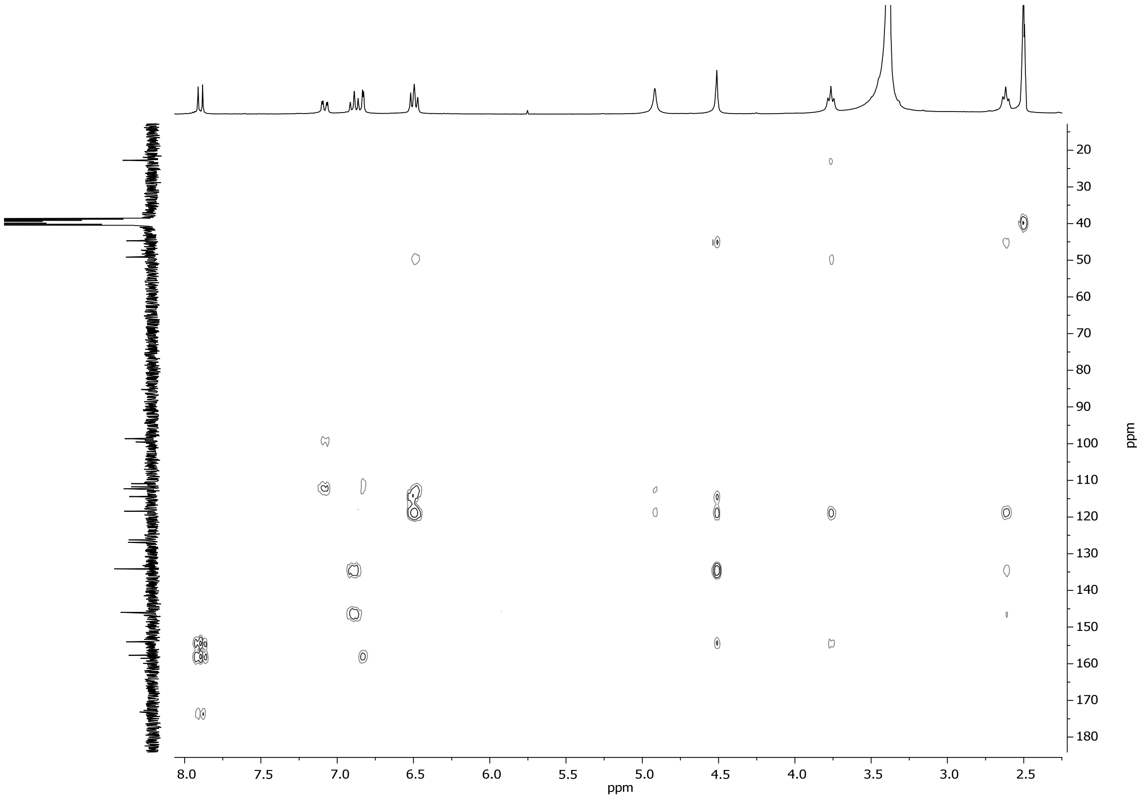

*Figure S40. HMBC spectrum of 3,6-bis(5-amino-3,4-dihydroisoquinolin-2(1H)-yl)-9H-xanthen-9-one (**19**).*

2. High-resolution mass spectrometry

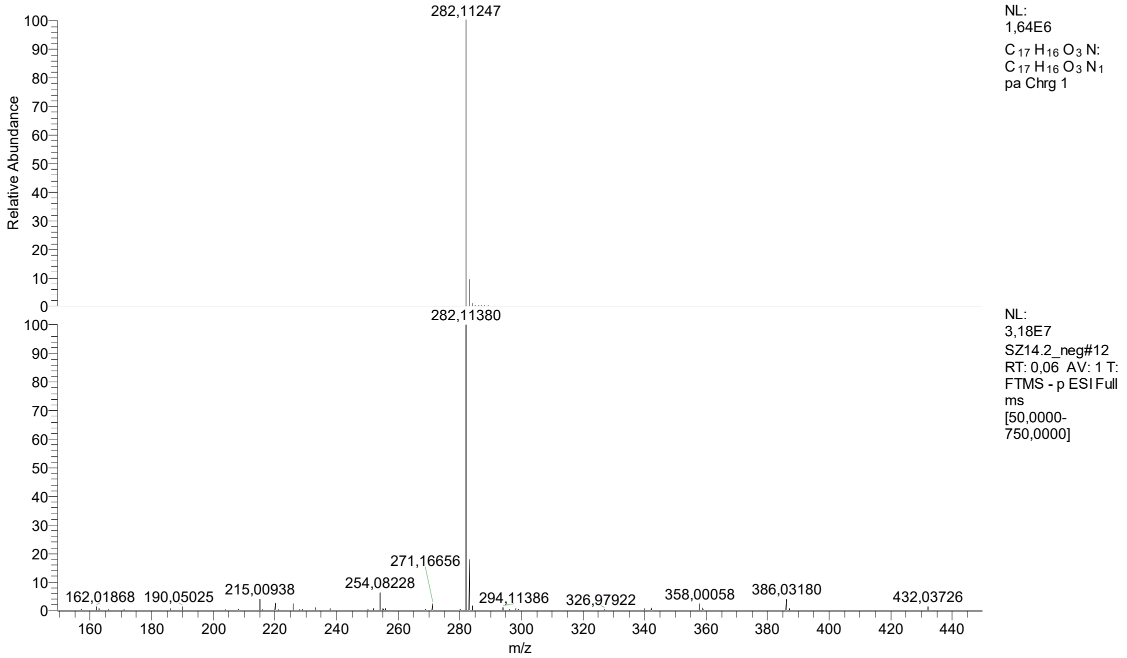

Formula	Theoretical mass	Experimental mass	Error (ppm)
[$C_{17}H_{17}NO_3$ - H^+]	282.11247	282.11380	4.71

*Figure S41. HRMS of 3-(diethylamino)-6-hydroxy-9H-xanthen-9-one (**7**).*

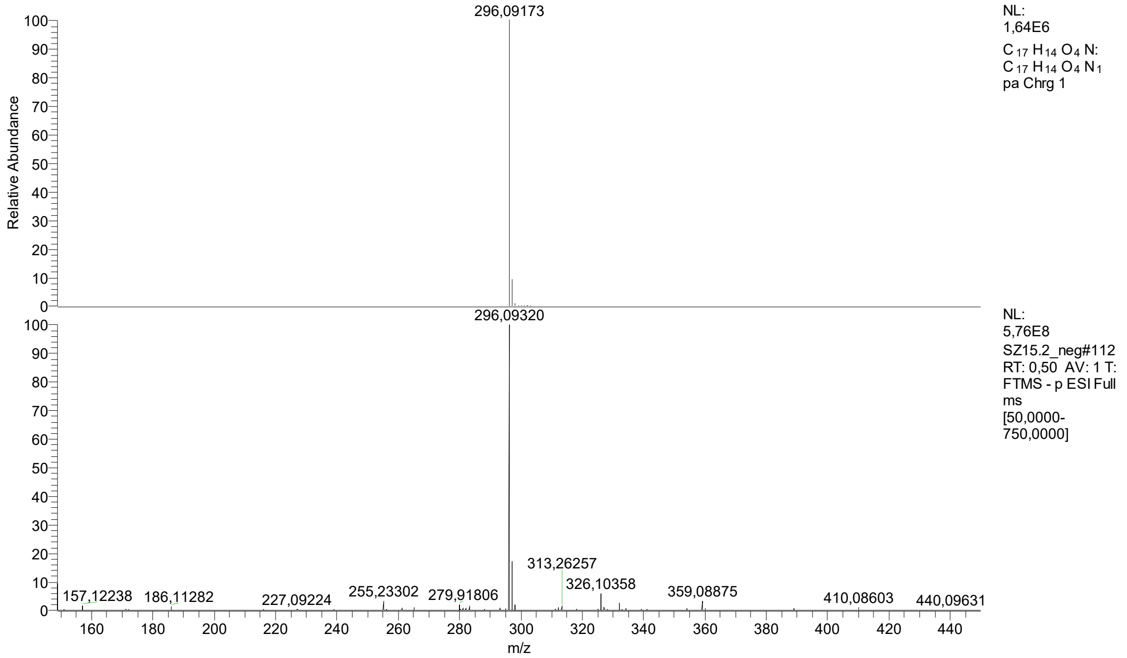

Formula	Theoretical mass	Experimental mass	Error (ppm)
[$C_{17}H_{15}NO_4$ - H^+]	296.09173	296.09320	4.96

*Figure S42. HRMS of 3-hydroxy-6-morpholino-9H-xanthen-9-one (**9**).*

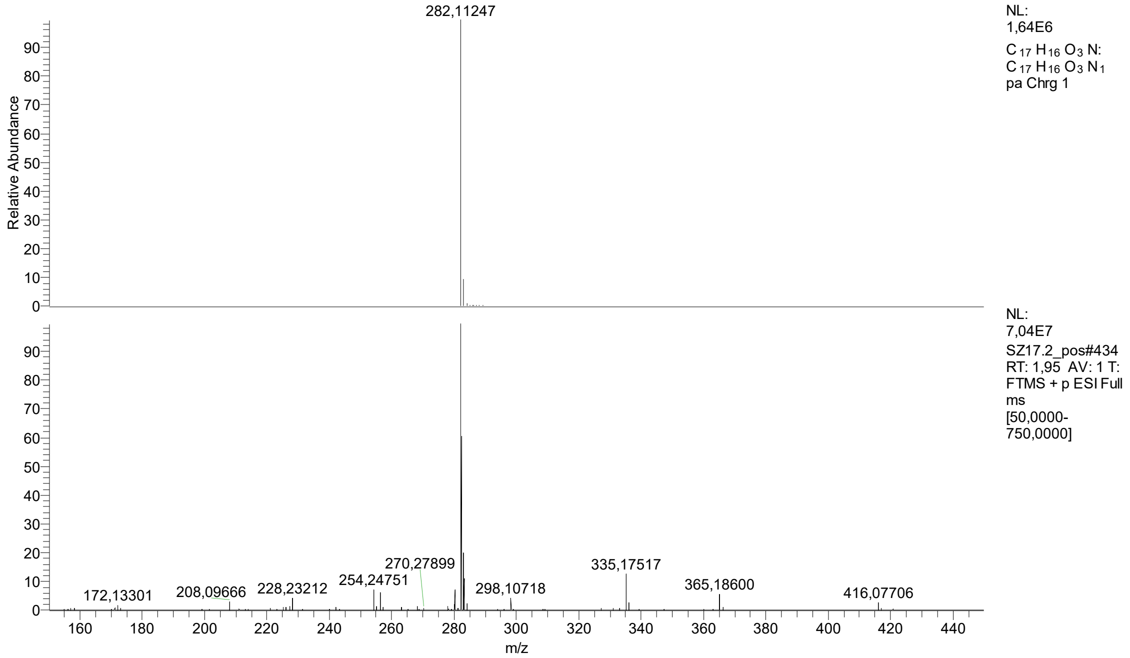

Formula	Theoretical mass	Experimental mass	Error (ppm)
$[C_{17}H_{15}NO_3 + H^+]$	282.11247	282.11230	-0.60

*Figure S43. HRMS of 3-hydroxy-6-(pyrrolidin-1-yl)-9H-xanthen-9-one (**12**).*

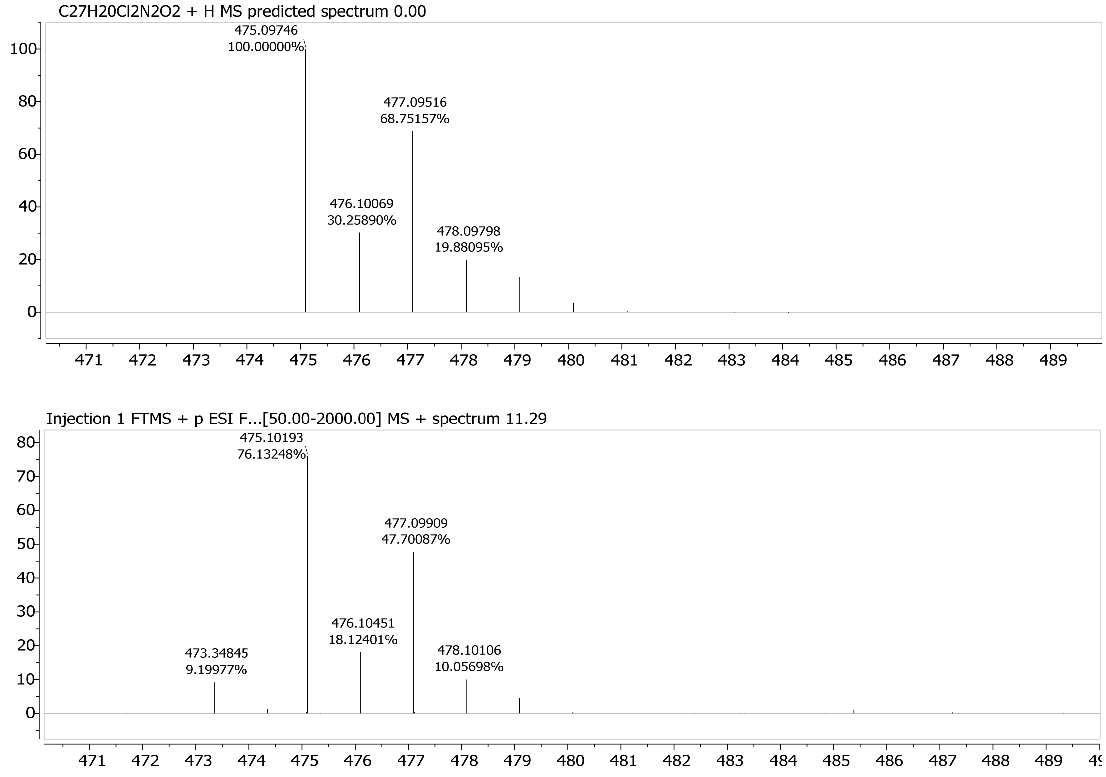

Formula	Theoretical mass	Experimental mass	Error (ppm)
$[C_{27}H_{20}Cl_2N_2O_2 + H^+]$	475.09746	475.10193	-9.41

*Figure S44. HRMS of 3,6-bis((4-chlorobenzyl)amino)-9H-xanthen-9-one (**13**).*

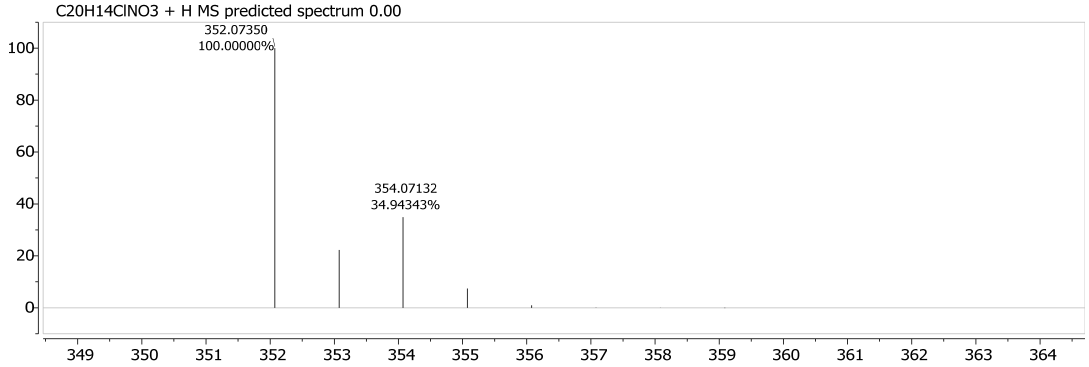

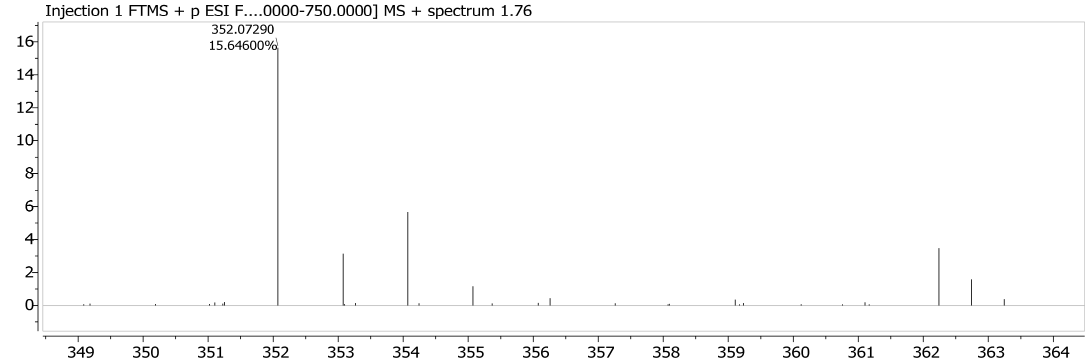

Formula	Theoretical mass	Experimental mass	Error (ppm)
[$C_{20}H_{14}ClNO_3$ + H^+]	352.07350	352.07290	-3.87

*Figure S45. HRMS of 3-((4-chlorobenzyl)amino)-6-hydroxy-9H-xanthen-9-one (**14**).*

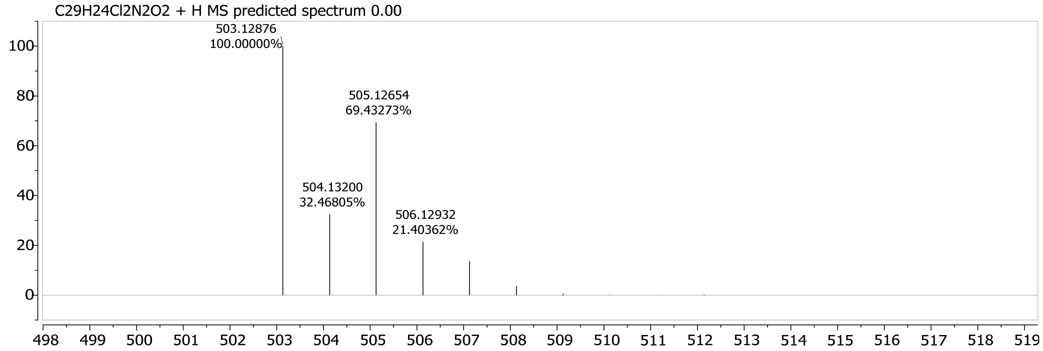

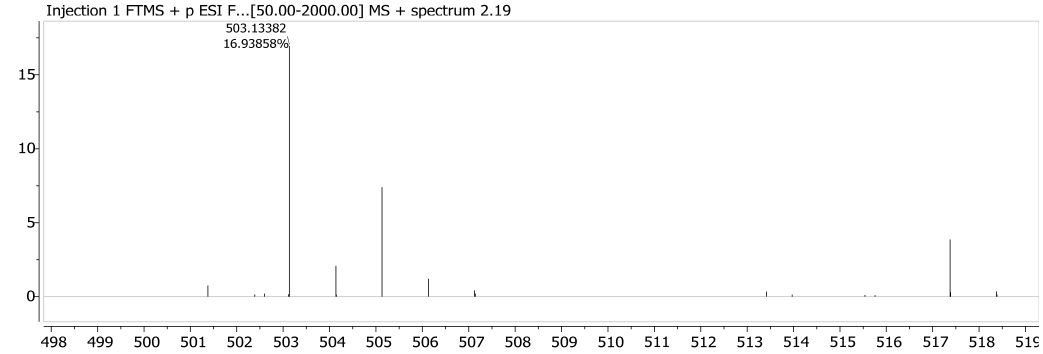

Formula	Theoretical mass	Experimental mass	Error (ppm)
[$C_{29}H_{24}Cl_2N_2O_2$ + H^+]	503.12876	503.13382	-10.06

*Figure S46. HRMS of 3,6-bis(((R)-1-(4-chlorophenyl)ethyl)amino)-9H-xanthen-9-one (**15**).*

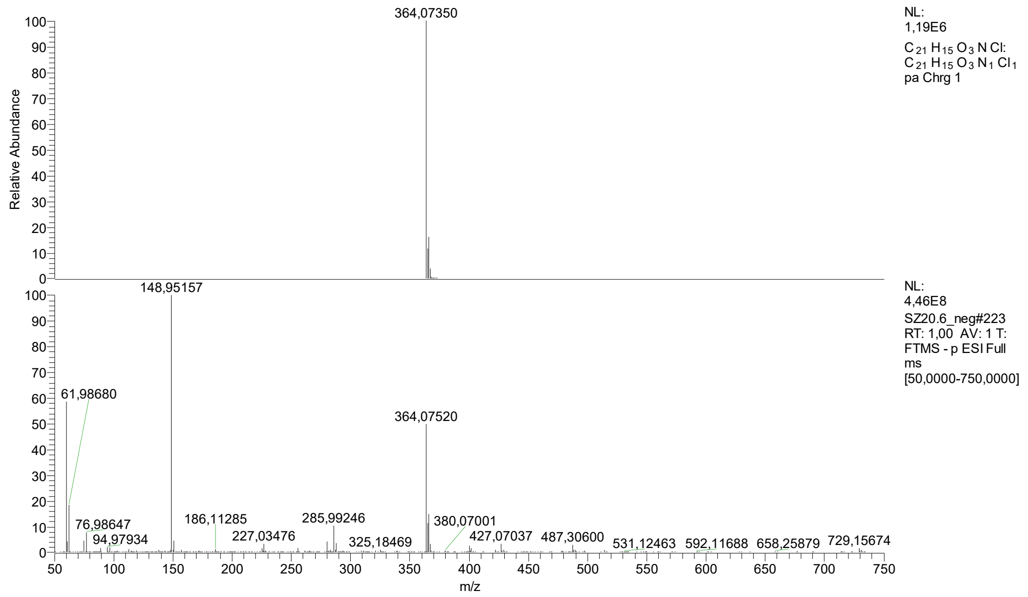

Formula	Theoretical mass	Experimental mass	Error (ppm)
[$C_{21}H_{16}ClNO_3$ - H^+]	364.07350	364.07520	4.67

*Figure S47. HRMS of (R)-3-((1-(4-chlorophenyl)ethyl)amino)-6-hydroxy-9H-xanthen-9-one (**16**).*

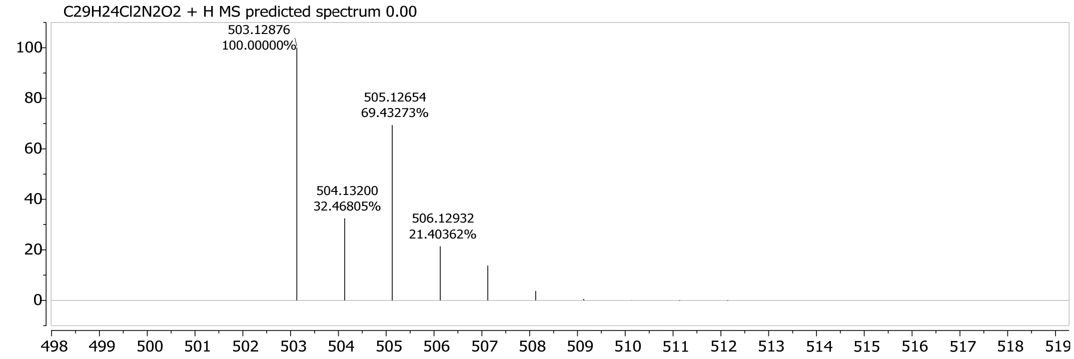

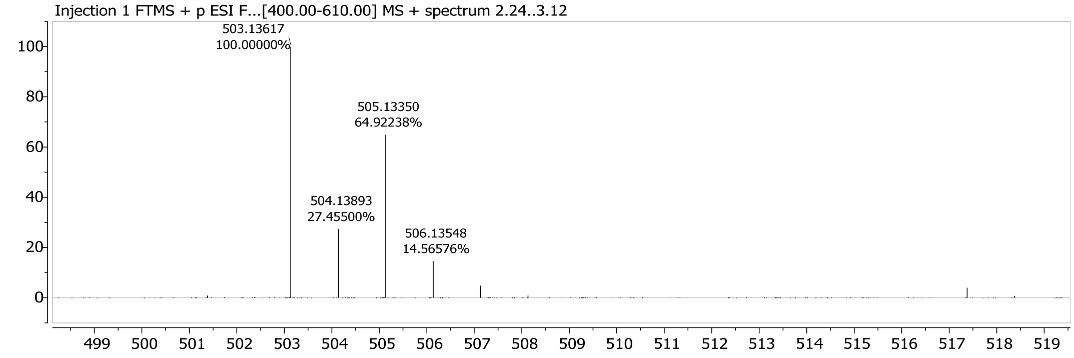

Formula	Theoretical mass	Experimental mass	Error (ppm)
[$C_{29}H_{24}Cl_2N_2O_2$ + H^+]	503.12876	503.13617	-14.73

*Figure S 48. HRMS of 3,6-bis(((S)-1-(4-chlorophenyl)ethyl)amino)-9H-xanthen-9-one (**17**).*

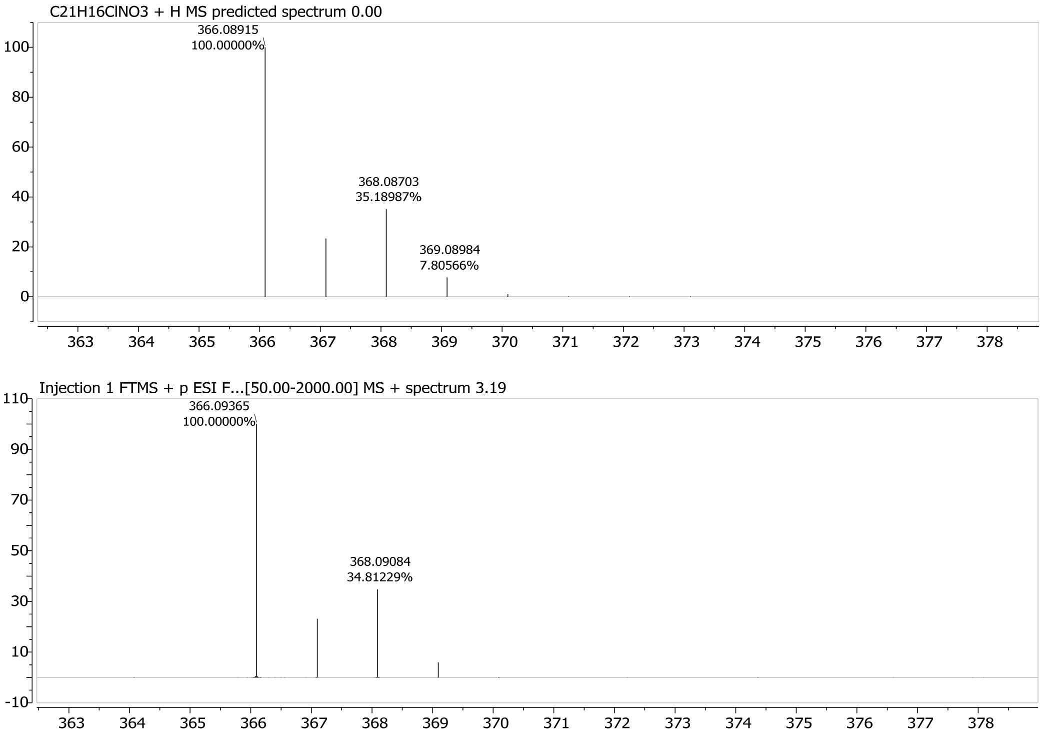

Formula	Theoretical mass	Experimental mass	Error (ppm)
[$C_{21}H_{16}ClNO_3$ + H^+]	366.08915	366.09365	-12.30

*Figure S49. HRMS of (R)-3-((1-(4-chlorophenyl)ethyl)amino)-6-hydroxy-9H-xanthen-9-one (**18**).*

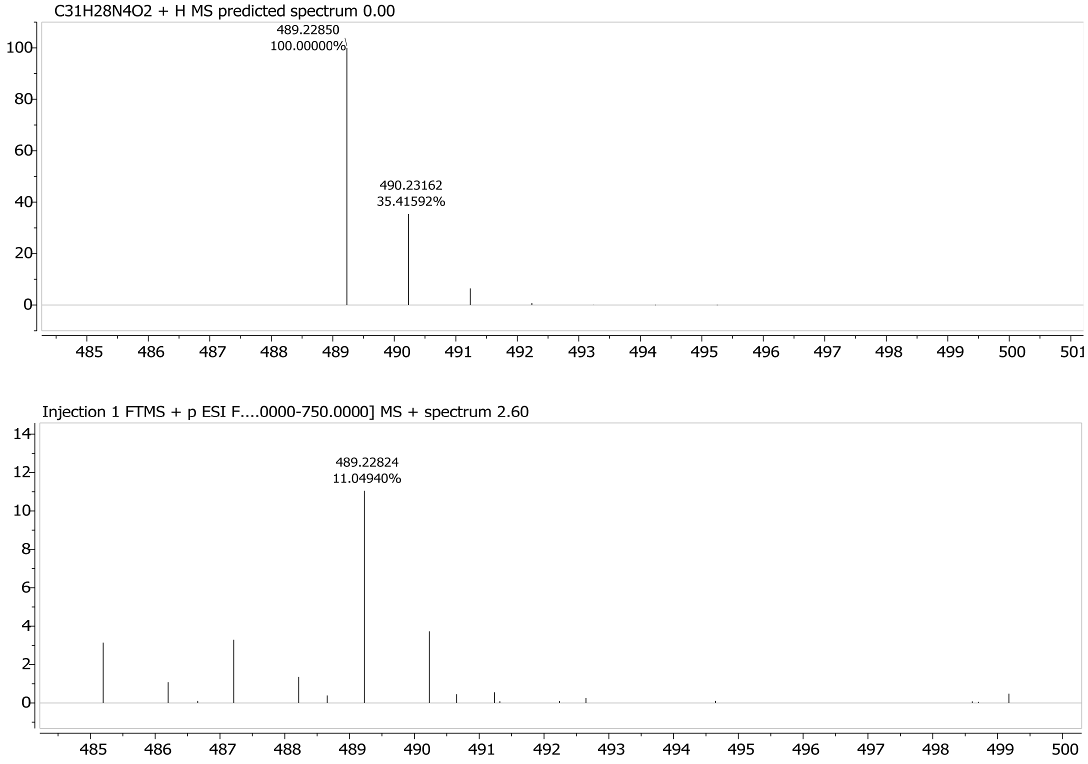

Formula	Theoretical mass	Experimental mass	Error (ppm)
[$C_{31}H_{28}N_4O_2$ + H^+]	489.22850	489.22824	0.54

*Figure S50. HRMS of 3,6-bis(5-amino-3,4-dihydroisoquinolin-2(1H)-yl)-9H-xanthen-9-one (**19**).*

3. Peak purity

Table S1. HPLC-DAD Purity Data.

Products	Peak purity index
7	99.4

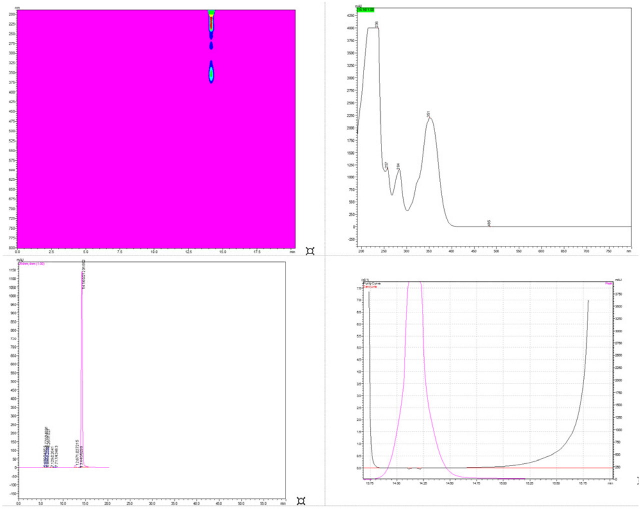

Retention Time	Area	Area %	Height
12.671	227215	1.0521	11619
14.160	21201552	98.1675	1139514
Totals	21428767	99.2196	1151133

Products	Peak purity index
9	99.9

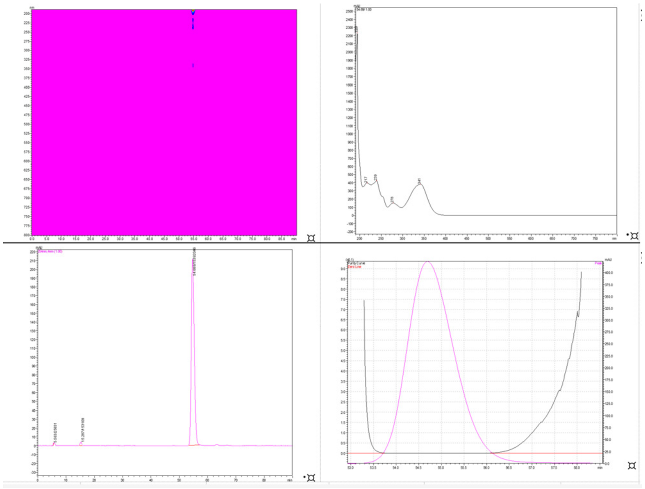

Retention Time	Area	Area %	Height
5.563	25831	0.1659	1804
15.287	153109	0.9833	4026
54.688	15392046	98.8508	211330
Totals	15570986	100	217160

12 **99.3**

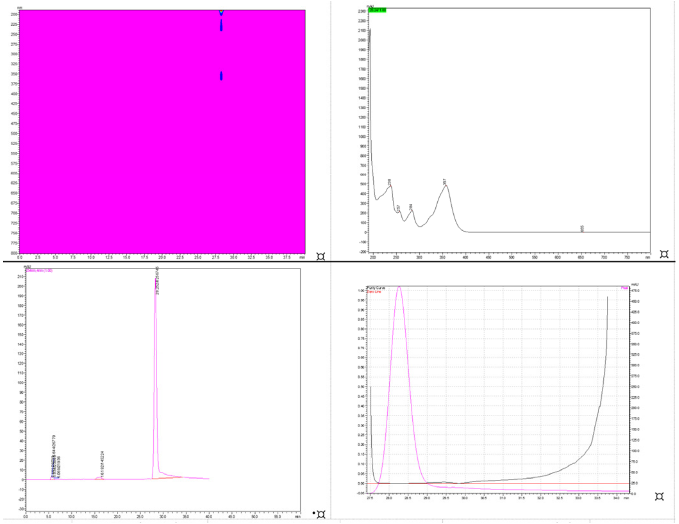

Retention Time	Area	Area %	Height
16.192	145224	1.7077	2127
28.252	8256745	97.0888	206979
Totals	8401969	98.7965	209106

13 **98.7**

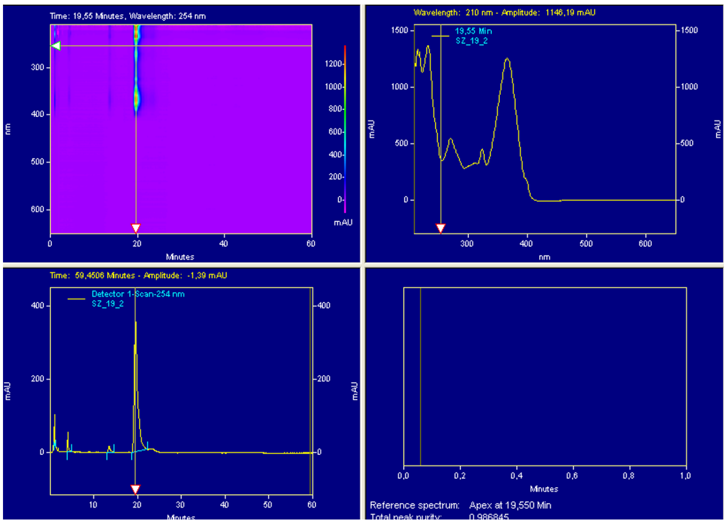

Retention Time	Area	Area %	Height
1,017	460250	2,20	81192
4,083	687567	3,29	53565
13,533	526378	2,52	15702
19,550	19205058	91,98	362302
Totals	20879253	100,00	512761

14	95.8	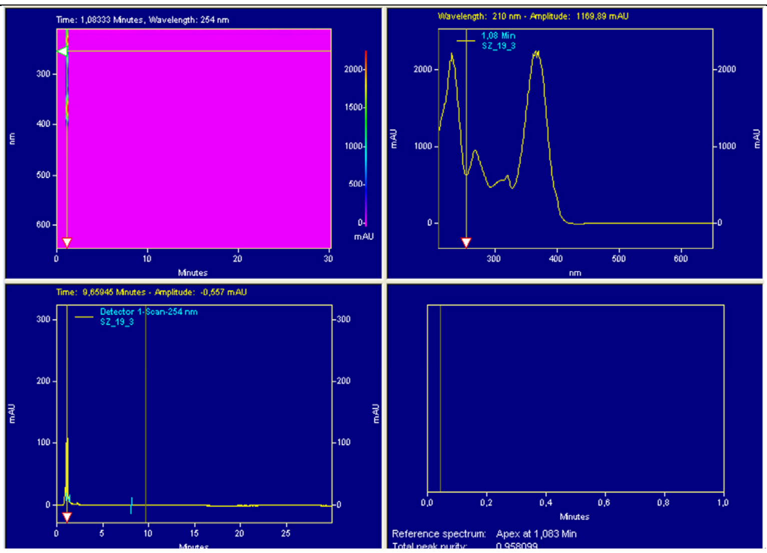

Retention Time	Area	Area %	Height
1,017	25069	0,46	2518
3,883	5475451	99,54	398719
Totals	5500520	100,00	401237

15	97.9	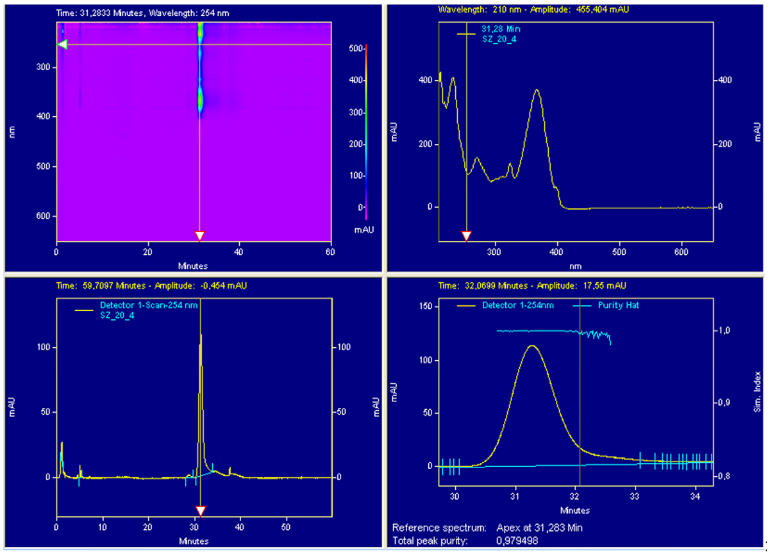

Retention Time	Area	Area %	Height
1,117	197993	3,06	16243
5,017	92670	1,43	9485
28,733	110239	1,70	2273
31,283	6070023	93,80	111771
Totals	6470925	100,00	139772

**16**	**99.9**	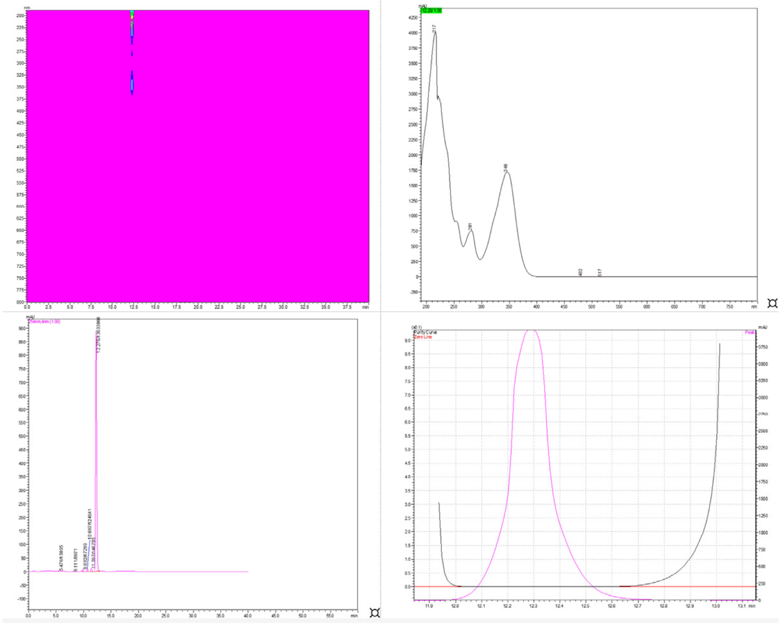

Retention Time	Area	Area %	Height
11.397	146705	1.0466	11011
12.278	13633866	97.2649	888585
Totals	13780571	98.3115	899596

**17**	**96.1**	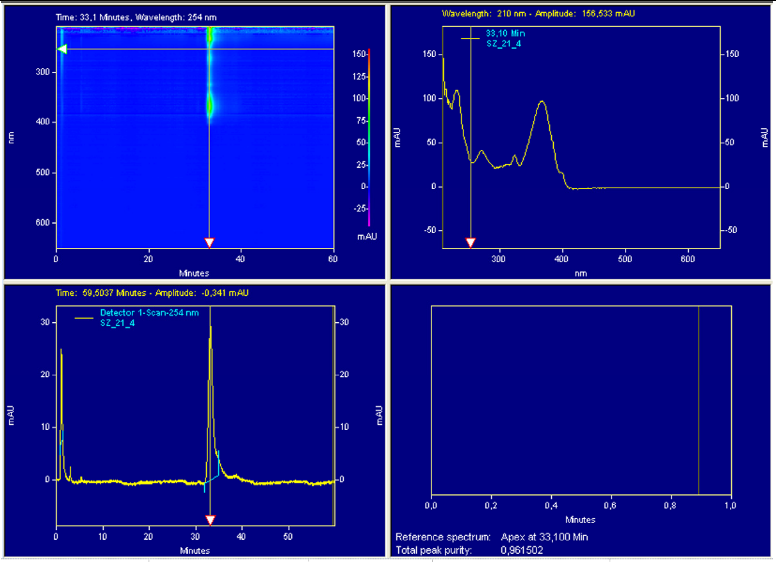

Retention Time	Area	Area %	Height
0,950	263129	11,35	18254
33,100	2055302	88,65	29795
Totals	2318431	100,00	48049

18 98.2

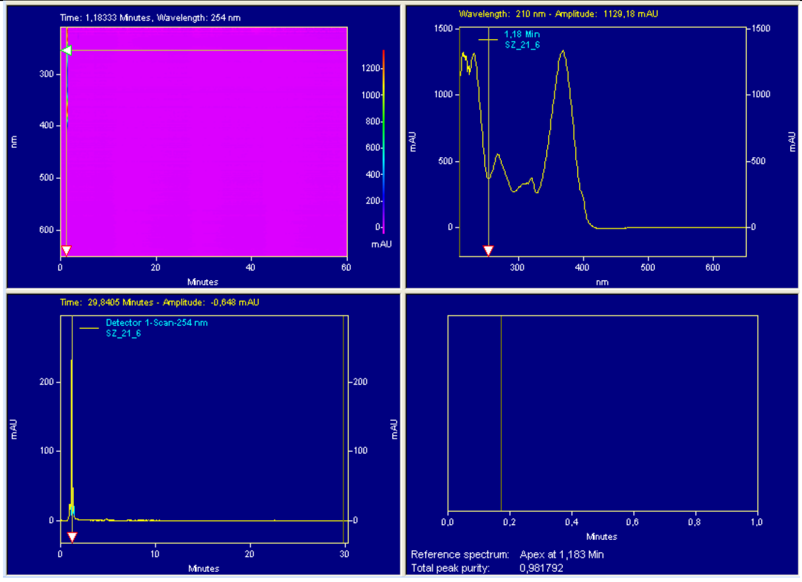

Retention Time	Area	Area %	Height
1,183	1532501	100,00	354977
Totals	1532501	100,00	354977

19 95.5

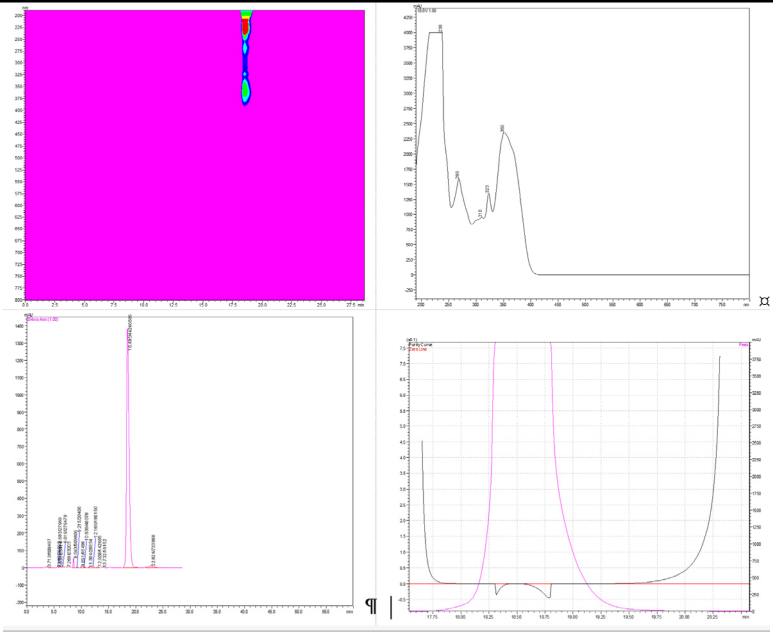

Retention Time	Area	Area %	Height
18.485	44266586	95.1867	1382397
22.824	733868	1.5780	9960
Totals	45000454	96.7647	1392357
